# Method comparison for *N*-glycan profiling: Towards the standardization of glycoanalytical technologies for cell line analysis

**DOI:** 10.1371/journal.pone.0223270

**Published:** 2019-10-07

**Authors:** Maximilianos Kotsias, Athanasios Blanas, Sandra J. van Vliet, Martina Pirro, Daniel I. R. Spencer, Radoslaw P. Kozak

**Affiliations:** 1 Ludger Ltd., Culham Science Centre, Abingdon, Oxfordshire, England, United Kingdom; 2 Amsterdam UMC, Vrije Universiteit Amsterdam, Molecular Cell Biology and Immunology, Cancer Center Amsterdam, Amsterdam, The Netherlands; 3 Leiden University Medical Centre, Centre for Proteomics and Metabolomics, Leiden, The Netherlands; Swiss Institute of Bioinformatics, SWITZERLAND

## Abstract

The study of protein *N*-glycosylation is essential in biological and biopharmaceutical research as *N*-glycans have been reported to regulate a wide range of physiological and pathological processes. Monitoring glycosylation in diagnosis, prognosis, as well as biopharmaceutical development and quality control are important research areas. A number of techniques for the analysis of protein *N*-glycosylation are currently available. Here we examine three methodologies routinely used for the release of *N*-glycans, in the effort to establish and standardize glycoproteomics technologies for quantitative glycan analysis from cultured cell lines. *N*-glycans from human gamma immunoglobulins (IgG), plasma and a pool of four cancer cell lines were released following three approaches and the performance of each method was evaluated.

## Introduction

*N*-linked glycosylation is one of the most common post-translational modifications of proteins.[1] *N*-glycans have been reported to regulate a multitude of biological processes such as ligand-receptor interactions, immune response, protein secretion and transport.[2–8] They have also been reported to play fundamental roles in disease and alterations in glycosylation have been observed in cancer, Alzheimer’s disease, inflammatory conditions and in several congenital disorders of glycosylation (CDGs).[8–18] Protein *N*-glycosylation is also critical for the development of biopharmaceuticals, influencing efficacy, immunogenicity, stability and pharmacokinetics.[8,19–21]. Therefore, establishing connections between glycan structures and their functions, elucidating molecular mechanisms involved in pathogenesis, monitoring glycosylation in diagnosis, prognosis as well as biopharmaceutical development and quality control in the context of existing and potential drugs that are glycosylated are important research areas.[22]

Many studies into glycosylation changes in cancer have been performed with *ex vivo* tissue, such as the analysis of mucus, blood, or epithelial cells. Many other investigative approaches utilise *in vivo* animal models or *in vitro* established cell lines. In contrast to the limited availability of tumour tissue samples and primary cells, the availability of *in vitro* models makes cell lines far more suitable for extensive analyses. This and other properties[23–25] demonstrate the essential role that *in vitro* cell line models have as a useful tool for studying biological mechanisms in disease.

As no universal method exists for the rapid and reliable identification of glycan structures, scientists must be aware of the available techniques and savvy in selecting the best method or combination of methods for glycan analysis depending on the information they wish to learn.[22] Various approaches are currently employed for the relative or absolute quantitation of *N*-linked glycans in biological samples. These analysis techniques often result in varied glycan profiles, which is probably the consequence of variations in the pre-processing sample preparation methodologies.

A number of techniques for the analysis of protein *N*-glycosylation are currently available and have been devised based on either an enzymatic or chemical approach.[26]

The enzymatic release of *N*-linked oligosaccharides from glycoproteins is most commonly achieved with the use of the endoglycosidic enzyme PNGaseF (*N*-glycosidase F). PNGaseF cleaves all asparagine-linked complex, hybrid, or high mannose oligosaccharides with the exception of specific *N*-glycans that contain fucose α(1, 3) linked to the asparagine-linked *N*-acetylglucosamine (GlcNAc), commonly found in glycoproteins from plants or parasitic worms. PNGaseA (*N*-glycosidase A), isolated from almond meal, must be used in this situation.[26,27]

More than one adaptations of this technique are regularly used, including an in-solution PNGaseF[26] and a PVDF membrane-based protocol, which can provide benefits such as the subsequential release and analysis of *N*- and *O*-glycans from the same protein spot.[28–30]

Amongst chemical methods, hydrazine is the most commonly used for the complete release of *N*-linked oligosaccharides. It is a well-established and validated technique.[26]

Here we provide an overview of the methodologies routinely employed for the release of *N*-linked glycans, highlighting the information that can be obtained from each and when they might be best used. In addition to the qualitative and quantitative comparative analyses of the approaches mentioned above, one important task is to make a step towards the establishment and standardization of glycoanalytical technologies aimed at the *N*-glycan profiling of cultured cell lines.

We used human IgG and human plasma as system suitability and process controls and HT29, HCT15, HCT116 and KM12 human colorectal cancer cell lines to evaluate three different approaches for *N*-glycan release, recovery and their analysis on liquid chromatography-mass spectrometry (LC-MS) system. The performance of each approach was evaluated on the basis of a number of criteria such as execution time, ultra-high performance liquid chromatography (UHPLC) profiles obtained, *N*-glycan identification and characterization ability, reproducibility of the data generated and high-throughput potential.

The comparison of the three approaches for *N*-glycan profiling and identification by UHPLC and electrospray ionisation mass spectrometry (ESI-MS) revealed useful information towards the establishment and standardization of glycoanalytical technologies for quantitative glycan analysis from cultured cell-lines.

## Materials and methods

### Materials

All reagents and kits for *N*-glycan release, labelling and clean-up were obtained from Ludger Ltd. (Oxford, UK). The 1.5 mL Eppendorf^®^ Safe-Lock microcentrifuge tubes, the Parafilm^®^ M sealing film and methanol (MeOH) were obtained from Sigma (Dorset, UK). Acetonitrile (Romil; 190 SPS for UV/gradient quality) and ethanol were obtained from Charlton Scientific (Charlton, Oxon, UK). The trifluoroacetic acid (TFA) and the 5 mL Chromacol vials were purchased from Thermo (Hampshire, UK). The polypropylene collection plates and the silicone plate lids were purchased from 4titude (Surrey, UK). The ultrasonic bath (Bandelin Sonorex Digitec DT103H) was purchased from Ultraschall-Welt (Morfelden-Walldorf, Germany). The heating block (DB-2A) was purchased from Techne (Staffordshire, UK). The horizontal shaker (Mini Orbital Shaker SO5) was purchased from Stuart Scientific (Staffordshire, UK). Human IgG and human plasma were obtained from Ludger Ltd. (Oxford, UK). One batch of HT29 human colorectal cancer cell line was obtained from the Center for Proteomics and Metabolomics of the Leiden University Medical Centre (Leiden, The Netherlands). HT29 (second batch), HCT15, HCT116 and KM12 human colorectal cancer cell lines were obtained from Amsterdam UMC, Vrije Universiteit Amsterdam, Molecular Cell Biology and Immunology, Cancer Center Amsterdam (Amsterdam, The Netherlands). Samples were dried down in a Thermo Savant centrifugal evaporator from Thermo (Hampshire, UK).

### Release of *N*-glycans from glycoprotein

Release of *N*-glycans from human IgG, plasma and the HT29, HCT15, HCT116 and KM12 human colorectal cancer cell lines was performed manually following three approaches; in-solution PNGaseF, polyvinylidene difluoride (PVDF)-membrane based PNGaseF and *N*-mode hydrazinolysis. Three technical replicates of human IgG, human plasma and human cancer cell lines were taken through each protocol.

### In-solution PNGaseF protocol

The *N*-glycans were released from glycoproteins using LudgerZymeTM PNGaseF Release Kit (LZ-rPNGaseF-kit) (Ludger Ltd.). Briefly, cell pellets (~2 x 10^6^ cells/technical replicate) were suspended in 100 μL of water with resistivity 18.2 MΩ and homogenized for 60 min in a sonication bath alongside human IgG (100 μg/technical replicate), human plasma (2 μL/technical replicate) and pure water (9 μL) as negative control. A thin layer of Parafilm^®^ M sealing film was applied to each Eppendorf^®^ tube prior to their sonication in the water bath in order to prevent contamination. Following the sonication step, cancer cell lines and human IgG samples were dried down in a centrifugal evaporator. Sample volumes were corrected to 9 μL with pure water and 1 μL of denaturing buffer (5% SDS 500 mM DTT) was added to each sample and mixed. The samples were incubated for 10 min at 100°C in order to denaturate the proteins and allowed to cool down at room temperature. 2 μL of 10% NP-40 solution, 6 μL of pure water and 1 μL of PNGaseF were added to each sample. Samples were vortexed and incubated overnight at 37°C. Released *N*-glycans were converted to aldoses with 0.1% formic acid, filtered through a 96-well protein binding plate and dried down completely (Ludger Ltd.).

### PVDF membrane-based PNGaseF protocol

Sample preparation was performed as described in the previous paragraph. Release of *N*-glycans was performed using a PVDF membrane-based protocol adapted from Burnina *et*.*al*.[28,29] Following the sample sonication step, sample volumes were corrected to 100 μL with pure water. A 96-well protein binding plate was placed onto the vacuum manifold. 200 μL of 70% ethanol in water were added to each well of the protein binding plate to be used. A cycle of vacuum (50 mbar below atmospheric pressure) was applied to aid washing of the wells. 50 μL of glycobuffer (1X) was added to each well of the protein binding plate followed by another cycle of vacuum to aid liquid elution. Following these steps, each sample was transferred into a well, followed by the addition of 7.5 μL of denaturation buffer. The protein binding plate was incubated at 60°C for 30 min in a moisturized sealed box as an incubation chamber within an oven. The plate was shaken for 5 min on a horizontal shaker prior to centrifugation (1 min, 500 x g). The wells were washed with 200 μL of mQ water and incubated for 3 min on a horizontal shaker prior to centrifugation (1 min, 500 x g). This procedure was repeated once more. Additional 50 μL of glycobuffer (1X) was added to each well and the plate was incubated for 3 min on a horizontal shaker prior to centrifugation (1 min, 500 x g). Residual solvent from the bottom of the protein binding plate filter was removed by gently dipping on a clean tissue. Following this step, the protein binding plate was placed on top of a clear 96-well collection plate and incubated at 37°C with 1 μL PNGaseF in the presence of 2 μL glycobuffer 2 (10X), 2 μL of NP-40 (10%) and 16 μL mQ water. The protein binding plate was sealed using a silicone plate lid prior to every incubation and mixing steps. Glycans were recovered into the 96-well collection plate by centrifugation (2 min, 1000 x g). Eventual residual solution was collected from the membrane by washing each well of the protein binding plate with 100 μL of mQ water prior to centrifugation (2 min, 1000 x g). This procedure was repeated twice. Finally, samples were transferred into Eppendorf^®^ tubes and dried down in a centrifugal evaporator. Released *N*-glycans were converted to aldoses with 0.1% formic acid, filtered through a 96-well protein binding plate and dried down completely (Ludger Ltd.).

### *N*-mode hydrazinolysis protocol

Sample preparation was performed as previously described. Following the sonication step, each sample was transferred into a 5 mL Chromacol vial and dried down completely. The *N*-glycans were released by the addition of hydrazine (0.5 mL) and incubation at 100°C for 5 h on a heating block.[31] Hydrazine was removed by centrifugal evaporation. The samples were placed on ice for 20 min (4°C) and were re-*N*-acetylated by the addition of 1 M sodium bicarbonate solution (450 μL) and acetic anhydride (21 μL). Samples were vortexed and incubated at 4°C on a horizontal shaker for 60 min. Residual acetohydrazide derivatives were converted to unreduced glycans by the addition of 600 μL of 5% trifluoroacetic acid (TFA) and incubation at 4°C for 60 min. Released *N*-glycans were purified and recovered by passing them through Ludger-Clean^TM^ EB10 Glycan Cleanup Cartridges (LC-EB10-A6) as stated in the product guide (Ludger Ltd.). Eluates were dried by centrifugal evaporation.

### Fluorescent labelling

Released *N*-glycans were fluorescently labelled by reductive amination with procainamide using LudgerTag^TM^ Procainamide Glycan Labelling Kit (LT-KPROC-24) (Ludger Ltd.). Briefly, samples in 10 μL of pure water were incubated for 60 min at 65°C with procainamide labelling solution.

### Purification of procainamide labelled glycans

The procainamide labelled *N*-glycans were cleaned up using a Ludger-Clean^TM^ Procainamide Clean-up Plate (LC-PROC-96) (Ludger Ltd.). The purified procainamide labelled *N*-glycans were eluted with pure water (200 μL). The samples were dried by centrifugal evaporation and resuspended in pure water (100 μL) for further analysis.

### LC-ESI-MS and MS/MS analysis

Procainamide labelled samples and system suitability standards were analysed by hydrophilic interaction liquid chromatography (HILIC)-(U)HPLC-ESI-MS with fluorescence detection. 2 μL of aqueous solution of each sample was injected onto an ACQUITY UPLC^®^ BEH-Glycan 1.7 μm, 2.1 x 150 mm column (Waters) at 40°C on a Ultimate 3000 UHPLC instrument with a fluorescence detector (λex = 310 nm, λem = 370 nm) (Thermo), attached to a Bruker Amazon Speed electron-transfer dissociation (ETD). The running conditions used were: Solvent A was 50 mM ammonium formate pH 4.4 made from Ludger Stock Buffer, and solvent B was acetonitrile. Gradient conditions were: 0 to 10 min, 76 to 76% B at a flow rate of 0.4 mL/min; 10 to 85 min, 76 to 51% B at a flow rate of 0.4 mL/min; 85 to 89 min, 51 to 10% at a flow rate of 0.2 mL/min; 89 to 93 min, 10 to 76% at a flow rate of 0.2 mL/min; 93 to 95 min, 76 to 76% at a flow rate of 0.4 mL/min. The Amazon Speed settings used were: source temperature 250°C, gas flow 10 L/min; Capillary voltage 4500 V; ICC target 200,000; max accu time 50.00 ms; rolling average 2; number of precursor ions selected 3, release after 1.0 min; Positive ion mode; Scan mode: enhanced resolution; mass range scanned, 300–1700; Target mass, 657.28.

A procainamide labelled glucose homopolymer ladder (GHP) was used as a system suitability standard as well as an external calibration standard for glucose units (GU) allocation. ESI-MS and tandem mass spectrometry (MS/MS) data analysis was performed using Bruker Compass Data Analysis software V4.4. Glycan structures/composition were assigned using the database of GU values and confirmed by a MS and MS/MS fragmentation.[32] Further verification by exoglycosydases digestion may be needed for exact structural assignments. Glycan structures were visualized using GlycoWorkBench, version 2.1.[33] Structures for glycans are depicted following the Consortium for Functional Glycomics (CFG) notation: *N*-acetylglucosamine (N; blue square), fucose (F; red triangle), *N*-acetylgalactosamine (N; yellow square), galactose (H; yellow circle), glucose (H; blue circle), *N*-acetylneuraminic acid (S; purple diamond), *N*-glycolylneuraminic acid (Sg; light-blue diamond).[34]

## Results and discussion

In this study, we compared the performance of three different approaches for *N*-glycan release, recovery, and their analysis using LC-MS. We started by using human IgG (system suitability standard and process control) and human plasma (process control) as a model glycoprotein and the HT29 human colorectal cancer cell line. From this point of the manuscript we will refer to this part of the work as “experiment 1”.

Four additional human colorectal cancer cell lines (HT29, HCT15, HCT116 and KM12) from a different culture batch, alongside a new set of system suitability and process controls (human IgG and plasma) were tested to evaluate the consistency of the results obtained from experiment 1. From this point of the manuscript we will refer to this part of the work as “experiment 2”.

The intermediate precision of each analytical procedure used was evaluated by comparing the data obtained from the release of *N*-glycans from human IgG processed in experiment 2, to those generated by the release of *N*-glycans from human IgG processed in experiment 1.

Glycan areas from triplicate samples of human IgG (system suitability and process control) were integrated and standard deviations (SDs) and coefficients of variation (CVs) were calculated for released *N*-glycans with relative areas (RAs) ≥ 0.91%. The variation for average relative area values for human IgG *N*-glycans prepared and analysed in two different days (3 samples from experiment 1 vs 3 samples from experiment 2) produced CVs < 26.33 for *N*-glycans with RAs ≥ 2.14% indicating a high level of similarity between the two data sets for all the methods used. These results can be appreciated in Table A of the supporting information (S1 File).

Since the batch of human plasma used in experiment 2 was different from the one used in experiment 1, the data obtained from the release of *N*-glycans from this sample was only used to assess the efficiency of the methods and for structural assignment purposes.

All methods were compared under the optimum conditions previously described and no additional optimization was performed since this was not the scope of this study.

**Fig 1:**
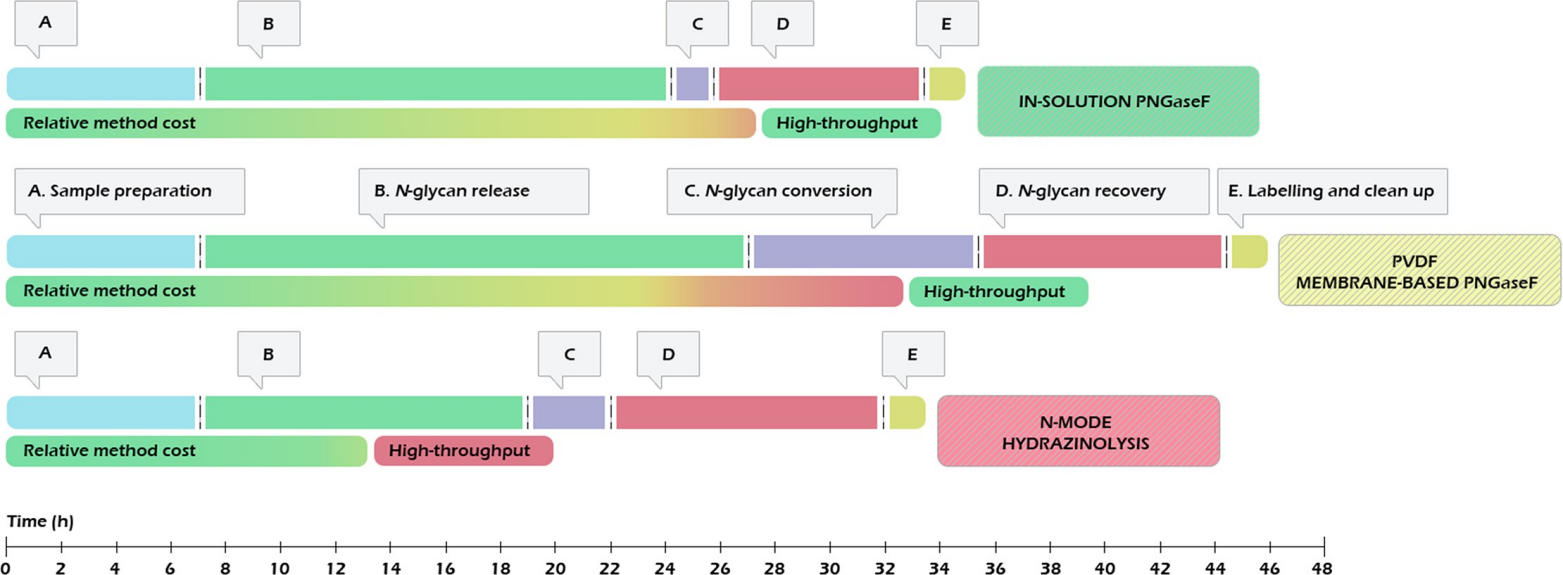
Visual representation of the workflows for in-solution PNGaseF, PVDF membrane-based PNGaseF and *N*-mode hydrazinolysis, highlighting their time, cost and high-throughput potential.

The time spent for sample preparation, *N*-glycan release, recovery and fluorescent labelling using each approach are summarized in Table B in S1 File. A visual representation of the workflows for in-solution PNGaseF, PVDF membrane-based PNGaseF and *N*-mode hydrazinolysis, highlighting their time, cost and high-throughput potential is presented in Fig 1.

Overall, the hydrazine release proved to be the fastest protocol, outperforming the in-solution PNGaseF and the PVDF membrane-based PNGaseF methods in terms of time needed to prepare and measure glycan samples by respectively 2 and 11.5 hours. While the in-solution PNGaseF and the hydrazine methods had similar execution times, the high number of centrifugation cycles required during the release of *N*-glycans using the PVDF membrane-based PNGaseF method resulted in larger overall procedure duration. This parameter needs to be carefully taken into consideration in terms of sample throughput, especially when large sample sets need to be analysed.

**Fig 2:**
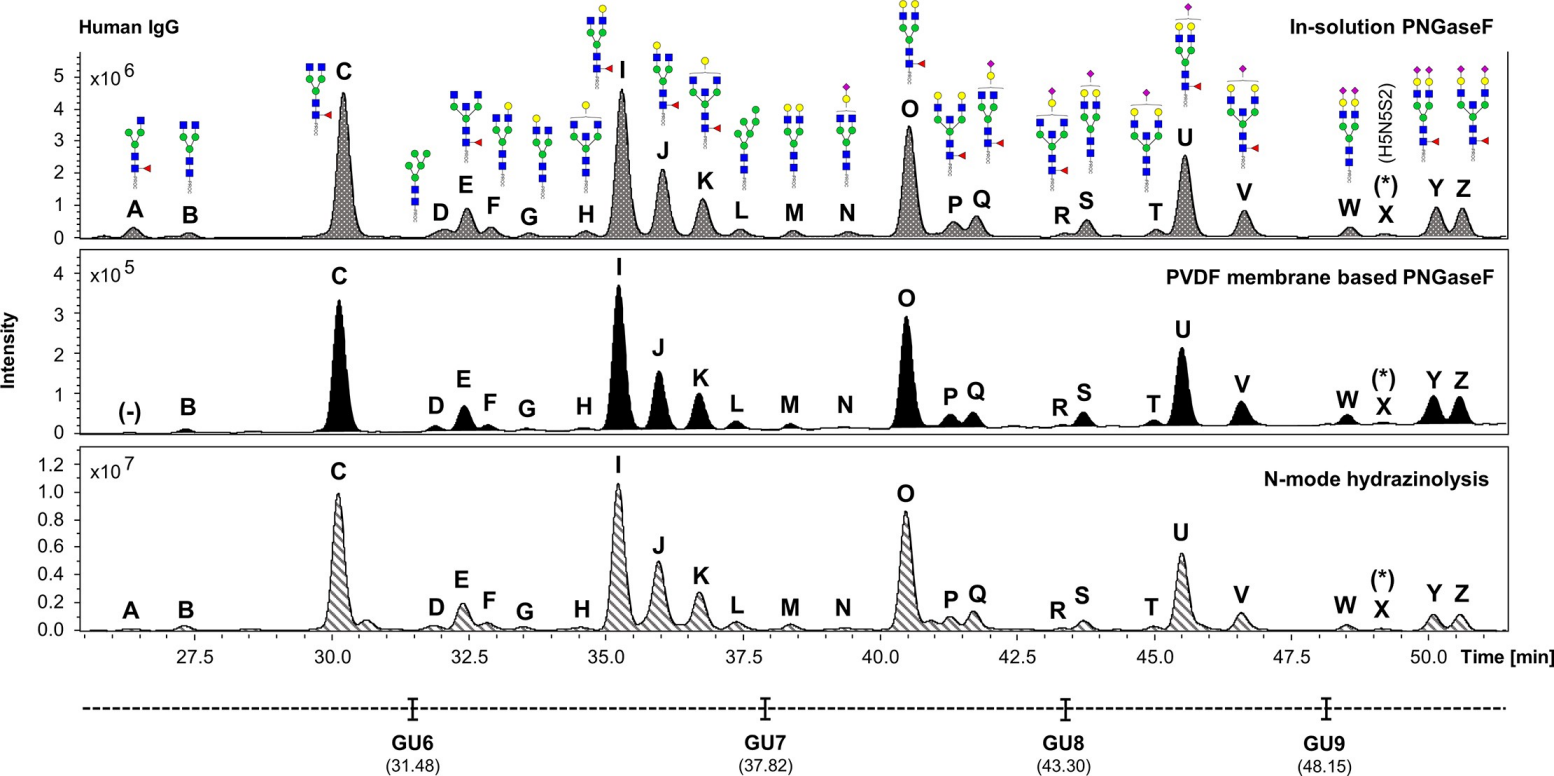
Comparison of the FLR chromatograms for human IgG *N*-glycans released by in-solution PNGaseF, PVDF membrane-based PNGaseF and *N*-mode hydrazinolysis. *N*-glycan structures, illustrated by cartoons, were assigned by MS and MS/MS fragmentation analysis. (-) Missing structures. (*) No MS/MS data detected for structural confirmation. Glycan compositions are given in the terms of hexose (H), *N*-acetylhexosamine (N), deoxyhexose (F), *N*-acetylneuraminic acid (S).

**Fig 3:**
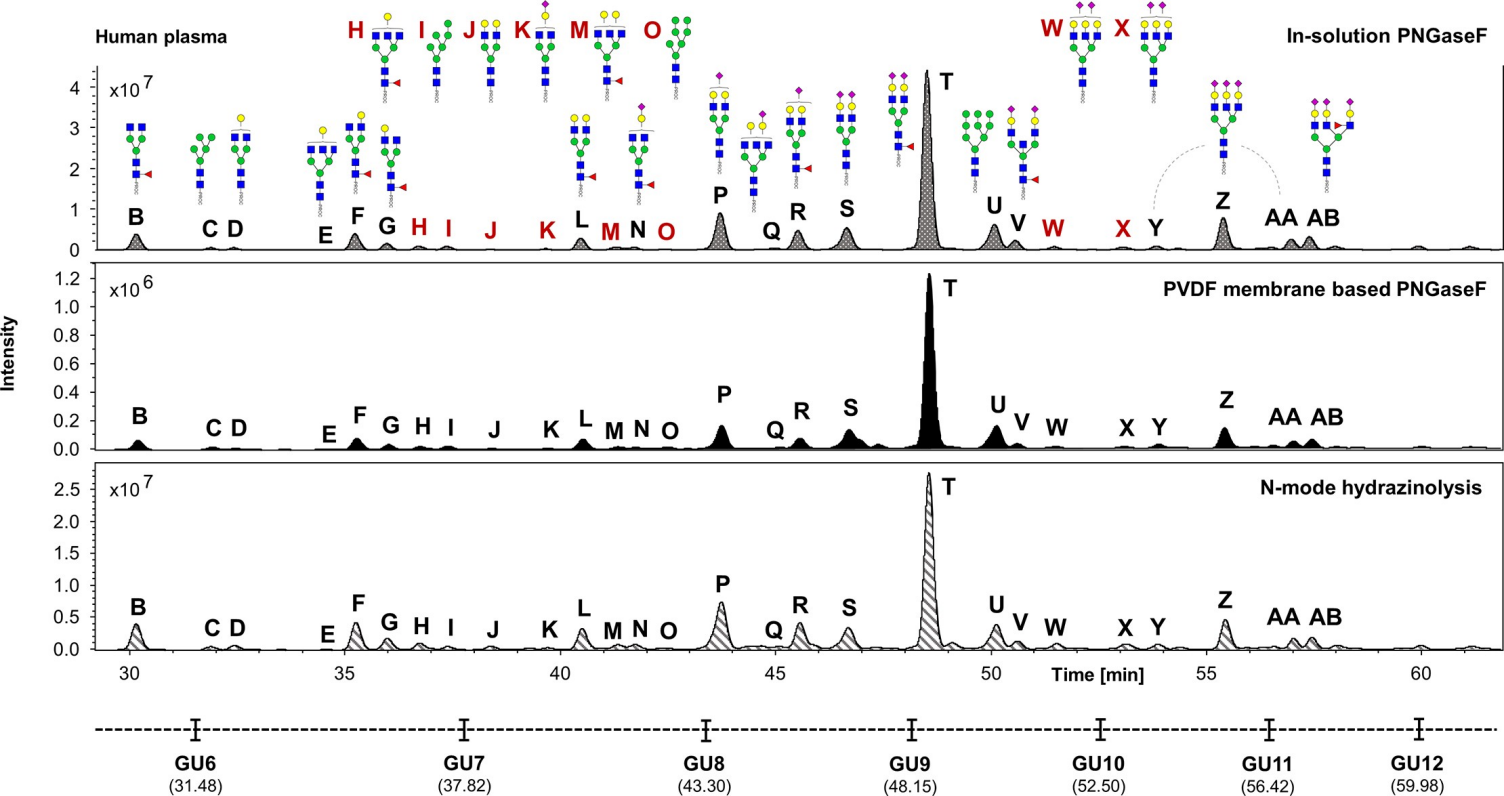
Comparison of the FLR chromatograms for human plasma *N*-glycans released by in-solution PNGaseF, PVDF membrane-based PNGaseF and *N*-mode hydrazinolysis. *N*-glycan structures, illustrated by cartoons, were assigned by MS and MS/MS fragmentation analysis.

**Fig 4:**
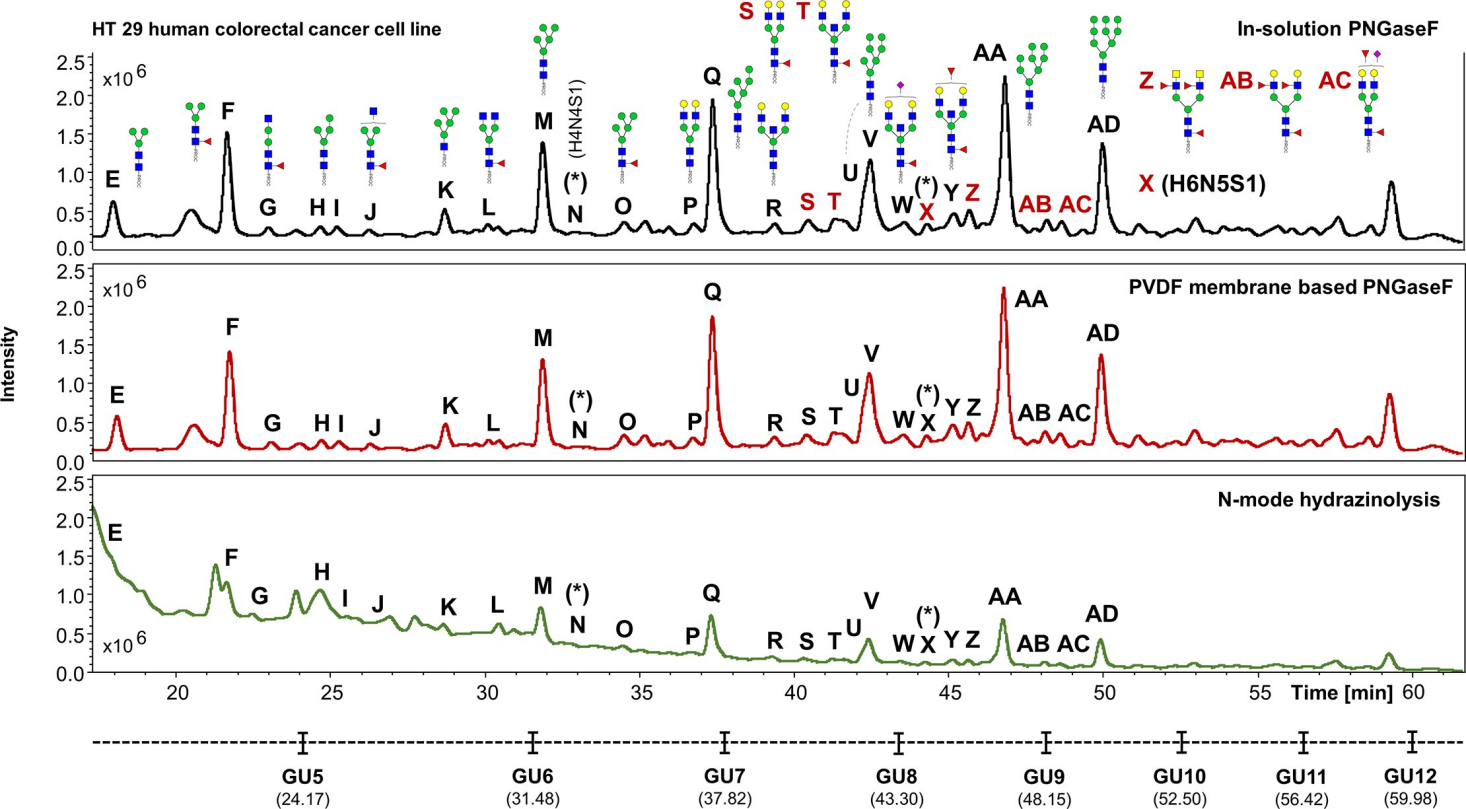
Comparison of the FLR chromatograms for HT29 human colorectal cancer cell line *N*-glycans released by in-solution PNGaseF, PVDF membrane-based PNGaseF and *N*-mode hydrazinolysis. *N*-glycan structures, illustrated by cartoons, were assigned by MS and MS/MS fragmentation analysis. (*) No MS/MS data detected for structural confirmation. Glycan compositions are given in the terms of hexose (H), *N*-acetylhexosamine (N), deoxyhexose (F), *N*-acetylneuraminic acid (S).

The UHPLC profiles obtained from the human IgG, human plasma and the HT29 cell line samples processed in experiment 1 were compared (Figs 2–4). Comparable *N*-glycan profiles of all three sample types were obtained from each approach used.

The fluorescent (FLR) chromatograms for human IgG *N*-glycans released by hydrazine showed the highest signal intensities, followed respectively by the in-solution PNGaseF and the PVDF membrane-based protocol (Fig 2).

The chromatograms for human plasma *N*-glycans released by in-solution PNGaseF showed the highest signal intensities, followed respectively by the hydrazine and the PVDF membrane-based protocol (Fig 3).

In contrast, the UHPLC data obtained from the three methods for released HT29 cell line *N*-glycans were very comparable, with the exception of few structures detected at lower retention times, which seem to show much higher signal intensities on the hydrazine protocol (Fig 4, structures E and H).

**Fig 5:**
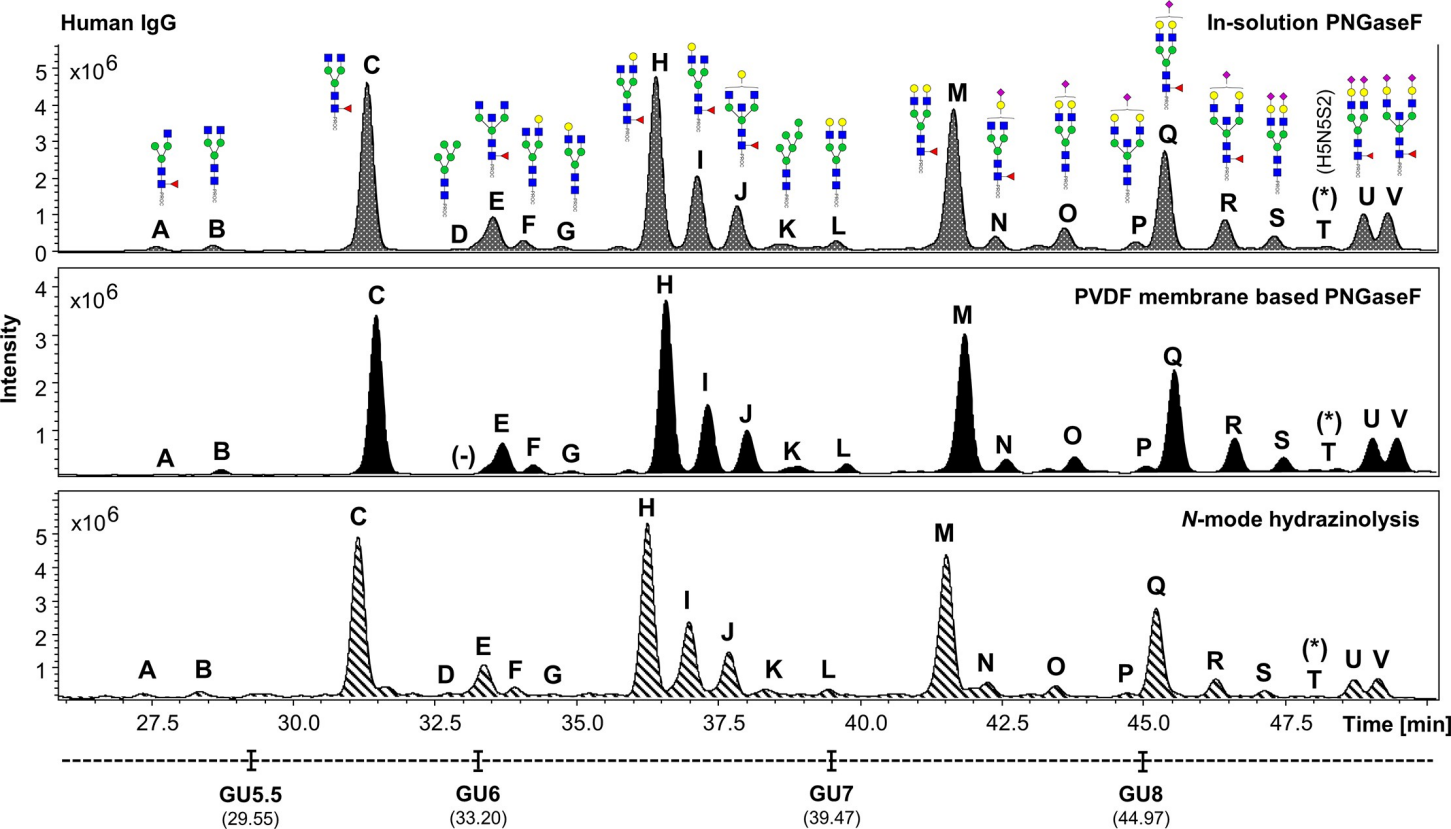
Comparison of the FLR chromatograms for human IgG *N*-glycans released by in-solution PNGaseF, PVDF membrane-based PNGaseF and N-mode hydrazinolysis. (Experiment 2). *N*-glycan structures, illustrated by cartoons, were assigned by MS and MS/MS fragmentation analysis. (-) Missing structures. (*) No MS/MS data detected for structural confirmation. Glycan compositions are given in the terms of hexose (H), *N*-acetylhexosamine (N), deoxyhexose (F), *N*-acetylneuraminic acid (S).

**Fig 6:**
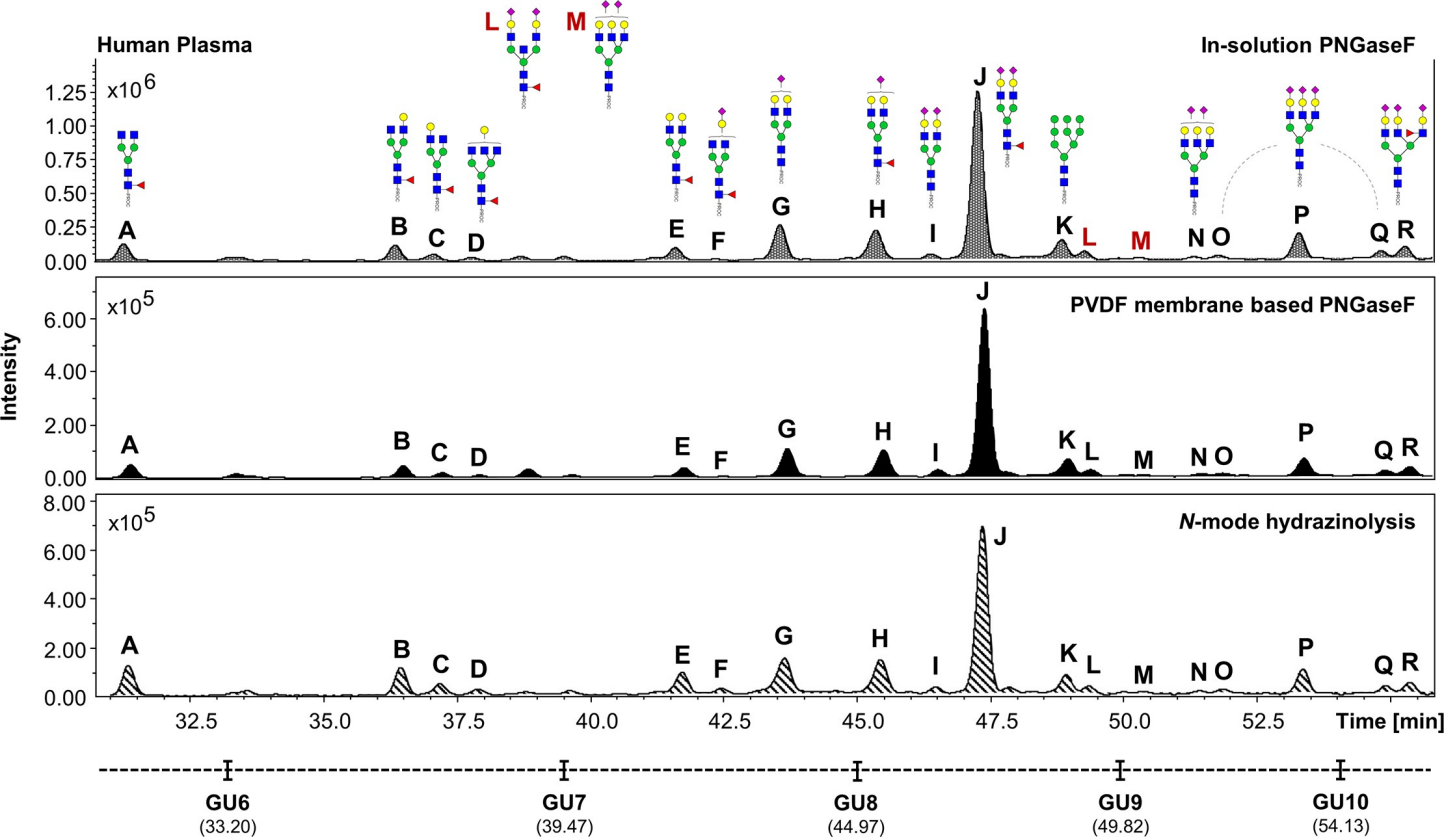
Comparison of the FLR chromatograms for human plasma *N*-glycans released by in-solution PNGaseF, PVDF membrane-based PNGaseF and N-mode hydrazinolysis. (Experiment 2). *N*-glycan structures, illustrated by cartoons, were assigned by MS and MS/MS fragmentation analysis.

With regards to signal intensities generated in experiment 2, the FLR chromatograms for hydrazine and in-solution PNGaseF released human IgG *N*-glycans showed similar signal intensities. Both methods were able to outperform the PVDF membrane-based PNGaseF protocol (Fig 5). A similar outcome to the one observed in experiment 1 was observed in the chromatograms for human plasma released *N*-glycans, where the in-solution PNGaseF method showed the highest signal intensities generated, followed respectively by the hydrazine and the PVDF membrane-based protocols (Fig 6).

**Fig 7:**
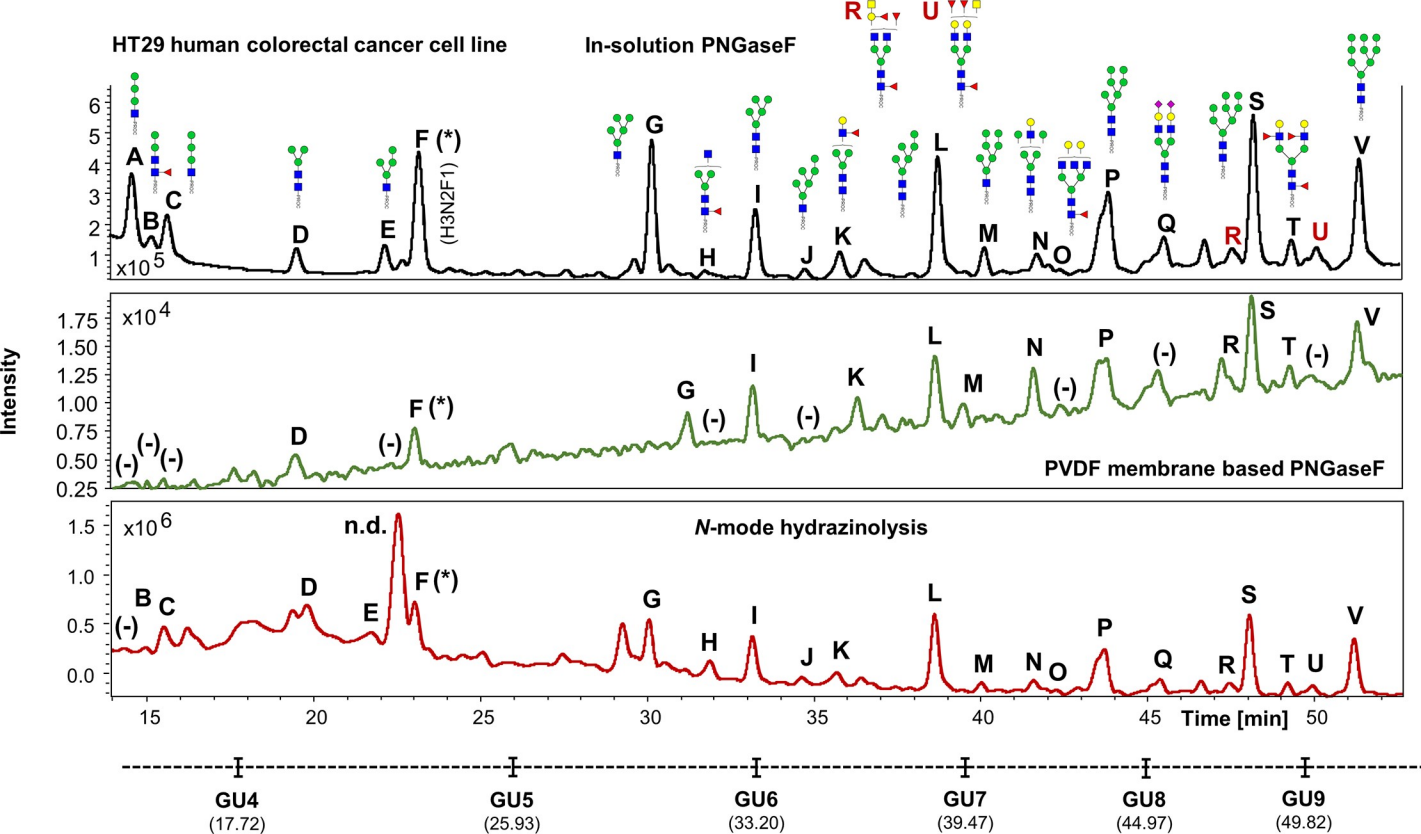
Comparison of the FLR chromatograms for HT29 human colorectal cancer cell line *N*-glycans released by in-solution PNGaseF, PVDF membrane-based PNGaseF and N-mode hydrazinolysis. (Experiment 2). *N*-glycan structures, illustrated by cartoons, were assigned by MS and MS/MS fragmentation analysis. (-) Missing structures. (n.d.) Artefacts or contaminants. (*) No MS/MS data detected for structural confirmation. Glycan compositions are given in the terms of hexose (H), *N*-acetylhexosamine (N), deoxyhexose (F), *N*-acetylneuraminic acid (S).

**Fig 8:**
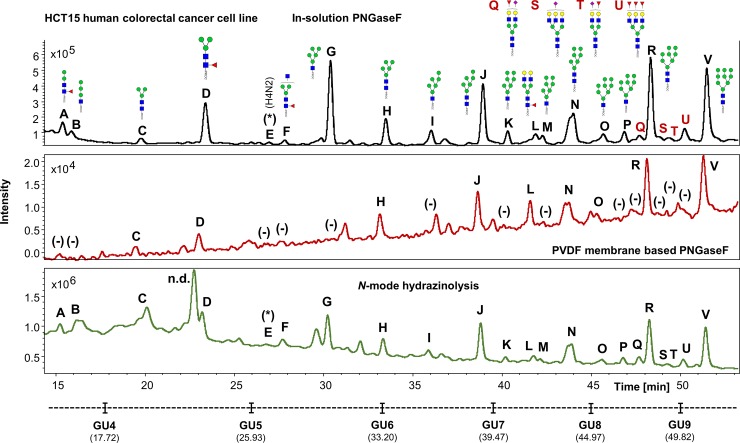
Comparison of the FLR chromatograms for HCT15 human colorectal cancer cell line *N*-glycans released by in-solution PNGaseF, PVDF membrane-based PNGaseF and N-mode hydrazinolysis. (Experiment 2). *N*-glycan structures, illustrated by cartoons, were assigned by MS and MS/MS fragmentation analysis. (-) Missing structures. (n.d.) Artefacts or contaminants. (*) No MS/MS data detected for structural confirmation. Glycan compositions are given in the terms of hexose (H), *N*-acetylhexosamine (N), deoxyhexose (F), *N*-acetylneuraminic acid (S).

**Fig 9:**
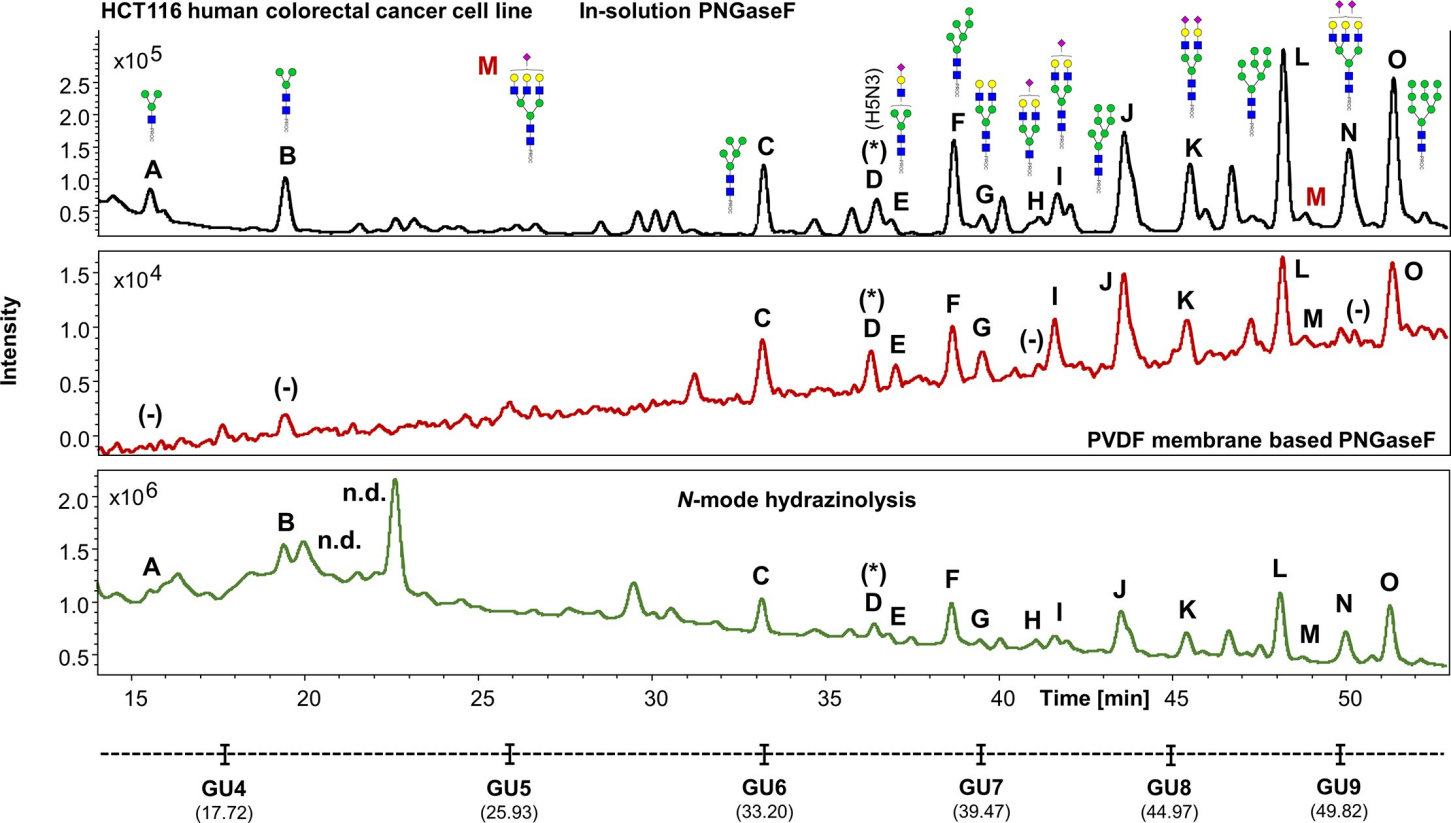
Comparison of the FLR chromatograms for HCT116 human colorectal cancer cell line *N*-glycans released by in-solution PNGaseF, PVDF membrane-based PNGaseF and N-mode hydrazinolysis. (Experiment 2). *N*-glycan structures, illustrated by cartoons, were assigned by MS and MS/MS fragmentation analysis. (-) Missing structures. (n.d.) Artefacts or contaminants. (*) No MS/MS data detected for structural confirmation. Glycan compositions are given in the terms of hexose (H), *N*-acetylhexosamine (N), deoxyhexose (F), *N*-acetylneuraminic acid (S).

**Fig 10:**
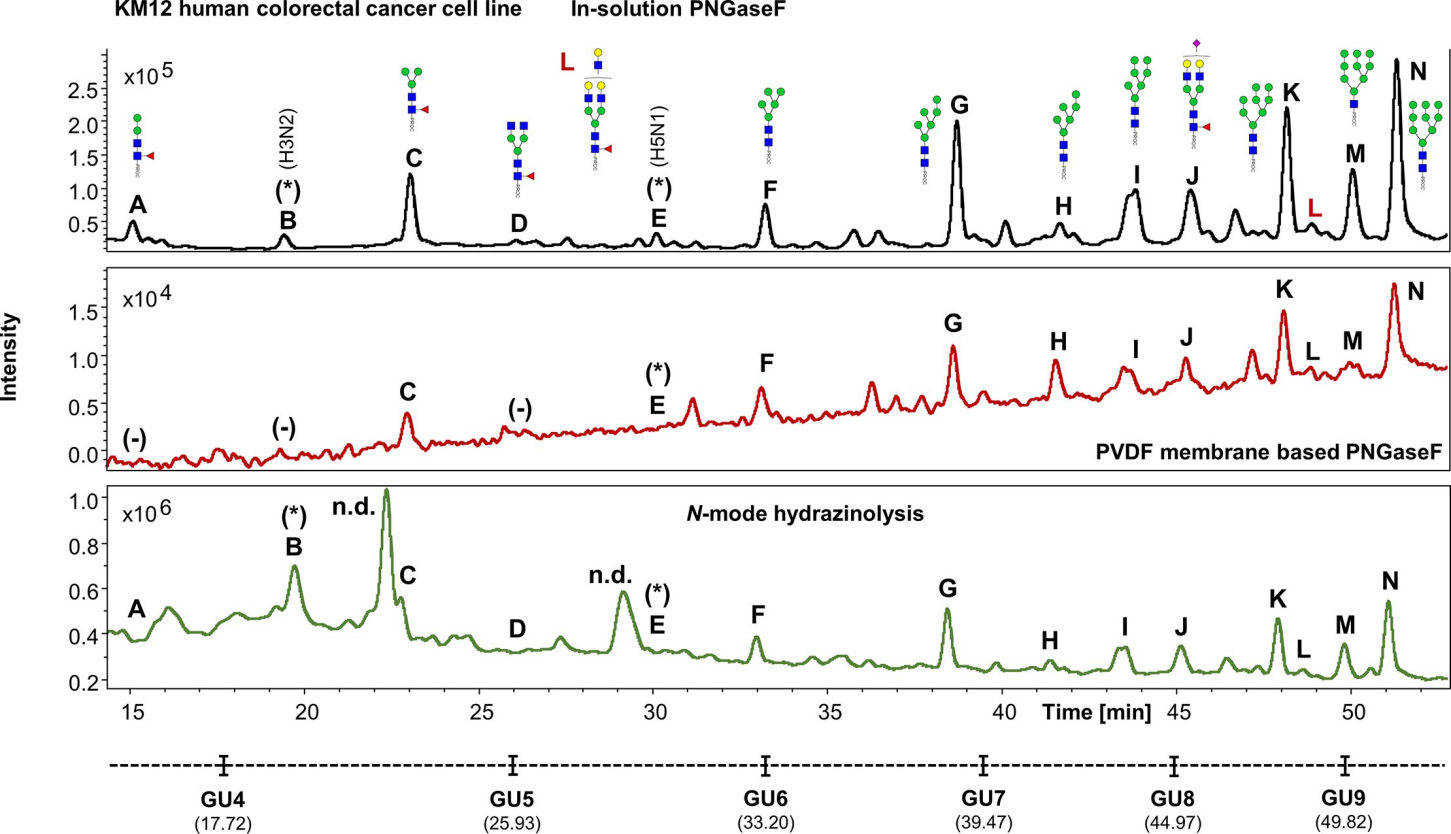
Comparison of the FLR chromatograms for KM12 human colorectal cancer cell line *N*-glycans released by in-solution PNGaseF, PVDF membrane-based PNGaseF and N-mode hydrazinolysis. (Experiment 2). *N*-glycan structures, illustrated by cartoons, were assigned by MS and MS/MS fragmentation analysis. (-) Missing structures. (n.d.) Artefacts or contaminants. (*) No MS/MS data detected for structural confirmation. Glycan compositions are given in the terms of hexose (H), *N*-acetylhexosamine (N), deoxyhexose (F), *N*-acetylneuraminic acid (S).

The UHPLC profiles from human IgG, human plasma and the HT29, HCT15, HCT116 and KM12 cell line samples were mostly comparable with the exception of few extra peaks only detected in cell lines when the hydrazine protocol is used (i.e. Figs 7–10, peaks labelled as “n.d.”). No *N*-glycan structures were identified from the MS/MS fragmentation analysis performed on these peaks, suggesting that the FLR signal detected may originate from contaminants present in the cell media and/or produced during sample preparation, coupled to the strong chemical reagents used in this method. This could be a limitation of the methodology, where the UHPLC profiles obtained, especially at lower retention times (0–32 min), are harder to interpret (Figs 4, 7, 8, 9 and 10), thus reflecting in the poor reproducibility of the data generated for *N*-glycans eluting at these retention times and consequently limiting, at a certain degree, the relative and/or absolute quantitation ability of the method.

The FLR signal scale used to illustrate the chromatograms, coupled to the inability of the PVDF membrane-based PNGaseF protocol to provide identification for few *N*-glycan structures due to the partial loss of material not efficiently eluting through the membrane reflects on the variability of few portions of the UHPLC profiles showed.

In general, the in-solution PNGaseF protocol was able to visibly and consistently produce higher signal intensities for all human colorectal cancer cell lines.

For further information regarding the UHPLC profiles obtained for each replicate processed with the three protocols used in this study, refer to Figs A-X in S1 File. We must point out that peaks observed in the UHPLC profiles obtained for each method within the 0–10 min retention time window, originate from free procainamide label dye and/or contaminants present in the samples. The 0–90 min retention time window FLR chromatograms for PVDF membrane-based PNGaseF released *N*-glycans from each replicate of HCT15, HCT116, and KM12 human colorectal cancer cell lines are not shown as the intensity scale used for these illustrations, coupled to the low FLR signal intensities produced by this protocol, prevents the visual appreciation of the FLR peaks detected in these samples. (Fig R in S1 File).

Details regarding the identification and the MS/MS fragmentation analysis performed for the most abundant glycans detected in all samples are shown in Tables C-I in S1 File. With regards to *N*-glycan identification, we must point out that unlike the in-solution PNGaseF and the *N*-mode hydrazinolysis protocols, the PVDF membrane-based PNGaseF method presented limitations to this purpose, not being able to detect few *N*-glycan structures in human IgG, HT29, HCT15, HCT116 and KM12 human colorectal cancer cell lines. (Tables J-R in S1 File).

Three examples of structural assignment by MS/MS fragmentation analysis are shown in Figs Y-AA in S1 File. We must point out that despite the HT29 human colorectal cancer cell line was used in both experiments, a batch from a new cell culture was used in experiment 2. As a consequence, the glycosylation patterns observed may vary. Although all cancer cell lines processed in this study originated from a different cell culture batch, and understanding the differences in the glycosylation patterns that this may imply, similar N-glycosylation traits to previously published literature were observed in this work [29].

An overview of the CVs observed for each method for human IgG and human plasma obtained from both experiments and the HT29 (from both culture batches), HCT15, HCT116 and KM12 cell line released *N*-glycans is shown in Tables J-R in S1 File.

For all release methods, the CVs for human IgG released *N*-glycans from experiment 1 were < 24.39% for *N*-glycans with relative areas (RAs) ≥ 0.69%, indicating a good degree of reproducibility of the methods. (Table J in S1 File). Similar results were obtained from human IgG *N*-glycans released from experiment 2, with CVs < 19.89% for *N*-glycans with RAs ≥ 0.41%. (Table M in S1 File).

In contrast, while the in-solution PNGaseF and the hydrazine methods were able to exhibit reproducible results for human plasma *N*-glycans released in experiment 1, with CVs < 5.22% for *N*-glycans with RAs ≥ 1.57%, the PVDF membrane-based PNGaseF protocol presented remarkable limits, displaying CVs up to 79.87% for *N*-glycans with RAs ≥ 5.07%, with a peak at 110.81% (Peak “AC”). (Table K in S1 File). Results from human plasma *N*-glycans released in experiment 2 showed slightly different outcomes. Overall, all methods were able to exhibit reproducible results, with the in-solution PNGaseF method outperforming the PVDF membrane-based PNGaseF and the hydrazine protocols, being able to produce CVs < 10.70% for *N*-glycans with RAs ≥ 0.81%. The hydrazine and the PVDF membrane-based methods showed quite reproducible results, being able to generate respectively CVs that sporadically peaked at 13.53% for RAs ≥ 1.84 and at 27.80% for RAs ≥ 1.08%. However, when taking into consideration all CVs presented, the PVDF membrane-based PNGaseF protocol confirmed to be the less reproducible method for *N*-glycan release and analysis. (Table N in S1 File). These differences may be the direct consequence of the extra separation processes involved in the PVDF membrane-based PNGaseF protocol. This type of membrane allows good filtration rates for relatively simple samples, but can frequently become clogged when more complex types of samples, like human plasma, are applied, thus resulting in poor reproducibility of the results.

In order to facilitate the comparison of the results obtained from the analysis of *N*-glycans released from human colorectal cancer cell lines, we limited the comparison of the extracted CVs to all detected *N*-glycan structures with RAs ≥ 2%.

With regards to the analysis of *N*-glycans released from HT29 cell line in experiment 1, all release methods were able to produce similar results. The in-solution PNGaseF protocol proved to be the best strategy by exhibiting CVs < 8.67% with two peaks at 18.16% and 13.65%, followed by the PVDF membrane-based PNGaseF and hydrazine and protocols which were able to produce respectively CVs < 18.98% and 20.88%. (Table L in S1 File).

The data obtained from all cell lines processed in experiment 2 proved that the in-solution PNGaseF protocol is able to consistently produce a higher degree of repeatability of the results generated (Tables O-R in S1 File).

CVs obtained from HT29 released *N*-glycans from experiment 2 by in-solution PNGaseF were < 6.60%. The hydrazine protocol produced CVs < 8.04%, with sporadic peaks at ca. 20%, followed by the PVDF membrane-based PNGaseF protocol, with CVs < 27.60% and one sporadic peak at 97.16%. (Table O in S1 File).

CVs obtained from HCT15 released *N*-glycans by in-solution PNGaseF were < 10.31%, followed by the hydrazine protocol, being able to produce CVs < 20.20%, and by the PVDF membrane-based PNGaseF protocol, with CVs < 24.26% and one sporadic peak at 39.42%. (Table P in S1 File).

CVs obtained from HCT116 released *N*-glycans by in-solution PNGaseF were < 7.78%, with one sporadic peak at 14.95%. The hydrazine protocol produced CVs < 15.25%, followed by the PVDF membrane-based PNGaseF protocol with CVs < 28.51%. (Table Q in S1 File).

CVs obtained from KM12 released *N*-glycans by in-solution PNGaseF were < 12.20%. The hydrazine release method produced CVs which peaked at 18.67%, followed by the PVDF membrane-based PNGaseF protocol, which exhibited CVs that peaked at 39.63%. (Table R in S1 File).

Considering the recent advances in *N*-glycan glycomics in terms of throughput, one important feature that should be taken into consideration is the high-throughput (HT) potential of each approach.[35–37] The in-solution and the PVDF membrane-based PNGaseF systems show good high-throughput potential over the hardly automatable hydrazine protocol. In this context, the hazardous properties of anhydrous hydrazine and subsequent storage and usage requirements may present a number of challenges.

Taking into account all presented data, we conclude that all three approaches represent a good choice for the release, recovery, identification and characterization of the *N*-glycan species. Their choice will depend on such factors as the type of glycosylation present, the nature and amount of sample. Based on our recent results indicating faster processing times, higher sensitivity and repeatability, we demonstrate that the in-solution PNGaseF method is robust, and can be used as a standard approach for *N*-glycan profiling and identification on a broad range of sample types, being able to consistently produce better UHPLC profiles, and lower coefficients of variation.

## Supporting information

S1 File **Table A.** Glycan compositions and proposed structures, average GU values, average relative areas (average % area), standard deviations (SDs) and coefficients of variation (CVs) for the most abundant *N*-glycan structures detected in human IgG and calculated after triplicate analysis. Glycans from three independent human IgG samples were released, labelled and analysed by LC-MS in two separate days (three samples on day 1 versus three samples on day 2) to assess interday variation. Structures for *N-*glycans are depicted following the Consortium for Functional Glycomics (CFG) notation: *N*-acetylglucosamine (N; blue square), fucose (F; red triangle), galactose (H; yellow circle), mannose (H; green circle), *N*-acetylneuraminic acid (S; purple diamond). Glycan compositions are given in the terms of hexose (H), *N*-acetylhexosamine (N), deoxyhexose (F), *N*-acetylneuraminic acid (S).
**Table B.** Comparison of the execution times for in-solution PNGaseF, PVDF membrane-based PNGaseF and *N*-mode hydrazinolysis *N*-glycan release methods.
**Table C.** Structural characterization of procainamide labelled IgG *N*-glycans. Structures for *N*-glycans are depicted following the Consortium for Functional Glycomics (CFG) notation: *N*-acetylglucosamine (N; blue square), fucose (F; red triangle), galactose (H; yellow circle), mannose (H; green circle), *N*-acetylneuraminic acid (S; purple diamond). Glycan compositions are given in the terms of hexose (H), *N*-acetylhexosamine (N), deoxyhexose (F), *N*-acetylneuraminic acid (S).
**Table D.** Structural characterization of procainamide labelled plasma *N-*glycans. Structures for *N-*glycans are depicted following the Consortium for Functional Glycomics (CFG) notation: *N*-acetylglucosamine (N; blue square), fucose (F; red triangle), galactose (H; yellow circle), mannose (H; green circle), *N*-acetylneuraminic acid (S; purple diamond). Glycan compositions are given in the terms of hexose (H), *N*-acetylhexosamine (N), deoxyhexose (F), *N*-acetylneuraminic acid (S). *^,^ **No MS data detected.
**Table E.** Structural characterization of procainamide labelled HT29 human colorectal cancer cell line *N-*glycans. Structures for *N-*glycans are depicted following the Consortium for Functional Glycomics (CFG) notation: *N*-acetylglucosamine (N; blue square), fucose (F; red triangle), galactose (H; yellow circle), mannose (H; green circle), *N*-acetylneuraminic acid (S; purple diamond). Glycan compositions are given in the terms of hexose (H), *N*-acetylhexosamine (N), deoxyhexose (F), *N*-acetylneuraminic acid (S). *^,^ **^,^ ***^,^ ****No MS data detected.
**Table F.** Structural characterization of procainamide labelled HT29 human colorectal cancer cell line *N-*glycans from experiment 2. Structures for *N-*glycans are depicted following the Consortium for Functional Glycomics (CFG) notation: *N*-acetylglucosamine (N; blue square), fucose (F; red triangle), galactose (H; yellow circle), mannose (H; green circle), *N*-acetylneuraminic acid (S; purple diamond). Glycan compositions are given in the terms of hexose (H), *N*-acetylhexosamine (N), deoxyhexose (F), *N*-acetylneuraminic acid (S).
**Table G.** Structural characterization of procainamide labelled HCT15 human colorectal cancer cell line *N-*glycans from experiment 2. Structures for *N-*glycans are depicted following the Consortium for Functional Glycomics (CFG) notation: *N*-acetylglucosamine (N; blue square), fucose (F; red triangle), galactose (H; yellow circle), mannose (H; green circle), *N*-acetylneuraminic acid (S; purple diamond). Glycan compositions are given in the terms of hexose (H), *N*-acetylhexosamine (N), deoxyhexose (F), *N*-acetylneuraminic acid (S).
**Table H.** Structural characterization of procainamide labelled HCT116 human colorectal cancer cell line *N-*glycans from experiment 2. Structures for *N-*glycans are depicted following the Consortium for Functional Glycomics (CFG) notation: *N*-acetylglucosamine (N; blue square), fucose (F; red triangle), galactose (H; yellow circle), mannose (H; green circle), *N*-acetylneuraminic acid (S; purple diamond). Glycan compositions are given in the terms of hexose (H), *N*-acetylhexosamine (N), deoxyhexose (F), *N*-acetylneuraminic acid (S).
**Table I.** Structural characterization of procainamide labelled HCT116 human colorectal cancer cell line *N-*glycans from experiment 2. Structures for *N-*glycans are depicted following the Consortium for Functional Glycomics (CFG) notation: *N*-acetylglucosamine (N; blue square), fucose (F; red triangle), galactose (H; yellow circle), mannose (H; green circle), *N*-acetylneuraminic acid (S; purple diamond). Glycan compositions are given in the terms of hexose (H), *N*-acetylhexosamine (N), deoxyhexose (F), *N*-acetylneuraminic acid (S).
**Table J.** Glycan compositions and proposed structures, average GU values, average relative areas (average % area), standard deviations (SDs) and coefficients of variation (CVs) for the most abundant *N*-glycan structures detected in human IgG from experiment 1 and calculated after triplicate analysis. Structures for *N-*glycans are depicted following the Consortium for Functional Glycomics (CFG) notation: *N*-acetylglucosamine (N; blue square), fucose (F; red triangle), galactose (H; yellow circle), mannose (H; green circle), *N*-acetylneuraminic acid (S; purple diamond). Glycan compositions are given in the terms of hexose (H), *N*-acetylhexosamine (N), deoxyhexose (F), *N*-acetylneuraminic acid (S).
**Table K.** Glycan compositions and proposed structures, average GU values, average relative areas (average % area), standard deviations (SDs) and coefficients of variation (CVs) for the most abundant *N*-glycan structures detected in human plasma from experiment 1 and calculated after triplicate analysis. Structures for *N-*glycans are depicted following the Consortium for Functional Glycomics (CFG) notation: *N*-acetylglucosamine (N; blue square), fucose (F; red triangle), galactose (H; yellow circle), mannose (H; green circle), *N*-acetylneuraminic acid (S; purple diamond). Glycan compositions are given in the terms of hexose (H), *N*-acetylhexosamine (N), deoxyhexose (F), *N*-acetylneuraminic acid (S).
**Table L.** Glycan compositions and proposed structures, average GU values, average relative areas (average % area), standard deviations (SDs) and coefficients of variation (CVs) for the most abundant *N*-glycan structures detected in HT29 human colorectal cancer cell line from experiment 1 and calculated after triplicate analysis. Structures for *N-*glycans are depicted following the Consortium for Functional Glycomics (CFG) notation: *N*-acetylglucosamine (N; blue square), fucose (F; red triangle), galactose (H; yellow circle), mannose (H; green circle), *N*-acetylneuraminic acid (S; purple diamond). Glycan compositions are given in the terms of hexose (H), *N*-acetylhexosamine (N), deoxyhexose (F), *N*-acetylneuraminic acid (S).
**Table M.** Glycan compositions and proposed structures, average GU values, average relative areas (average % area), standard deviations (SDs) and coefficients of variation (CVs) for the most abundant *N*-glycan structures detected in human IgG from experiment 2 and calculated after triplicate analysis. Structures for *N-*glycans are depicted following the Consortium for Functional Glycomics (CFG) notation: *N*-acetylglucosamine (N; blue square), fucose (F; red triangle), galactose (H; yellow circle), mannose (H; green circle), *N*-acetylneuraminic acid (S; purple diamond). Glycan compositions are given in the terms of hexose (H), *N*-acetylhexosamine (N), deoxyhexose (F), *N*-acetylneuraminic acid (S).
**Table N.** Glycan compositions and proposed structures, average GU values, average relative areas (average % area), standard deviations (SDs) and coefficients of variation (CVs) for the most abundant *N*-glycan structures detected in human plasma from experiment 2 and calculated after triplicate analysis. Structures for *N-*glycans are depicted following the Consortium for Functional Glycomics (CFG) notation: *N*-acetylglucosamine (N; blue square), fucose (F; red triangle), galactose (H; yellow circle), mannose (H; green circle), *N*-acetylneuraminic acid (S; purple diamond). Glycan compositions are given in the terms of hexose (H), *N*-acetylhexosamine (N), deoxyhexose (F), *N*-acetylneuraminic acid (S).
**Table O.** Glycan compositions and proposed structures, average GU values, average relative areas (average % area), standard deviations (SDs) and coefficients of variation (CVs) for the most abundant *N*-glycan structures detected in HT29 human colorectal cancer cell line from experiment 2 and calculated after triplicate analysis. Structures for *N-*glycans are depicted following the Consortium for Functional Glycomics (CFG) notation: *N*-acetylglucosamine (N; blue square), fucose (F; red triangle), galactose (H; yellow circle), mannose (H; green circle), *N*-acetylneuraminic acid (S; purple diamond). Glycan compositions are given in the terms of hexose (H), *N*-acetylhexosamine (N), deoxyhexose (F), *N*-acetylneuraminic acid (S).
**Table P.** Glycan compositions and proposed structures, average GU values, average relative areas (average % area), standard deviations (SDs) and coefficients of variation (CVs) for the most abundant *N*-glycan structures detected in HCT15 human colorectal cancer cell line from experiment 2 and calculated after triplicate analysis. Structures for *N-*glycans are depicted following the Consortium for Functional Glycomics (CFG) notation: *N*-acetylglucosamine (N; blue square), fucose (F; red triangle), galactose (H; yellow circle), mannose (H; green circle), *N*-acetylneuraminic acid (S; purple diamond). Glycan compositions are given in the terms of hexose (H), *N*-acetylhexosamine (N), deoxyhexose (F), *N*-acetylneuraminic acid (S).
**Table Q.** Glycan compositions and proposed structures, average GU values, average relative areas (average % area), standard deviations (SDs) and coefficients of variation (CVs) for the most abundant *N*-glycan structures detected in HCT116 human colorectal cancer cell line from experiment 2 and calculated after triplicate analysis. Structures for *N-*glycans are depicted following the Consortium for Functional Glycomics (CFG) notation: *N*-acetylglucosamine (N; blue square), fucose (F; red triangle), galactose (H; yellow circle), mannose (H; green circle), *N*-acetylneuraminic acid (S; purple diamond). Glycan compositions are given in the terms of hexose (H), *N*-acetylhexosamine (N), deoxyhexose (F), *N*-acetylneuraminic acid (S).
**Table R.** Glycan compositions and proposed structures, average GU values, average relative areas (average % area), standard deviations (SDs) and coefficients of variation (CVs) for the most abundant *N*-glycan structures detected in KM12 human colorectal cancer cell line from experiment 2 and calculated after triplicate analysis. Structures for *N-*glycans are depicted following the Consortium for Functional Glycomics (CFG) notation: *N*-acetylglucosamine (N; blue square), fucose (F; red triangle), galactose (H; yellow circle), mannose (H; green circle), *N*-acetylneuraminic acid (S; purple diamond). Glycan compositions are given in the terms of hexose (H), *N*-acetylhexosamine (N), deoxyhexose (F), *N*-acetylneuraminic acid (S).
**Fig A.** Comparison of the FLR chromatograms for the three replicates of human IgG *N*-glycans released by in-solution PNGaseF in experiment 1.
**Fig B.** Comparison of the FLR chromatograms for the three replicates of human plasma *N*-glycans released by in-solution PNGaseF in experiment 1.
**Fig C.** Comparison of the FLR chromatograms for the three replicates of HT29 human colorectal cancer cell line *N*-glycans released by in-solution PNGaseF in experiment 1.
**Fig D.** Comparison of the FLR chromatograms for the three replicates of human IgG *N*-glycans released by PVDF membrane-based PNGaseF in experiment 1.
**Fig E.** Comparison of the FLR chromatograms for the three replicates of human plasma *N*-glycans released by PVDF membrane-based PNGaseF in experiment 1.
**Fig F.** Comparison of the FLR chromatograms for the three replicates of HT29 human colorectal cancer cell line *N*-glycans released by PVDF membrane-based PNGaseF in experiment 1.
**Fig G.** Comparison of the FLR chromatograms for the three replicates of human IgG *N*-glycans released by *N*-mode hydrazinolysis in experiment 1.
**Fig H.** Comparison of the FLR chromatograms for the three replicates of human plasma *N*-glycans released by *N*-mode hydrazinolysis in experiment 1.
**Fig I.** Comparison of the FLR chromatograms for the three replicates of HT29 human colorectal cancer cell line *N*-glycans released by *N*-mode hydrazinolysis in experiment 1.
**Fig J.** Comparison of the FLR chromatograms for the three replicates of human IgG *N*-glycans released by in-solution PNGaseF in experiment 2.
**Fig K.** Comparison of the FLR chromatograms for the three replicates of human plasma *N*-glycans released by in-solution PNGaseF in experiment 2.
**Fig L.** Comparison of the FLR chromatograms for the three replicates of HT29 human colorectal cancer cell line *N*-glycans released by in-solution PNGaseF in experiment 2.
**Fig M.** Comparison of the FLR chromatograms for the three replicates of HCT15 human colorectal cancer cell line *N*-glycans released by in-solution PNGaseF in experiment 2.
**Fig N.** Comparison of the FLR chromatograms for the three replicates of HCT116 human colorectal cancer cell line *N*-glycans released by in-solution PNGaseF in experiment 2.
**Fig O.** Comparison of the FLR chromatograms for the three replicates of KM12 human colorectal cancer cell line *N*-glycans released by in-solution PNGaseF in experiment 2.
**Fig P.** Comparison of the FLR chromatograms for the three replicates of human IgG *N*-glycans released by PVDF membrane-based PNGaseF in experiment 2.
**Fig Q.** Comparison of the FLR chromatograms for the three replicates of human plasma *N*-glycans released by PVDF membrane-based PNGaseF in experiment 2.
**Fig R.** Comparison of the FLR chromatograms for the three replicates of HT29 human colorectal cancer cell line *N*-glycans released by PVDF membrane-based PNGaseF in experiment 2.
**Fig S.** Comparison of the FLR chromatograms for the three replicates of human IgG *N*-glycans released by *N*-mode hydrazinolysis in experiment 2.
**Fig T.** Comparison of the FLR chromatograms for the three replicates of human plasma *N*-glycans released by *N*-mode hydrazinolysis in experiment 2.
**Fig U.** Comparison of the FLR chromatograms for the three replicates of HT29 human colorectal cancer cell line *N*-glycans released by *N*-mode hydrazinolysis in experiment 2.
**Fig V.** Comparison of the FLR chromatograms for the three replicates of HCT15 human colorectal cancer cell line *N*-glycans released by *N*-mode hydrazinolysis in experiment 2.
**Fig W.** Comparison of the FLR chromatograms for the three replicates of HCT116 human colorectal cancer cell line *N*-glycans released by *N*-mode hydrazinolysis in experiment 2.
**Fig X.** Comparison of the FLR chromatograms for the three replicates of KM12 human colorectal cancer cell line *N*-glycans released by *N*-mode hydrazinolysis in experiment 2.
**Fig Y.** Fragment ion spectra of precursor *m/z* 970.99, [M + PROC]^2+^ from HT29 human colorectal cancer cell line *N*-glycans released by in-solution PNGaseF.
**Fig Z.** Fragment ion spectra of precursor *m/z* 1276.57, [M + PROC]^+^ from HCT15 human colorectal cancer cell line *N*-glycans released by in-solution PNGaseF.
**Fig AA.** Fragment ion spectra of precursor *m/z* 814.99, [M + PROC]^+^ from HCT116 human colorectal cancer cell line *N*-glycans released by in-solution PNGaseF.
(DOCX)

## References

[1] Schwarz F, Aebi M. Mechanisms and principles of N-linked protein glycosylation. Curr Opin Struct Biol [Internet]. Elsevier Ltd; 2011;21(5):576–82. Available from: 10.1016/j.sbi.2011.08.005 21978957

[2] Singh S, Darbari H, Bhattacharjee K, Verma S. Open source NLG systems: A survey with a vision to design a true NLG system. Int J Control Theory Appl. 2016;9(10):4409–21.

[3] Salimonu LS, Johnson AOK, Williams AIO, Iyabo Adeleye G, Osunkoya BO. Phagocyte function in protein-calorie malnutrition. Nutr Res. 1982;2(4):445–54.

[4] Han BW, Kim WS, Lee JK, Lee SY, Park SH, Kim YI, et al Angular dependency of magnetization losses in continuously transposed coated conductors for large current applications. Trans Korean Inst Electr Eng. 2010;59(1):51–6.

[5] Rudd PM, Elliott T, Cresswell P, Wilson IA, Dwek RA. Glycosylation and the immune system. Science (80-). 2001;291(5512):2370–6. 11269318 10.1126/science.291.5512.2370

[6] Tanichi N, Korekane H. Branched N-glycans and their implications for cell adhesion, signaling and clinical applications for cancer biomarkers and in therapeutics. BMB Rep. 2011;44(12):772–81. 10.5483/bmbrep.2011.44.12.772 22189679

[7] Molinari M. N-glycan structure dictates extension of protein folding or onset of disposal. Nat Chem Biol. 2007;3(6):313–20. 10.1038/nchembio880 17510649

[8] Clerc F, Reiding KR, Jansen BC, Kammeijer GSM, Bondt A, Wuhrer M. Human plasma protein N-glycosylation. Glycoconj J. 2016;33(3):309–43. 10.1007/s10719-015-9626-2 26555091 PMC4891372

[9] Lasebikan VO, Adebayo S. Need for Screening for Alcohol and Drugs in Emergency Trauma Units. East Afr Med J [Internet]. Elsevier B.V.; 2013;90(5):164–70. Available from: 10.1016/j.bbagen.2011.12.001 26859007

[10] Freeze HH. Genetic defects in the human glycome. Nat Rev Genet. 2006;7(7):537–51. 10.1038/nrg1894 16755287

[11] Jaeken J, Matthijs G. Congenital Disorders of Glycosylation: A Rapidly Expanding Disease Family. Annu Rev Genomics Hum Genet [Internet]. 2007;8(1):261–78. Available from: http://www.annualreviews.org/doi/10.1146/annurev.genom.8.080706.092327 17506657 10.1146/annurev.genom.8.080706.092327

[12] Jeff G, Schnapp BJ, Sheetz MP. 198 8 Nature Publishing Group. Nature. 1988;331:450 10.1038/331450a0 3123999

[13] Audfray A, Varrot A, Imberty A. Bacteria love our sugars: Interaction between soluble lectins and human fucosylated glycans, structures, thermodynamics and design of competing glycocompounds. Comptes Rendus Chim [Internet]. Academie des sciences; 2013;16(5):482–90. Available from: 10.1016/j.crci.2012.11.021

[14] Imberty A, Varrot A. Microbial recognition of human cell surface glycoconjugates. Curr Opin Struct Biol. 2008;18(5):567–76. 10.1016/j.sbi.2008.08.001 18809496

[15] Dennis JW, Nabi IR, Demetriou M. Organization, Cell Surface and disease. Cell. 2009;139(7):1229–41. 10.1016/j.cell.2009.12.008 20064370 PMC3065826

[16] Takahashi M, Kizuka Y, Ohtsubo K, Gu J, Taniguchi N. Disease-associated glycans on cell surface proteins. Mol Aspects Med [Internet]. Elsevier Ltd; 2016;51:56–70. Available from: 10.1016/j.mam.2016.04.008 27131428

[17] Taniguchi N, Kizuka Y. Glycans and cancer: Role of N-Glycans in cancer biomarker, progression and metastasis, and therapeutics [Internet]. 1st ed. Vol. 126, Advances in Cancer Research. Elsevier Inc.; 2015 11–51 p. Available from: 10.1016/bs.acr.2014.11.001 25727145

[18] Kizuka Y, Kitazume S, Taniguchi N. N-glycan and Alzheimer’s disease. Biochim Biophys Acta—Gen Subj [Internet]. Elsevier B.V.; 2017;1861(10):2447–54. Available from: 10.1016/j.bbagen.2017.04.012 28465241

[19] Higel F, Seidl A, Sörgel F, Friess W. N-glycosylation heterogeneity and the influence on structure, function and pharmacokinetics of monoclonal antibodies and Fc fusion proteins. Eur J Pharm Biopharm [Internet]. Elsevier B.V.; 2016;100(January):94–100. Available from: 10.1016/j.ejpb.2016.01.005 26775146

[20] Zhang P, Woen S, Wang T, Liau B, Zhao S, Chen C, et al Challenges of glycosylation analysis and control: An integrated approach to producing optimal and consistent therapeutic drugs. Drug Discov Today [Internet]. Elsevier Ltd; 2016;21(5):740–65. Available from: 10.1016/j.drudis.2016.01.006 26821133

[21] Griebenow KAI, Sola RJ. Effects of Glycosylation on the Stability of Protein Pharmaceuticals. J Pharm Sci. 2009;98(4):1223–45. 10.1002/jps.21504 18661536 PMC2649977

[22] Mariño K, Bones J, Kattla JJ, Rudd PM. A systematic approach to protein glycosylation analysis: A path through the maze. Nat Chem Biol [Internet]. Nature Publishing Group; 2010;6(10):713–23. Available from: 10.1038/nchembio.437 20852609

[23] Balog CIA, Stavenhagen K, Fung WLJ, Koeleman CA, McDonnell LA, Verhoeven A, et al *N* -glycosylation of Colorectal Cancer Tissues. Mol Cell Proteomics [Internet]. 2012;11(9):571–85. Available from: 10.1074/mcp.M111.011601 22573871 PMC3434767

[24] Cornelissen LAM, Van Vliet SJ. A bitter sweet symphony: Immune responses to altered o-glycan epitopes in cancer. Biomolecules. 2016;6(2):1–19. 27153100 10.3390/biom6020026 PMC4919921

[25] Chik JHL, Zhou J, Moh ESX, Christopherson R, Clarke SJ, Molloy MP, et al Comprehensive glycomics comparison between colon cancer cell cultures and tumours: Implications for biomarker studies. J Proteomics [Internet]. Elsevier B.V.; 2014;108:146–62. Available from: 10.1016/j.jprot.2014.05.002 24840470

[26] Merry T, Astrautsova S. Chemical and Enzymatic Release of Glycans from Glycoproteins. Capill Electrophor Carbohydrates [Internet]. 213:27–40. Available from: http://link.springer.com/10.1385/1-59259-294-5:27 10.1385/1-59259-294-5:27 12619983

[27] Sun G, Yu X, Bao C, Wang L, Li M, Gan J, et al Identification and characterization of a novel prokaryotic peptide: N-Glycosidase from Elizabethkingia meningoseptica. J Biol Chem. 2015;290(12):7452–62. 10.1074/jbc.M114.605493 25614628 PMC4367255

[28] Burnina I, Hoyt E, Lynaugh H, Li H, Gong B. A cost-effective plate-based sample preparation for antibody N-glycan analysis. J Chromatogr A [Internet]. Elsevier B.V.; 2013;1307:201–6. Available from: 10.1016/j.chroma.2013.07.104 23932029

[29] Holst S, Deuss AJM, van Pelt GW, van Vliet SJ, Garcia-Vallejo JJ, Koeleman CAM, et al N-glycosylation Profiling of Colorectal Cancer Cell Lines Reveals Association of Fucosylation with Differentiation and Caudal Type Homebox 1 (CDX1)/Villin mRNA Expression. Mol Cell Proteomics [Internet]. 2016;15(1):124–40. Available from: 10.1074/mcp.M115.051235 26537799 PMC4762531

[30] Jensen PH, Karlsson NG, Kolarich D, Packer NH. Structural analysis of N- and O-glycans released from glycoproteins. Nat Protoc [Internet]. Nature Publishing Group; 2012;7(7):1299–310. Available from: 10.1038/nprot.2012.063 22678433

[31] Jiménez-castells C, Vanbeselaere J, Kohlhuber S. Europe PMC Funders Group Gender and developmental specific N-glycomes of the porcine parasite Oesophagostomum dentatum. 2018;1861(2):418–30. 10.1016/j.bbagen.2016.10.011 PMC5201199 27751954

[32] Inka B, Royle L, Radcliffe CM, Dwek RA, Rudd PM. Detailed Structural Analysis of N-Glycans Released From Glycoproteins in SDS-PAGE Gel Bands Using HPLC Combined With Exoglycosidase Array Digestions. Glycobiol Protoc. 2006;347:125–44. 10.1385/1-59745-167-3:125 17072008

[33] Ceroni A, Maass K, Geyer H, Geyer R, Dell A, Haslam SM. GlycoWorkbench: A tool for the computer-assisted annotation of mass spectra of glycans. J Proteome Res. 2008;7(4):1650–9. 10.1021/pr7008252 18311910

[34] Varki A, Cummings RD, Aebi M, Packer NH, Seeberger PH, Esko JD, et al Symbol nomenclature for graphical representations of glycans. Glycobiology. 2015;25(12):1323–4. 10.1093/glycob/cwv091 26543186 PMC4643639

[35] Shubhakar A, Reiding KR, Gardner RA, Spencer DIR, Fernandes DL, Wuhrer M. High-Throughput Analysis and Automation for Glycomics Studies. Chromatographia. 2014;78(5–6):321–33. 10.1007/s10337-014-2803-9 25814696 PMC4363487

[36] Ventham NT, Gardner RA, Kennedy NA, Shubhakar A, Kalla R, Nimmo ER, et al Changes to serum sample tube and processing methodology does not cause inter-individual variation in automated whole serum N-Glycan profiling in health and disease. PLoS One. 2015;10(4):1–16. 10.1371/journal.pone.0123028 PMC4382121 25831126

[37] Stöckmann H, O’Flaherty R, Adamczyk B, Saldova R, Rudd PM. Automated, high-throughput serum glycoprofiling platform. Integr Biol (United Kingdom). 2015;7(9):1026–32. 10.1039/c5ib00130g 26189827

