# Supplementary material for: Method comparison for N-glycan profiling: Towards the standardization of glycoanalytical technologies for cell line analysis
Source: PLoS One. 2019 Oct 7;14(10):e0223270. doi: 10.1371/journal.pone.0223270 (PMC6779296; doi:10.1371/journal.pone.0223270)
Supplement: S1 File — Table A. Glycan compositions and proposed structures, average GU values, average relative areas (average % area), standard deviations (SDs) and coefficients of variation (CVs) for the most abundant N-glycan structures detected in human IgG and calculated after triplicate analysis. Glycans from three independent human IgG samples were released, labelled and analysed by LC-MS in two separate days (three samples on day 1 versus three samples on day 2) to assess interday variation. Structures for N-glycans are depicted following the Consortium for Functional Glycomics (CFG) notation: N-acetylglucosamine (N; blue square), fucose (F; red triangle), galactose (H; yellow circle), mannose (H; green circle), N-acetylneuraminic acid (S; purple diamond). Glycan compositions are given in the terms of hexose (H), N-acetylhexosamine (N), deoxyhexose (F), N-acetylneuraminic acid (S). Table B. Comparison of the execution times for in-solution PNGaseF, PVDF membrane-based PNGaseF and N-mode hydrazinolysis N-glycan release methods. Table C. Structural characterization of procainamide labelled IgG N-glycans. Structures for N-glycans are depicted following the Consortium for Functional Glycomics (CFG) notation: N-acetylglucosamine (N; blue square), fucose (F; red triangle), galactose (H; yellow circle), mannose (H; green circle), N-acetylneuraminic acid (S; purple diamond). Glycan compositions are given in the terms of hexose (H), N-acetylhexosamine (N), deoxyhexose (F), N-acetylneuraminic acid (S). Table D. Structural characterization of procainamide labelled plasma N-glycans. Structures for N-glycans are depicted following the Consortium for Functional Glycomics (CFG) notation: N-acetylglucosamine (N; blue square), fucose (F; red triangle), galactose (H; yellow circle), mannose (H; green circle), N-acetylneuraminic acid (S; purple diamond). Glycan compositions are given in the terms of hexose (H), N-acetylhexosamine (N), deoxyhexose (F), N-acetylneuraminic acid (S). *, **No MS data de [file pone.0223270.s001.docx]

Method comparison for *N*-glycan profiling: Towards the standardization of glycoanalytical technologies for cell line analysis.

Maximilianos Kotsias^1*^, Athanasios Blanas^2^, Sandra J. van Vliet^2^, Martina Pirro^3^, Daniel I. R. Spencer^1^, Radoslaw P. Kozak^1^

^1^Ludger Ltd., Culham Science Centre, Abingdon, Oxfordshire, UK.

^2^Amsterdam UMC, Vrije Unitersiteit Amsterdam, Molecular Cell Biology and Immunology, Cancer Center Amsterdam, Amsterdam, The Netherlands.

^3^Leiden University Medical Centre, Centre for Proteomics and Metabolomics, Leiden, The Netherlands.

***Corresponding author**

E-mail: [maximilianos.kotsias@ludger.com](mailto:maximilianos.kotsias@ludger.com) (MK)

Supporting Information

| Peak ID | Possible  structure | Composition | | | | Avg. GU |  | IgG samples released with:  *(3 samples processed on day 1 vs 3 samples processed on day 2)* | | |
| --- | --- | --- | --- | --- | --- | --- | --- | --- | --- | --- |
|  |  |  |  |  |  |  |  | IS *(n=6)* | PVDF *(n=6)* | N-Hy *(n=6)* |
|  |  | Hex | HexNAc | Fuc | Neu5Ac |  |  |  |  |  |
| C | 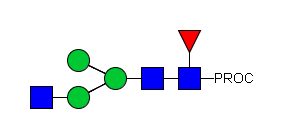 | 3 | 3 | 1 | 0 | 5.79 | Average % area | 17.15 | 17.50 | 17.36 |
|  |  |  |  |  |  |  | Standard deviation (SD) | 0.41 | 0.29 | 0.34 |
|  |  |  |  |  |  |  | Coefficient of variation (CV) | **2.38** | **1.68** | **1.97** |
| C | 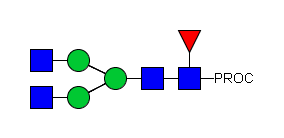 | 3 | 4 | 1 | 0 | 5.79 |  |  |  |  |
| E | 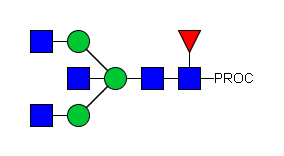 | 3 | 5 | 1 | 0 | 6.12 | Average % area | 3.75 | 3.64 | 4.10 |
|  |  |  |  |  |  |  | Standard deviation (SD) | 0.42 | 0.27 | 0.45 |
|  |  |  |  |  |  |  | Coefficient of variation (CV) | **11.12** | **7.40** | **10.89** |
| F | 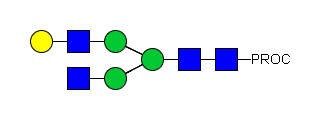 | 4 | 4 | 0 | 0 | 6.19 | Average % area | 1.34 | 0.96 | 1.32 |
|  |  |  |  |  |  |  | Standard deviation (SD) | 0.10 | 0.05 | 0.12 |
|  |  |  |  |  |  |  | Coefficient of variation (CV) | **7.23** | **5.13** | **8.77** |
| H | 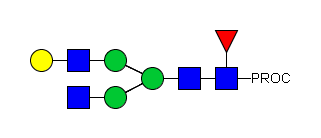 | 4 | 4 | 1 | 0 | 6.56 | Average % area | 17.19 | 18.69 | 18.28 |
|  |  |  |  |  |  |  | Standard deviation (SD) | 0.40 | 0.40 | 0.49 |
|  |  |  |  |  |  |  | Coefficient of variation (CV) | **2.30** | **2.14** | **2.68** |
| I | 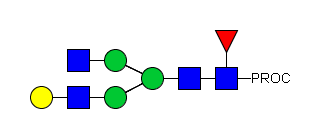 | 4 | 4 | 1 | 0 | 6.68 | Average % area | 7.87 | 7.57 | 9.42 |
|  |  |  |  |  |  |  | Standard deviation (SD) | 0.27 | 0.21 | 0.51 |
|  |  |  |  |  |  |  | Coefficient of variation (CV) | **3.40** | **2.80** | **5.44** |
| J | 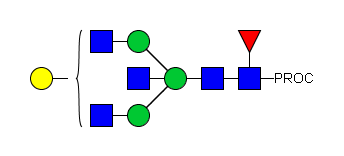 | 4 | 5 | 1 | 0 | 6.80 | Average % area | 4.67 | 4.81 | 5.57 |
|  |  |  |  |  |  |  | Standard deviation (SD) | 0.08 | 0.30 | 0.22 |
|  |  |  |  |  |  |  | Coefficient of variation (CV) | **1.67** | **6.24** | **3.88** |
| K | 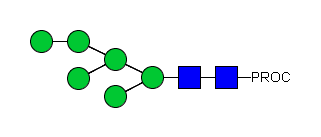 | 6 | 2 | 0 | 0 | 6.91 | Average % area | 1.35 | 1.25 | 1.61 |
|  |  |  |  |  |  |  | Standard deviation (SD) | 0.09 | 0.11 | 0.16 |
|  |  |  |  |  |  |  | Coefficient of variation (CV) | **6.88** | **9.08** | **10.04** |
| L | 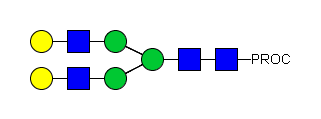 | 5 | 4 | 0 | 0 | 7.08 | Average % area | 1.10 | 0.91 | 1.13 |
|  |  |  |  |  |  |  | Standard deviation (SD) | 0.14 | 0.19 | 0.10 |
|  |  |  |  |  |  |  | Coefficient of variation (CV) | **12.80** | **20.58** | **9.17** |
| M | 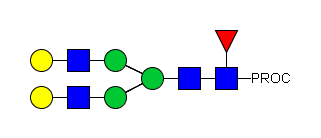 | 5 | 4 | 1 | 0 | 7.45 | Average % area | 14.26 | 15.55 | 15.68 |
|  |  |  |  |  |  |  | Standard deviation (SD) | 1.17 | 0.82 | 0.63 |
|  |  |  |  |  |  |  | Coefficient of variation (CV) | **8.22** | **5.29** | **4.04** |
| N | 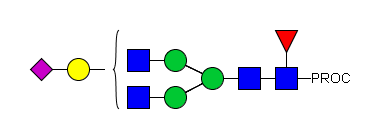 | 4 | 4 | 1 | 1 | 7.68 | Average % area | 2.14 | 1.80 | 2.37 |
|  |  |  |  |  |  |  | Standard deviation (SD) | 0.56 | 0.36 | 0.35 |
|  |  |  |  |  |  |  | Coefficient of variation (CV) | **26.32** | **20.19** | **14.64** |
| O | 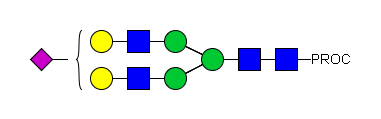 | 4 | 4 | 0 | 1 | 8.06 | Average % area | 2.37 | 1.98 | 1.47 |
|  |  |  |  |  |  |  | Standard deviation (SD) | 0.23 | 0.19 | 0.10 |
|  |  |  |  |  |  |  | Coefficient of variation (CV) | **9.84** | **9.55** | **6.92** |
| Q | 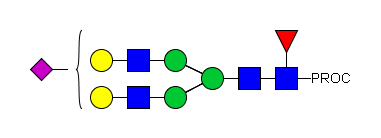 | 5 | 4 | 1 | 1 | 8.42 | Average % area | 9.99 | 10.63 | 9.82 |
|  |  |  |  |  |  |  | Standard deviation (SD) | 0.20 | 0.39 | 0.11 |
|  |  |  |  |  |  |  | Coefficient of variation (CV) | **2.05** | **3.66** | **1.15** |
| R | 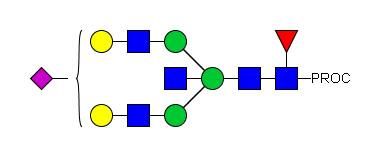 | 5 | 5 | 1 | 1 | 8.65 | Average % area | 3.47 | 3.43 | 2.36 |
|  |  |  |  |  |  |  | Standard deviation (SD) | 0.17 | 0.27 | 0.16 |
|  |  |  |  |  |  |  | Coefficient of variation (CV) | **4.79** | **7.77** | **6.79** |
| S | 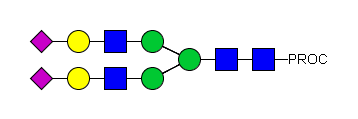 | 5 | 4 | 0 | 2 | 9.06 | Average % area | 1.53 | 1.36 | 0.88 |
|  |  |  |  |  |  |  | Standard deviation (SD) | 0.24 | 0.24 | 0.08 |
|  |  |  |  |  |  |  | Coefficient of variation (CV) | **15.94** | **17.93** | **9.32** |
| U | 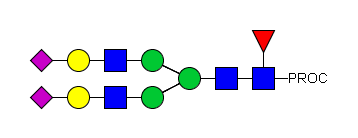 | 5 | 4 | 1 | 2 | 9.41 | Average % area | 3.71 | 3.71 | 2.10 |
|  |  |  |  |  |  |  | Standard deviation (SD) | 0.09 | 0.17 | 0.07 |
|  |  |  |  |  |  |  | Coefficient of variation (CV) | **2.36** | **4.63** | **3.36** |
| V | 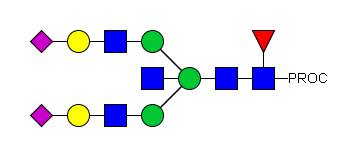 | 5 | 5 | 1 | 2 | 9.52 | Average % area | 3.64 | 3.61 | 2.13 |
|  |  |  |  |  |  |  | Standard deviation (SD) | 0.15 | 0.07 | 0.07 |
|  |  |  |  |  |  |  | Coefficient of variation (CV) | **4.24** | **1.82** | **3.30** |

**Table A.** Glycan compositions and proposed structures, average GU values, average relative areas (average % area), standard deviations (SDs) and coefficients of variation (CVs) for the most abundant *N*-glycan structures detected in human IgG and calculated after triplicate analysis. Glycans from three independent human IgG samples were released, labelled and analysed by LC-MS in two separate days (three samples on day 1 versus three samples on day 2) to assess interday variation. Structures for *N-*glycans are depicted following the Consortium for Functional Glycomics (CFG) notation: *N*-acetylglucosamine (N; blue square), fucose (F; red triangle), galactose (H; yellow circle), mannose (H; green circle), *N*-acetylneuraminic acid (S; purple diamond). Glycan compositions are given in the terms of hexose (H), *N*-acetylhexosamine (N), deoxyhexose (F), *N*-acetylneuraminic acid (S).

|  | Time (hours) | | | | |  |
| --- | --- | --- | --- | --- | --- | --- |
| Release method | **Sample preparation** | ***N*-glycan release** | ***N*-glycan conversion** | ***N*-glycan recovery** | **Labelling and clean up** | **Tot. time (hours)** |
| In-solution PNGaseF | 1.00 **^A^** | 17.16 | 1.50 | 1.50 **^A^** | 1.50 | **34.66 ^D^** |
| PVDF membrane-based PNGaseF | 1.00 **^A^** | 20.00 | 2.25 **^A^** | 1.50 **^A^** | 1.50 | **44.25 ^D^** |
| *N*-mode hydrazinolysis | 1.00 **^A^** | 5.50 **^B^** | 3.00 | 3.75 **^A^** | 1.50 | **32.75 ^C, D^** |
|  |  |  | **^A^** plus drying time | | |  |
|  |  |  | **^B^** plus drying time needed for hydrazine removal (360 min) | | |  |
|  |  |  | **^C^** Including time needed for hydrazine removal (360 min) | | |  |
|  |  |  | **^D^** Including drying time (360 min) | | |  |
|  |  |  |  | | |  |

**Table B.** Comparison of the execution times for in-solution PNGaseF, PVDF membrane-based PNGaseF and *N*-mode hydrazinolysis *N*-glycan release methods.

**IgG**

**Y**

**X**

27.5

30.0

32.5

35.0

37.5

40.0

42.5

45.0

47.5

50.0

**Time [min]**

**A**

**B**

**C**

**D**

**E**

**F**

**G**

**H**

**I**

**J**

**K**

**L**

**M**

**N**

**O**

**P**

**R**

**Q**

**S**

**T**

**U**

**V**

**W**

**Z**

| Average | | Possible structure | Composition | | | | Human IgG LC-ESI-MS (in-solution PNGaseF release) | | | | | | | | | | | |
| --- | --- | --- | --- | --- | --- | --- | --- | --- | --- | --- | --- | --- | --- | --- | --- | --- | --- | --- |
| Peak ID | GU (Procainamide) |  |  |  |  |  | [M/Z]^+^ calculated | [M/Z]^2+^ calculated | [M/Z]^3+^ calculated | [M/Z]^+^ registered | [M/Z]^2+^ registered | [M/Z]^3+^ registered | [M/Z] characteristic fragment ions (composition) | | | | | |
|  |  |  | Hex (H) | HexNAc (N) | Fuc (F) | Neu5Ac (S) |  |  |  |  |  |  |  |  |  |  |  |  |
| A | 5.28 | 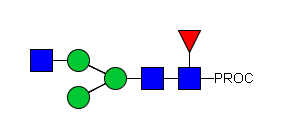 | 3 | 3 | 1 | 0 | 1479.65 | 740.33 | 493.89 | 1479.80 | 740.44 | n.d. | 441.34 (N1-PROC) | 968.46 (H2N2-PROC) | 690.25 (H3N1) |  |  |  |
|  |  |  |  |  |  |  |  |  |  |  |  |  | 587.59 (N1F1-PROC) | 1114.62 (H2N2F1-PROC) | 893.49 (H3N2) |  |  |  |
|  |  |  |  |  |  |  |  |  |  |  |  |  | 644.38 (N2-PROC) | 1276.73 (H3N2F1-PROC) |  |  |  |  |
|  |  |  |  |  |  |  |  |  |  |  |  |  | 790.57 (N2F1-PROC | 366.13 (H1N1) |  |  |  |  |
|  |  |  |  |  |  |  |  |  |  |  |  |  | 952.63 (H1N2F1-PROC) | 528.01 (H2N1) |  |  |  |  |
| B | 5.40 | 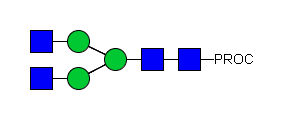 | 3 | 4 | 0 | 0 | 1536.67 | 768.84 | 512.89 | 1536.81 | 768.95 | n.d. | 441.01 (N1-PROC) | 1333.80 (H3N3-PROC) |  |  |  |  |
|  |  |  |  |  |  |  |  |  |  |  |  |  | 644.25 (N2-PROC) | 365.95 (H1N1) |  |  |  |  |
|  |  |  |  |  |  |  |  |  |  |  |  |  | 807.38 (H1N2-PROC) | 690.38 (H3N1) |  |  |  |  |
|  |  |  |  |  |  |  |  |  |  |  |  |  | 968.54 (H2N2-PROC) |  |  |  |  |  |
|  |  |  |  |  |  |  |  |  |  |  |  |  | 1131.74 (H3N2-PROC) |  |  |  |  |  |
| C | 5.79 | 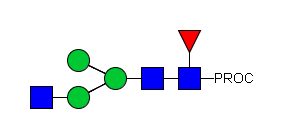 | 3 | 3 | 1 | 0 | 1479.65 | 740.33 | 493.89 | 1479.80 | n.d. | n.d. | 441.38 (N1-PROC) | 969.47 (H2N2-PROC) |  |  |  |  |
|  |  |  |  |  |  |  |  |  |  |  |  |  | 587.16 (N1F1-PROC) | 1114.50 (H2N2F1-PROC) |  |  |  |  |
|  |  |  |  |  |  |  |  |  |  |  |  |  | 644.50 (N2-PROC) | 1130.75 (H3N2-PROC) |  |  |  |  |
|  |  |  |  |  |  |  |  |  |  |  |  |  | 790.38 (N2F1-PROC) | 1276.57 (H3N2F1-PROC) |  |  |  |  |
|  |  |  |  |  |  |  |  |  |  |  |  |  | 807.50 (H1N2-PROC) | 1333.88 (H3N3-PROC) |  |  |  |  |
| C | 5.79 | 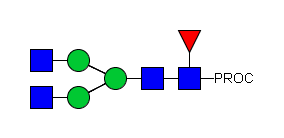 | 3 | 4 | 1 | 0 | 1682.72 | 841.87 | 561.58 | n.d. | 841.98 | n.d. | 441.34 (N1-PROC) | 968.59 (H2N2-PROC) | 1333.75 (H3N3-PROC) | 893.39 (H3N2) |  |  |
|  |  |  |  |  |  |  |  |  |  |  |  |  | 587.42 (N1F1-PROC) | 1114.72 (H2N2F1-PROC) | 1479.92 (H3N3F1-PROC) | 1096.50 (H3N3) |  |  |
|  |  |  |  |  |  |  |  |  |  |  |  |  | 644.38 (N2-PROC) | 1171.71 (H2N3-PROC) | 366.17 (H1N1) |  |  |  |
|  |  |  |  |  |  |  |  |  |  |  |  |  | 790.59 (N2F1-PROC) | 1276.78 (H3N2F1-PROC) | 528.20 (H2N1) |  |  |  |
|  |  |  |  |  |  |  |  |  |  |  |  |  | 952.63 (H1N2F1-PROC) | 1317.79 (H2N3F1-PROC) | 690.25 (H3N1) |  |  |  |
| D | 6.07 | 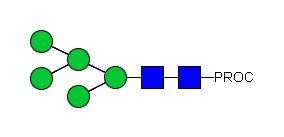 | 5 | 2 | 0 | 0 | 1454.61 | 727.81 | 485.54 | 1454.74 | 727.93 | n.d. | 441.33 (N1-PROC) | 690.25 (H3N1) |  |  |  |  |
|  |  |  |  |  |  |  |  |  |  |  |  |  | 645.25 (N2-PROC) | 893.39 (H3N2) |  |  |  |  |
|  |  |  |  |  |  |  |  |  |  |  |  |  | 807.50 (H1N2-PROC) |  |  |  |  |  |
|  |  |  |  |  |  |  |  |  |  |  |  |  | 366.12 (H1N1) |  |  |  |  |  |
|  |  |  |  |  |  |  |  |  |  |  |  |  | 528.22 (H2N1) |  |  |  |  |  |
| D | 6.07 | 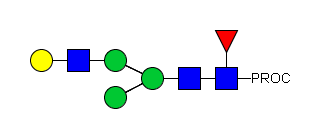 | 4 | 3 | 1 | 0 | 1641.70 | 821.35 | 547.90 | n.d. | 821.45 | n.d. | 441.31 (N1-PROC) | 1276.74 (H3N2F1-PROC) |  |  |  |  |
|  |  |  |  |  |  |  |  |  |  |  |  |  | 587.41 (N1F1-PROC) | 366.19 (H1N1) |  |  |  |  |
|  |  |  |  |  |  |  |  |  |  |  |  |  | 968.75 (H2N2-PROC) | 528.25 (H2N1) |  |  |  |  |
|  |  |  |  |  |  |  |  |  |  |  |  |  | 1114.63 (H2N2F1-PROC) | 691.63 (H3N1) |  |  |  |  |
|  |  |  |  |  |  |  |  |  |  |  |  |  | 1130.50 (H3N2-PROC) |  |  |  |  |  |
| E | 6.13 | 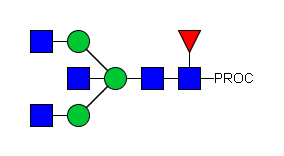 | 3 | 5 | 1 | 0 | 1885.80 | 943.41 | 629.27 | n.d. | 943.52 | 629.35 | 441.23 (N1-PROC) | 1009.63 (H1N3-PROC) | 1682.85 (H3N4F1-PROC) |  |  |  |
|  |  |  |  |  |  |  |  |  |  |  |  |  | 587.29 (N1F1-PROC) | 1171.63 (H2N3-PROC) | 366.25 (H1N1) |  |  |  |
|  |  |  |  |  |  |  |  |  |  |  |  |  | 644.50 (N2-PROC) | 1317.70 (H2N3F1-PROC) | 528.18 (H2N1) |  |  |  |
|  |  |  |  |  |  |  |  |  |  |  |  |  | 790.39 (N2F1-PROC) | 1333.64 (H3N3-PROC) | 569.13 (H1N2) |  |  |  |
|  |  |  |  |  |  |  |  |  |  |  |  |  | 806.50 (H1N2-PROC) | 1479.82 (H3N3F1-PROC) |  |  |  |  |
| F | 6.20 | 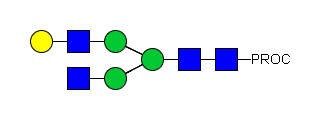 | 4 | 4 | 0 | 0 | 1698.72 | 849.86 | 566.91 | n.d. | 849.97 | n.d. | 441.25 (N1-PROC) | 1333.80 (H3N3-PROC) | 893.38 (H3N2) |  |  |  |
|  |  |  |  |  |  |  |  |  |  |  |  |  | 806.50 (H1N2-PROC) | 1495.78 (H4N3-PROC) | 1258.63 (H4N3) |  |  |  |
|  |  |  |  |  |  |  |  |  |  |  |  |  | 968.59 (H2N2-PROC) | 366.19 (H1N1) |  |  |  |  |
|  |  |  |  |  |  |  |  |  |  |  |  |  | 1130.75 (H3N2-PROC) | 528.12 (H2N1) |  |  |  |  |
|  |  |  |  |  |  |  |  |  |  |  |  |  | 1171.74 (H2N3-PROC) | 731.13 (H2N2) |  |  |  |  |
| G | 6.29 | 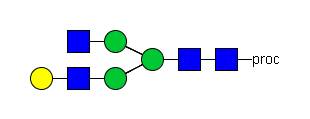 | 4 | 4 | 0 | 0 | 1698.72 | 849.86 | 566.91 | n.d. | 849.97 | n.d. | 441.50 (N1-PROC) | 1171.64 (H2N3-PROC) | 1055.38 (H4N2) |  |  |  |
|  |  |  |  |  |  |  |  |  |  |  |  |  | 644.50 (N2-PROC) | 1333.79 (H3N3-PROC) |  |  |  |  |
|  |  |  |  |  |  |  |  |  |  |  |  |  | 805.51 (H1N2-PROC) | 1495.72 (H4N3-PROC) |  |  |  |  |
|  |  |  |  |  |  |  |  |  |  |  |  |  | 968.63 (H2N2-PROC) | 366.16 (H1N1) |  |  |  |  |
|  |  |  |  |  |  |  |  |  |  |  |  |  | 1130.67 (H3N2-PROC) | 528.00 (H2N1) |  |  |  |  |
| H | 6.46 | 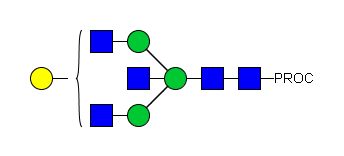 | 4 | 5 | 0 | 0 | 1901.80 | 951.40 | 634.60 | n.d. | 951.49 | 634.68 | 644.38 (N2-PROC) | 1536.80 (H3N4-PROC) |  |  |  |  |
|  |  |  |  |  |  |  |  |  |  |  |  |  | 1009.63 (H1N3-PROC) | 1699.79 (H4N5-PROC) |  |  |  |  |
|  |  |  |  |  |  |  |  |  |  |  |  |  | 1171.77 (H2N3-PROC) | 366.16 (H1N1) |  |  |  |  |
|  |  |  |  |  |  |  |  |  |  |  |  |  | 1333.75 (H3N3-PROC) | 731.63 (H2N2) |  |  |  |  |
|  |  |  |  |  |  |  |  |  |  |  |  |  | 1496.88 (H4N3-PROC) |  |  |  |  |  |
| I | 6.57 | 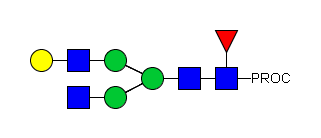 | 4 | 4 | 1 | 0 | 1844.78 | 922.89 | 615.60 | n.d. | 923.01 | 615.67 | 441.38 (N1-PROC) | 968.75 (H2N2-PROC) | 366.21 (H1N1) |  |  |  |
|  |  |  |  |  |  |  |  |  |  |  |  |  | 587.29 (N1F1-PROC) | 1114.61 (H2N2F1-PROC) | 690.50 (H3N1) |  |  |  |
|  |  |  |  |  |  |  |  |  |  |  |  |  | 644.25 (N2-PROC) | 1276.68 (H3N2F1-PROC) | 852.25 (H4N1) |  |  |  |
|  |  |  |  |  |  |  |  |  |  |  |  |  | 790.38 (N2F1-PROC) | 1479.81 (H3N3F1-PROC) |  |  |  |  |
|  |  |  |  |  |  |  |  |  |  |  |  |  | 807.50 (H1N2-PROC) | 1641.85 (H4N3F1-PROC) |  |  |  |  |
| J | 6.69 | 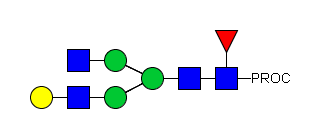 | 4 | 4 | 1 | 0 | 1844.78 | 922.89 | 615.60 | n.d. | 923.00 | 615.67 | 441.33 (N1-PROC) | 968.26 (H2N2-PROC) | 528.13 (H2N1) |  |  |  |
|  |  |  |  |  |  |  |  |  |  |  |  |  | 587.38 (N1F1-PROC) | 1114.68 (H2N2F1-PROC) | 731.25 (H2N2) |  |  |  |
|  |  |  |  |  |  |  |  |  |  |  |  |  | 644.38 (N2-PROC) | 1276.72 (H3N2F1-PROC) | 1055.50 (H4N2) |  |  |  |
|  |  |  |  |  |  |  |  |  |  |  |  |  | 790.50 (N2F1-PROC) | 1479.83 (H3N3F1-PROC) | 1258.75 (H4N3) |  |  |  |
|  |  |  |  |  |  |  |  |  |  |  |  |  | 806.50 (H1N2-PROC) | 366.15 (H1N1) | 1461.63 (H4N4) |  |  |  |
| K | 6.81 | 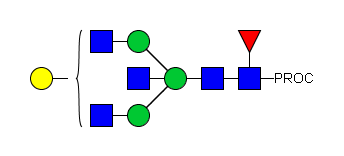 | 4 | 5 | 1 | 0 | 2047.86 | 1024.43 | 683.29 | n.d. | 1024.56 | 683.37 | 441.25 (N1-PROC) | 952.50 (H1N2F1-PROC) | 1479.81 (H3N3F1-PROC) | 1844.88 (H4N4F1-PROC) |  |  |
|  |  |  |  |  |  |  |  |  |  |  |  |  | 587.44 (N1F1-PROC) | 1114.50 (H2N2F1-PROC) | 1521.79 (H2N4F1-PROC) | 366.22 (H1N1) |  |  |
|  |  |  |  |  |  |  |  |  |  |  |  |  | 644.26 (N2-PROC) | 1155.88 (H1N3F1-PROC) | 1536.75 (H3N4-PROC) | 512.25 (H1N1F1) |  |  |
|  |  |  |  |  |  |  |  |  |  |  |  |  | 790.52 (N2F1-PROC) | 1171.75 (H2N3-PROC) | 1641.88 (H4N3F1-PROC) |  |  |  |
|  |  |  |  |  |  |  |  |  |  |  |  |  | 806.13 (H1N2-PROC) | 1317.72 (H2N3F1-PROC) | 1682.93 (H3N4F1-PROC) |  |  |  |
| L | 6.92 | 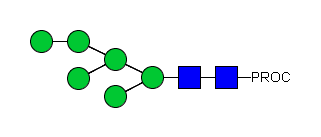 | 6 | 2 | 0 | 0 | 1616.67 | 808.84 | 539.56 | 1616.78 | 808.93 | n.d. | 441.29 (N1-PROC) | 690.30 (H3N1) |  |  |  |  |
|  |  |  |  |  |  |  |  |  |  |  |  |  | 644.39 (N2-PROC) | 852.37 (H4N1) |  |  |  |  |
|  |  |  |  |  |  |  |  |  |  |  |  |  | 968.51 (H2N2-PROC) | 1014.59 (H5N1) |  |  |  |  |
|  |  |  |  |  |  |  |  |  |  |  |  |  | 366.04 (H1N1) | 1176.64 (H6N1) |  |  |  |  |
|  |  |  |  |  |  |  |  |  |  |  |  |  | 528.27 (H2N1) |  |  |  |  |  |
| M | 7.09 | 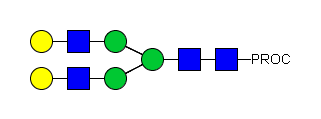 | 5 | 4 | 0 | 0 | 1860.77 | 930.89 | 620.93 | n.d. | 931.00 | 621.00 | 441.38 (N1-PROC) | 1495.79 (H4N3-PROC) |  |  |  |  |
|  |  |  |  |  |  |  |  |  |  |  |  |  | 644.38 (N2-PROC) | 1658.00 (H5N3-PROC) |  |  |  |  |
|  |  |  |  |  |  |  |  |  |  |  |  |  | 805.88 (H1N2-PROC) | 366.16 (H1N1) |  |  |  |  |
|  |  |  |  |  |  |  |  |  |  |  |  |  | 968.74 (H2N2-PROC) | 528.38 (H2N1) |  |  |  |  |
|  |  |  |  |  |  |  |  |  |  |  |  |  | 1292.75 (H4N2-PROC) |  |  |  |  |  |
| N | 7.26 | 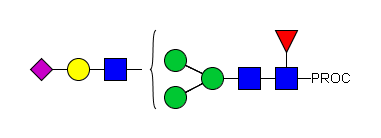 | 4 | 3 | 1 | 1 | 1932.79 | 966.90 | 644.94 | n.d. | 966.99 | n.d. | 790.75 (N2F1-PROC) | 366.23 (H1N1) |  |  |  |  |
|  |  |  |  |  |  |  |  |  |  |  |  |  | 952.88 (H1N2F1-PROC) | 657.26 (H1N1S1) |  |  |  |  |
|  |  |  |  |  |  |  |  |  |  |  |  |  | 1114.52 (H2N2F1-PROC) |  |  |  |  |  |
|  |  |  |  |  |  |  |  |  |  |  |  |  | 1276.69 (H3N2F1-PROC) |  |  |  |  |  |
|  |  |  |  |  |  |  |  |  |  |  |  |  | 1641.88 (H4N3F1-PROC) |  |  |  |  |  |
| N | 7.26 | 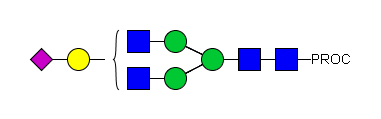 | 4 | 4 | 0 | 1 | 1989.82 | 995.41 | 663.94 | n.d. | 995.53 | n.d. | 441.25 (N1-PROC) | 1333.80 (H3N3-PROC) | 657.28 (H1N1S1) |  |  |  |
|  |  |  |  |  |  |  |  |  |  |  |  |  | 806.50 (H1N2-PROC) | 1495.75 (H4N3-PROC) | 894.50 (H3N2) |  |  |  |
|  |  |  |  |  |  |  |  |  |  |  |  |  | 968.47 (H2N2-PROC) | 1786.88 (H4N3S1) |  |  |  |  |
|  |  |  |  |  |  |  |  |  |  |  |  |  | 1130.61 (H3N2-PROC) | 366.27 (H1N1) |  |  |  |  |
|  |  |  |  |  |  |  |  |  |  |  |  |  | 1171.79 (H2N3-PROC) | 528.63 (H2N1) |  |  |  |  |
| O | 7.46 | 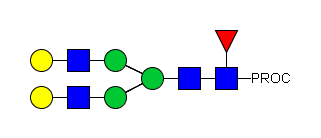 | 5 | 4 | 1 | 0 | 2006.83 | 1003.92 | 669.62 | n.d. | 1004.05 | 669.70 | 441.25 (N1-PROC) | 952.50 (H1N2F1-PROC) | 1334.00 (H3N3-PROC) |  |  |  |
|  |  |  |  |  |  |  |  |  |  |  |  |  | 587.40 (N1F1-PROC) | 968.55 (H2N2-PROC) | 366.20 (H1N1) |  |  |  |
|  |  |  |  |  |  |  |  |  |  |  |  |  | 644.25 (N2-PROC) | 1114.63 (H2N2F1-PROC) | 528.30 (H2N1) |  |  |  |
|  |  |  |  |  |  |  |  |  |  |  |  |  | 790.63 (N2F1-PROC) | 1130.70 (H3N2-PROC) | 893.50 (H3N2) |  |  |  |
|  |  |  |  |  |  |  |  |  |  |  |  |  | 807.25 (H1N2-PROC) | 1276.72 (H3N2F1-PROC) | 1055.47 (H4N2) |  |  |  |
| P | 7.61 | 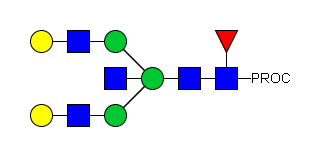 | 5 | 5 | 1 | 0 | 2209.91 | 1105.46 | 737.31 | n.d. | 1105.60 | 737.40 | 441.39 (N1-PROC) | 968.50 (H2N2-PROC) | 1333.74 (H3N3-PROC) | 528.25 (H2N1) |  |  |
|  |  |  |  |  |  |  |  |  |  |  |  |  | 587.28 (N1F1-PROC) | 1009.66 (H1N3-PROC) | 1479.89 (H3N3F1-PROC) | 690.88 (H3N1) |  |  |
|  |  |  |  |  |  |  |  |  |  |  |  |  | 644.38 (N2-PROC) | 1155.75 (H1N3F1-PROC) | 1496.80 (H4N3-PROC) |  |  |  |
|  |  |  |  |  |  |  |  |  |  |  |  |  | 790.63 (N2F1-PROC) | 1172.92 (H2N3-PROC) | 1641.88 (H4N3F1-PROC) |  |  |  |
|  |  |  |  |  |  |  |  |  |  |  |  |  | 806.50 (H1N2-PROC) | 1317.75 (H2N3F1-PROC) | 366.15 (H1N1) |  |  |  |
| Q | 7.69 | 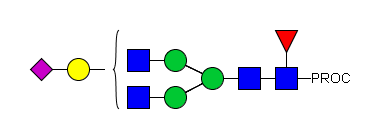 | 4 | 4 | 1 | 1 | 2135.87 | 1068.44 | 712.63 | n.d. | 1068.57 | 712.72 | 441.39 (N1-PROC) | 1276.77 (H3N2F1-PROC) | 657.29 (H1N1S1) |  |  |  |
|  |  |  |  |  |  |  |  |  |  |  |  |  | 587.38 (N1F1-PROC) | 1334.73 (H3N3-PROC) | 689.75 (H3N1) |  |  |  |
|  |  |  |  |  |  |  |  |  |  |  |  |  | 645.38 (N2-PROC) | 1497.00 (H4N3-PROC) | 820.26 (H2N1S1) |  |  |  |
|  |  |  |  |  |  |  |  |  |  |  |  |  | 807.50 (H1N2-PROC) | 366.19 (H1N1) | 893.25 (H3N2) |  |  |  |
|  |  |  |  |  |  |  |  |  |  |  |  |  | 1130.65 (H3N2-PROC) | 529.26 (H2N1) |  |  |  |  |
| R | 7.99 | 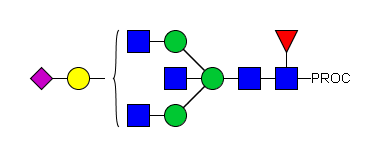 | 4 | 5 | 1 | 1 | 2338.95 | 1169.98 | 780.32 | n.d. | 1170.57 | 780.41 | 441.38 (N1-PROC) | 1155.75 (H1N3F1-PROC) | 1537.75 (H3N4-PROC) |  |  |  |
|  |  |  |  |  |  |  |  |  |  |  |  |  | 587.42 (N1F1-PROC) | 1171.75 (H2N3-PROC) | 366.22 (H1N1) |  |  |  |
|  |  |  |  |  |  |  |  |  |  |  |  |  | 645.13 (N2-PROC) | 1317.67 (H2N3F1-PROC) | 657.07 (H1N1S1) |  |  |  |
|  |  |  |  |  |  |  |  |  |  |  |  |  | 968.73 (H2N2-PROC) | 1333.75 (H3N3-PROC) | 819.63 (H2N1S1) |  |  |  |
|  |  |  |  |  |  |  |  |  |  |  |  |  | 1130.75 (H3N2-PROC) | 1479.85 (H3N3F1-PROC) | 1096.50 (H3N3) |  |  |  |
| S | 8.07 | 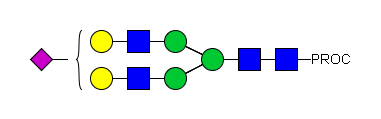 | 5 | 4 | 0 | 1 | 2151.87 | 1076.44 | 717.96 | n.d. | 1076.58 | 718.06 | 441.32 (N1-PROC) | 1292.50 (H4N2-PROC) | 657.27 (H1N1S1) | 1420.63 (H5N3) |  |  |
|  |  |  |  |  |  |  |  |  |  |  |  |  | 644.38 (N2-PROC) | 1333.89 (H3N3-PROC) | 818.13 (H2N1S1) |  |  |  |
|  |  |  |  |  |  |  |  |  |  |  |  |  | 806.50 (H1N2-PROC) | 1495.88 (H4N3-PROC) | 893.93 (H3N2) |  |  |  |
|  |  |  |  |  |  |  |  |  |  |  |  |  | 968.56 (H2N2-PROC) | 366.17 (H1N1) | 1056.50 (H4N2) |  |  |  |
|  |  |  |  |  |  |  |  |  |  |  |  |  | 1130.68 (H3N2-PROC) | 528.25 (H2N1) | 1259.63 (H4N3) |  |  |  |
| T | 8.32 | 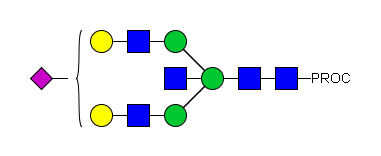 | 5 | 5 | 0 | 1 | 2354.95 | 1177.98 | 785.65 | n.d. | 1178.58 | 785.75 | 441.59 (N1-PROC) | 1171.71 (H2N3-PROC) | 366.18 (H1N1) |  |  |  |
|  |  |  |  |  |  |  |  |  |  |  |  |  | 644.34 (N2-PROC) | 1333.63 (H3N3-PROC) | 657.22 (H1N1S1) |  |  |  |
|  |  |  |  |  |  |  |  |  |  |  |  |  | 951.26 (H1N2F1-PROC) | 1495.63 (H4N3-PROC) | 819.25 (H2N1S1) |  |  |  |
|  |  |  |  |  |  |  |  |  |  |  |  |  | 968.63 (H2N2-PROC) | 1699.00 (H4N4-PROC) |  |  |  |  |
|  |  |  |  |  |  |  |  |  |  |  |  |  | 1009.63 (H1N3-PROC) | 1991.75 (H4N4S1-PROC) |  |  |  |  |
| U | 8.43 | 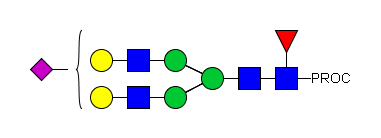 | 5 | 4 | 1 | 1 | 2297.93 | 1149.47 | 766.65 | n.d. | 1150.06 | 766.75 | 441.42 (N1-PROC) | 968.59 (H2N2-PROC) | 1479.88 (H3N3F1-PROC) | 657.26 (H1N1S1) |  |  |
|  |  |  |  |  |  |  |  |  |  |  |  |  | 587.39 (N1F1-PROC) | 1114.50 (H2N2F1-PROC) | 1495.74 (H4N3-PROC) | 821.46 (H2N1S1) |  |  |
|  |  |  |  |  |  |  |  |  |  |  |  |  | 645.50 (N2-PROC) | 1130.66 (H3N2-PROC) | 1641.75 (H4N3F1-PROC) | 894.00 (H3N2) |  |  |
|  |  |  |  |  |  |  |  |  |  |  |  |  | 806.50 (H1N2-PROC) | 1276.77 (H3N2F1-PROC) | 366.18 (H1N1) | 1055.62 (H4N2) |  |  |
|  |  |  |  |  |  |  |  |  |  |  |  |  | 952.62 (H1N2F1-PROC) | 1334.00 (H3N3-PROC) | 528.25 (H2N1) | 1346.50 (H3N2S1) |  |  |
| V | 8.66 | 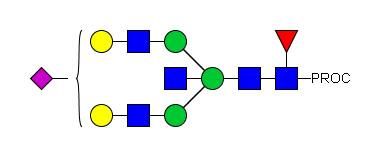 | 5 | 5 | 1 | 1 | 2501.01 | 1251.01 | 834.34 | n.d. | 1251.60 | 834.44 | 441.40 (N1-PROC) | 1130.75 (H3N2-PROC) | 1624.88 (H3N3S1-PROC) | 366.21 (H1N1) | 1258.71 (H4N3) |  |
|  |  |  |  |  |  |  |  |  |  |  |  |  | 587.46 (N1F1-PROC) | 1156.63 (H1N3F1-PROC) | 1641.75 (H4N3F1-PROC) | 731.38 (H2N2) | 1548.42 (H4N3S1) |  |
|  |  |  |  |  |  |  |  |  |  |  |  |  | 644.50 (N2-PROC) | 1317.88 (H2N3F1-PROC) | 1698.88 (H4N4-PROC) | 821.38 (H2N1S1) |  |  |
|  |  |  |  |  |  |  |  |  |  |  |  |  | 968.71 (H2N2-PROC) | 1333.88 (H3N3-PROC) | 1771.25 (H3N3F1S1-PROC) | 894.50 (H3N2) |  |  |
|  |  |  |  |  |  |  |  |  |  |  |  |  | 1114.63 (H2N2F1-PROC) | 1479.78 (H3N3F1-PROC) | 1845.01 (H4N4F1-PROC) | 1055.38 (H4N2) |  |  |
| W | 9.07 | **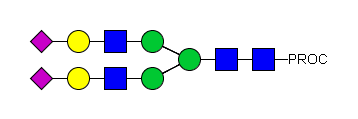** | 5 | 4 | 0 | 2 | 2442.96 | 1221.99 | 814.99 | n.d. | 1222.57 | 815.09 | 441.39 (N1-PROC) | 1292.79 (H4N2-PROC) | 528.13 (H2N1) |  |  |  |
|  |  |  |  |  |  |  |  |  |  |  |  |  | 644.50 (N2-PROC) | 1333.83 (H3N3-PROC) | 894.07 (H3N2) |  |  |  |
|  |  |  |  |  |  |  |  |  |  |  |  |  | 806.38 (H1N2-PROC) | 1495.88 (H4N3-PROC) | 1055.32 (H4N2) |  |  |  |
|  |  |  |  |  |  |  |  |  |  |  |  |  | 968.56 (H2N2-PROC) | 1786.88 (H4N3S1-PROC) | 1346.51 (H4N2S1) |  |  |  |
|  |  |  |  |  |  |  |  |  |  |  |  |  | 1130.76 (H3N2-PROC) | 366.22 (H1N1) |  |  |  |  |
| X | 9.20 | **-** | 5 | 5 | 0 | 2 | 2246.04 | 1323.53 | 882.69 | n.d. | n.d. | 882.79 |  |  |  |  |  |  |
|  |  |  |  |  |  |  |  |  |  |  |  |  |  |  |  |  |  |  |
|  |  |  |  |  |  |  |  |  |  |  |  |  | No MS/MS data detected |  |  |  |  |  |
|  |  |  |  |  |  |  |  |  |  |  |  |  |  |  |  |  |  |  |
|  |  |  |  |  |  |  |  |  |  |  |  |  |  |  |  |  |  |  |
| Y | 9.42 | **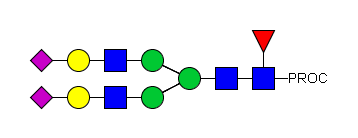** | 5 | 4 | 1 | 2 | 2589.02 | 1295.01 | 863.68 | n.d. | 1295.60 | 863.78 | 441.32 (N1-PROC) | 968.55 (H2N2-PROC) | 1479.76 (H3N3F1-PROC) | 528.05 (H2N1) | 1055.50 (H4N2) |  |
|  |  |  |  |  |  |  |  |  |  |  |  |  | 587.35 (N1F1-PROC) | 1114.70 (H2N2F1-PROC) | 1495.73 (H4N3-PROC) | 657.30 (H1N1S1) | 1346.56 (H4N2S1) |  |
|  |  |  |  |  |  |  |  |  |  |  |  |  | 644.50 (N2-PROC) | 1130.67 (H3N2-PROC) | 1641.91 (H4N3F1-PROC) | 690.50 (H3N1) |  |  |
|  |  |  |  |  |  |  |  |  |  |  |  |  | 748.88 (H1N1F1-PROC) | 1276.72 (H3N2F1-PROC) | 1932.98 (H4N3F1S1-PROC) | 819.35 (H2N1S1) |  |  |
|  |  |  |  |  |  |  |  |  |  |  |  |  | 806.46 (H1N2-PROC) | 1333.75 (H3N3-PROC) | 366.21 (H1N1) | 894.00 (H3N2) |  |  |
| Z | 9.53 | **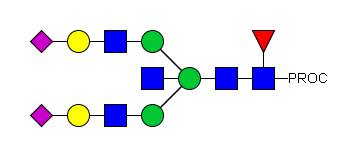** | 5 | 5 | 1 | 2 | 2792.10 | 1396.55 | 931.37 | n.d. | 1397.16 | 931.48 | 441.36 (N1-PROC) | 1130.71 (H3N2-PROC) | 1333.75 (H3N3-PROC) | 2136.10 (H4N4F1S1-PROC) | 894.38 (H3N2) |  |
|  |  |  |  |  |  |  |  |  |  |  |  |  | 587.40 (N1F1-PROC) | 1156.00 (H1N3F1-PROC) | 1479.81 (H3N3F1-PROC) | 366.18 (H1N1) | 1258.50 (H4N3) |  |
|  |  |  |  |  |  |  |  |  |  |  |  |  | 644.47 (N2-PROC) | 1171.90 (H2N3-PROC) | 1642.00 (H4N3F1-PROC) | 528.24 (H2N1) | 1346.75 (H4N2S1) |  |
|  |  |  |  |  |  |  |  |  |  |  |  |  | 806.51 (H1N2-PROC) | 1292.75 (H4N2-PROC) | 1845.93 (H4N4F1-PROC) | 657.29 (H1N1S1) | 1549.62 (H4N3S1) |  |
|  |  |  |  |  |  |  |  |  |  |  |  |  | 967.88 (H2N2-PROC) | 1317.73 (H2N3F1-PROC) | 1933.25 (H4N3F1S1-PROC) | 819.38 (H2N1S1) |  |  |

**Table C.** Structural characterization of procainamide labelled IgG *N*-glycans. Structures for *N*-glycans are depicted following the Consortium for Functional Glycomics (CFG) notation: *N*-acetylglucosamine (N; blue square), fucose (F; red triangle), galactose (H; yellow circle), mannose (H; green circle), *N*-acetylneuraminic acid (S; purple diamond). Glycan compositions are given in the terms of hexose (H), *N*-acetylhexosamine (N), deoxyhexose (F), *N*-acetylneuraminic acid (S).

**PLASMA**

*****

30

35

40

45

50

55

60

**Time [min]**

**A**

**B**

**C**

**D**

**E**

**F**

**G**

**H**

**I**

**K**

**L**

**J**

**M**

**N**

**O**

**P**

**Q**

**R**

**S**

**T**

**U**

**V**

**W**

**X**

**Y**

**Z**

**AA**

**AB**

**AC**

******

| Average | | Possible structure | Composition | | | | Human plasma LC-ESI-MS (in-solution PNGaseF release) | | | | | | | | | | | |
| --- | --- | --- | --- | --- | --- | --- | --- | --- | --- | --- | --- | --- | --- | --- | --- | --- | --- | --- |
| Peak ID | GU (Procainamide) |  |  |  |  |  | [M/Z]^+^ calculated | [M/Z]^2+^ calculated | [M/Z]^3+^ calculated | [M/Z]^+^ registered | [M/Z]^2+^ registered | [M/Z]^3+^ registered | [M/Z] characteristic fragment ions (composition) | | | | | |
|  |  |  | Hex (H) | HexNAc (N) | Fuc (F) | Neu5Ac (S) |  |  |  |  |  |  |  |  |  |  |  |  |
| A | 5.39 | 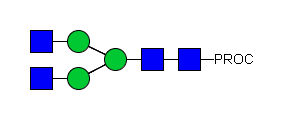 | 3 | 4 | 0 | 0 | 1536.67 | 768.84 | 512.89 | 1536.82 | 768.95 | n.d. | 441.30 (N1-PROC) | 1333.77 (H3N3-PROC) |  |  |  |  |
|  |  |  |  |  |  |  |  |  |  |  |  |  | 644.38 (N2-PROC) | 366.13 (H1N1) |  |  |  |  |
|  |  |  |  |  |  |  |  |  |  |  |  |  | 968.81 (H2N2-PROC) | 528.17 (H2N1) |  |  |  |  |
|  |  |  |  |  |  |  |  |  |  |  |  |  | 1130.73 (H3N2-PROC) | 690.13 (H3N1) |  |  |  |  |
|  |  |  |  |  |  |  |  |  |  |  |  |  | 1171.67 (H2N3-PROC) |  |  |  |  |  |
| B | 5.78 | 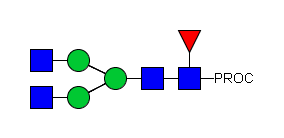 | 3 | 4 | 1 | 0 | 1682.72 | 841.87 | 561.58 | n.d. | 841.97 | n.d. | 441.41 (N1-PROC) | 952.38 (H1N2F1-PROC) | 1317.90 (H2N3F1-PROC) |  |  |  |
|  |  |  |  |  |  |  |  |  |  |  |  |  | 587.39 (N1F1-PROC) | 968.53 (H2N2-PROC) | 1333.77 (H3N3-PROC) |  |  |  |
|  |  |  |  |  |  |  |  |  |  |  |  |  | 644.25 (N2-PROC) | 1114.70 (H2N2F1-PROC) | 1479.83 (H3N3F1-PROC) |  |  |  |
|  |  |  |  |  |  |  |  |  |  |  |  |  | 790.62 (N2F1-PROC) | 1130.60 (H3N2-PROC) | 366.08 (H1N1) |  |  |  |
|  |  |  |  |  |  |  |  |  |  |  |  |  | 806.38 (H1N2-PROC) | 1276.75 (H3N2F1-PROC) |  |  |  |  |
| C | 6.03 | 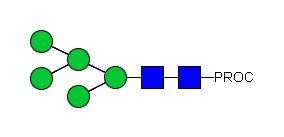 | 5 | 2 | 0 | 0 | 1454.61 | 727.81 | 485.54 | 1454.78 | 727.93 | n.d. | 441.35 (N1-PROC) | 690.07 (H3N1) |  |  |  |  |
|  |  |  |  |  |  |  |  |  |  |  |  |  | 644.32 (N2-PROC) | 852.30 (H4N1) |  |  |  |  |
|  |  |  |  |  |  |  |  |  |  |  |  |  | 806.59 (H1N2-PROC) | 1014.15 (H5N1) |  |  |  |  |
|  |  |  |  |  |  |  |  |  |  |  |  |  | 366.15 (H1N1) |  |  |  |  |  |
|  |  |  |  |  |  |  |  |  |  |  |  |  | 528.22 (H2N1) |  |  |  |  |  |
| D | 6.11 | 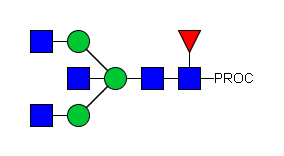 | 3 | 5 | 1 | 0 | 1885.80 | 943.41 | 629.27 | n.d. | 943.54 | 629.36 | 441.38 (N1-PROC) | 1009.76 (H1N3-PROC) | 1333.75 (H3N3-PROC) | 732.50 (H2N2) |  |  |
|  |  |  |  |  |  |  |  |  |  |  |  |  | 587.38 (N1F1-PROC) | 1114.63 (H2N2F1-PROC) | 1375.88 (H2N4-PROC) | 894.50 (H3N2) |  |  |
|  |  |  |  |  |  |  |  |  |  |  |  |  | 644.27 (N2-PROC) | 1172.63 (H2N3-PROC) | 1479.83 (H3N3F1-PROC) | 1097.50 (H3N3) |  |  |
|  |  |  |  |  |  |  |  |  |  |  |  |  | 790.50 (N2F1-PROC) | 1277.88 (H3N2F1-PROC) | 1682.91 (H3N4F1-PROC) |  |  |  |
|  |  |  |  |  |  |  |  |  |  |  |  |  | 968.38 (H2N2-PROC) | 1317.87 (H2N3F1-PROC) | 366.19 (H1N1) |  |  |  |
| D | 6.11 | 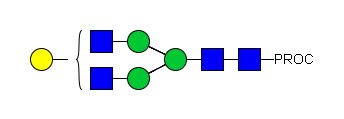 | 4 | 4 | 0 | 0 | 1698.72 | 849.86 | 566.91 | n.d. | 849.97 | 567.00 | 441.27 (N1-PROC) | 1171.71 (H2N3-PROC) | 690.35 (H3N1) |  |  |  |
|  |  |  |  |  |  |  |  |  |  |  |  |  | 644.29 (N2-PROC) | 1333.77 (H3N3-PROC) | 893.29 (H3N2) |  |  |  |
|  |  |  |  |  |  |  |  |  |  |  |  |  | 806.63 (H1N2-PROC) | 1495.77 (H4N3-PROC) | 1055.75 (H4N2) |  |  |  |
|  |  |  |  |  |  |  |  |  |  |  |  |  | 968.63 (H2N2-PROC) | 366.18 (H1N1) | 1096.50 (H3N3) |  |  |  |
|  |  |  |  |  |  |  |  |  |  |  |  |  | 1130.68 (H3N2-PROC) | 529.13 (H2N1) | 1258.26 (H4N3) |  |  |  |
| E | 6.44 | 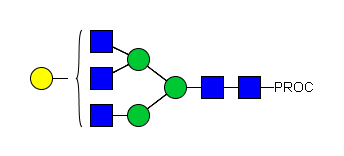 | 4 | 5 | 0 | 0 | 1901.80 | 951.40 | 634.60 | n.d. | 951.51 | 634.68 | 441.31 (N1-PROC) | 1375.75 (H2N4-PROC) | 893.88 (H3N2) |  |  |  |
|  |  |  |  |  |  |  |  |  |  |  |  |  | 644.38 (N2-PROC) | 1536.88 (H3N4-PROC) | 1057.88 (H4N2) |  |  |  |
|  |  |  |  |  |  |  |  |  |  |  |  |  | 806.50 (H1N2-PROC) | 1699.88 (H4N4-PROC) | 1258.50 (H4N3) |  |  |  |
|  |  |  |  |  |  |  |  |  |  |  |  |  | 1171.69 (H2N3-PROC) | 366.23 (H1N1) |  |  |  |  |
|  |  |  |  |  |  |  |  |  |  |  |  |  | 1333.75 (H3N3-PROC) | 528.38 (H2N1) |  |  |  |  |
| F | 6.55 | 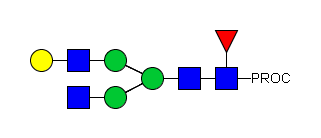 | 4 | 4 | 1 | 0 | 1844.78 | 922.89 | 615.60 | n.d. | 923.02 | 615.68 | 441.38 (N1-PROC) | 968.50 (H2N2-PROC) | 528.23 (H2N1) |  |  |  |
|  |  |  |  |  |  |  |  |  |  |  |  |  | 587.34 (N1F1-PROC) | 1114.69 (H2N2F1-PROC) | 690.39 (H3N1) |  |  |  |
|  |  |  |  |  |  |  |  |  |  |  |  |  | 644.27 (N2-PROC) | 1130.75 (H3N2-PROC) | 893.50 (H3N2) |  |  |  |
|  |  |  |  |  |  |  |  |  |  |  |  |  | 790.50 (N2F1-PROC) | 1276.71 (H3N2F1-PROC) | 1055.38 (H4N2) |  |  |  |
|  |  |  |  |  |  |  |  |  |  |  |  |  | 806.75 (H1N2-PROC) | 366.15 (H1N1) |  |  |  |  |
| G | 6.67 | 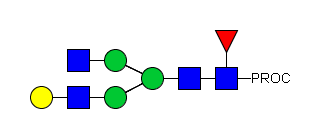 | 4 | 4 | 1 | 0 | 1844.78 | 922.89 | 615.60 | n.d. | 923.02 | 615.68 | 441.38 (N1-PROC) | 1114.71 (H2N2F1-PROC) | 1495.75 (H4N3-PROC) | 1056.63 (H4N2) |  |  |
|  |  |  |  |  |  |  |  |  |  |  |  |  | 588.27 (N1F1-PROC) | 1130.63 (H3N2-PROC) | 1641.93 (H4N3F1-PROC) | 1258.63 (H4N3) |  |  |
|  |  |  |  |  |  |  |  |  |  |  |  |  | 644.38 (N2-PROC) | 1276.72 (H3N2F1-PROC) | 366.18 (H1N1) |  |  |  |
|  |  |  |  |  |  |  |  |  |  |  |  |  | 790.46 (N2F1-PROC) | 1317.83 (H2N3F1-PROC) | 528.19 (H2N1) |  |  |  |
|  |  |  |  |  |  |  |  |  |  |  |  |  | 952.50 (H1N2F1-PROC) | 1479.76 H3N3F1-PROC) | 691.63 (H3N1) |  |  |  |
| H | 6.79 | 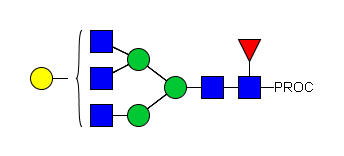 | 4 | 5 | 1 | 0 | 2047.86 | 1024.43 | 683.29 | n.d. | 1024.58 | 683.39 | 441.50 (N1-PROC) | 1130.88 (H3N2-PROC) | 1641.76 (H4N3F1-PROC) |  |  |  |
|  |  |  |  |  |  |  |  |  |  |  |  |  | 587.25 (N1F1-PROC) | 1171.68 (H2N3-PROC) | 366.24 (H1N1) |  |  |  |
|  |  |  |  |  |  |  |  |  |  |  |  |  | 644.63 (N2-PROC) | 1318.64 (H2N3F1-PROC) | 528.13 (H2N1) |  |  |  |
|  |  |  |  |  |  |  |  |  |  |  |  |  | 791.75 (N2F1-PROC) | 1333.88 (H3N3-PROC) | 1096.25 (H3N3) |  |  |  |
|  |  |  |  |  |  |  |  |  |  |  |  |  | 969.50 (H2N2-PROC) | 1479.83 (H3N3F1-PROC) | 1258.50 (H4N3) |  |  |  |
| H | 6.79 | 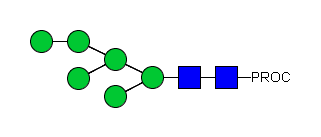 | 6 | 2 | 0 | 0 | 1616.67 | 808.84 | 539.56 | n.d. | 808.93 | n.d. | 441.32 (N1-PROC) | 690.36 (H3N1) |  |  |  |  |
|  |  |  |  |  |  |  |  |  |  |  |  |  | 644.38 (N2-PROC) | 852.32 (H4N1) |  |  |  |  |
|  |  |  |  |  |  |  |  |  |  |  |  |  | 968.63 (H2N2-PROC) | 1014.50 (H5N1) |  |  |  |  |
|  |  |  |  |  |  |  |  |  |  |  |  |  | 366.13 (H1N1) | 1176.56 (H6N1) |  |  |  |  |
|  |  |  |  |  |  |  |  |  |  |  |  |  | 528.04 (H2N1) |  |  |  |  |  |
| H | 6.79 | 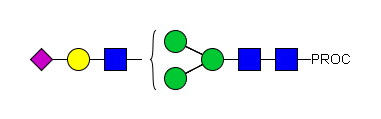 | 4 | 3 | 0 | 1 | 1786.74 | 893.87 | 596.25 | n.d. | 893.98 | 596.32 | 441.28 (N1-PROC) | 1496.75 (H4N3-PROC) |  |  |  |  |
|  |  |  |  |  |  |  |  |  |  |  |  |  | 644.38 (N2-PROC) | 366.18 (H1N1) |  |  |  |  |
|  |  |  |  |  |  |  |  |  |  |  |  |  | 806.25 (H1N2-PROC) | 657.27 (H1N1S1) |  |  |  |  |
|  |  |  |  |  |  |  |  |  |  |  |  |  | 968.62 (H2N2-PROC) |  |  |  |  |  |
|  |  |  |  |  |  |  |  |  |  |  |  |  | 1130.68 (H3N2-PROC) |  |  |  |  |  |
| I | 6.90 | 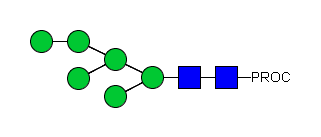 | 6 | 2 | 0 | 0 | 1616.67 | 808.84 | 539.56 | n.d. | 808.95 | n.d. | 441.32 (N1-PROC) | 690.36 (H3N1) |  |  |  |  |
|  |  |  |  |  |  |  |  |  |  |  |  |  | 644.38 (N2-PROC) | 852.32 (H4N1) |  |  |  |  |
|  |  |  |  |  |  |  |  |  |  |  |  |  | 968.63 (H2N2-PROC) | 1014.50 (H5N1) |  |  |  |  |
|  |  |  |  |  |  |  |  |  |  |  |  |  | 366.13 (H1N1) | 1176.56 (H6N1) |  |  |  |  |
|  |  |  |  |  |  |  |  |  |  |  |  |  | 528.04 (H2N1) |  |  |  |  |  |
| J | 7.07 | 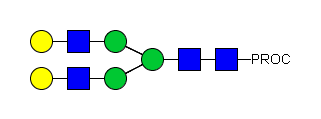 | 5 | 4 | 0 | 0 | 1860.77 | 930.89 | 620.93 | n.d. | 931.00 | 621.01 | 441.39 (N1-PROC) | 1495.76 (H4N3-PROC) | 1056.63 (H4N2) |  |  |  |
|  |  |  |  |  |  |  |  |  |  |  |  |  | 645.13 (N2-PROC) | 366.19 (H1N1) | 1421.69 (H5N3) |  |  |  |
|  |  |  |  |  |  |  |  |  |  |  |  |  | 968.50 (H2N2-PROC) | 528.25 (H2N1) |  |  |  |  |
|  |  |  |  |  |  |  |  |  |  |  |  |  | 1130.63 (H3N2-PROC) | 730.50 (H2N2) |  |  |  |  |
|  |  |  |  |  |  |  |  |  |  |  |  |  | 1333.77 (H3N3-PROC) | 893.38 (H3N2) |  |  |  |  |
| K | 7.30 | 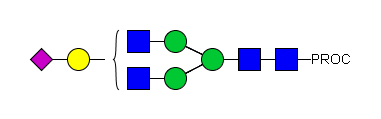 | 4 | 4 | 0 | 1 | 1989.82 | 995.41 | 663.94 | n.d. | 995.54 | 664.03 | 441.25 (N1-PROC) | 1171.64 (H2N3-PROC) | 657.38 (H1N1S1) |  |  |  |
|  |  |  |  |  |  |  |  |  |  |  |  |  | 644.51 (N2-PROC) | 1333.79 (H3N3-PROC) | 820.38 (H2N1S1) |  |  |  |
|  |  |  |  |  |  |  |  |  |  |  |  |  | 806.50 (H1N2-PROC) | 1495.83 (H4N3-PROC) | 893.94 (H3N2) |  |  |  |
|  |  |  |  |  |  |  |  |  |  |  |  |  | 968.60 (H2N2-PROC) | 1788.96 (H4N3S1-PROC) | 1055.50 (H4N2) |  |  |  |
|  |  |  |  |  |  |  |  |  |  |  |  |  | 1130.66 (H3N2-PROC) | 366.26 (H1N1) | 1422.75 (H5N3) |  |  |  |
| L | 7.44 | 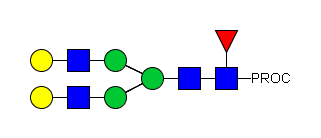 | 5 | 4 | 1 | 0 | 2006.83 | 1003.92 | 669.62 | n.d. | 1004.07 | 669.72 | 441.10 (N1-PROC) | 806.38 (H1N2-PROC) | 366.20 (H1N1) |  |  |  |
|  |  |  |  |  |  |  |  |  |  |  |  |  | 587.45 (N1F1-PROC) | 952.38 (H1N2F1-PROC) | 528.13 (H2N1) |  |  |  |
|  |  |  |  |  |  |  |  |  |  |  |  |  | 644.25 (N2-PROC) | 968.58 (H2N2-PROC) | 690.25 (H3N1) |  |  |  |
|  |  |  |  |  |  |  |  |  |  |  |  |  | 749.50 (H1N1F1-PROC) | 1130.68 (H3N2-PROC) | 893.25 (H3N2) |  |  |  |
|  |  |  |  |  |  |  |  |  |  |  |  |  | 790.38 (N2F1-PROC) | 1333.75 (H3N3-PROC) | 1055.50 (H4N2) |  |  |  |
| M | 7.59 | 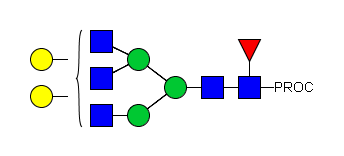 | 5 | 5 | 1 | 0 | 2209.91 | 1105.46 | 737.31 | n.d. | 1105.57 | 737.47 | 441.18 (N1-PROC) | 968.50 (H2N2-PROC) | 1333.75 (H3N3-PROC) | 528.62 (H2N1) |  |  |
|  |  |  |  |  |  |  |  |  |  |  |  |  | 587.38 (N1F1-PROC) | 1114.68 (H2N2F1-PROC) | 1479.69 (H3N3F1-PROC) | 690.38 (H3N1) |  |  |
|  |  |  |  |  |  |  |  |  |  |  |  |  | 644.38 (N2-PROC) | 1171.78 (H2N3-PROC) | 1496.00 (H4N3-PROC) | 1258.93 (H4N3) |  |  |
|  |  |  |  |  |  |  |  |  |  |  |  |  | 748.25 (H1N1F1-PROC) | 1276.63 (H3N2F1-PROC) | 1641.84 (H4N3F1-PROC) |  |  |  |
|  |  |  |  |  |  |  |  |  |  |  |  |  | 790.63 (N2F1-PROC) | 1317.88 (H2N3F1-PROC) | 366.21 (H1N1) |  |  |  |
| N | 7.67 | 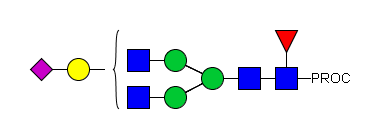 | 4 | 4 | 1 | 1 | 2135.87 | 1068.44 | 712.63 | n.d. | 1068.60 | 712.74 | 441.31 (N1-PROC) | 952.63 (H1N2F1-PROC) | 1334.88 (H3N3-PROC) | 690.50 (H3N1) |  |  |
|  |  |  |  |  |  |  |  |  |  |  |  |  | 587.38 (N1F1-PROC) | 968.00 (H2N2-PROC) | 1481.75 (H3N3F1-PROC) | 893.38 (H3N2) |  |  |
|  |  |  |  |  |  |  |  |  |  |  |  |  | 644.25 (N2-PROC) | 1114.75 (H2N2F1-PROC) | 366.11 (H1N1) |  |  |  |
|  |  |  |  |  |  |  |  |  |  |  |  |  | 790.52 (N2F1-PROC) | 1130.71 (H3N2-PROC) | 527.63 (H2N1) |  |  |  |
|  |  |  |  |  |  |  |  |  |  |  |  |  | 805.38 (H1N2-PROC) | 1276.67 (H3N2F1-PROC) | 657.21 (H1N1S1) |  |  |  |
| O | 7.80 | 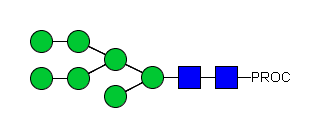 | 7 | 2 | 0 | 0 | 1778.72 | 889.86 | 593.58 | n.d. | 889.95 | n.d. | 441.38 (N1-PROC) | 366.24 (H1N1) | 1177.45 (H6N1) |  |  |  |
|  |  |  |  |  |  |  |  |  |  |  |  |  | 644.30 (N2-PROC) | 527.88 (H2N1) | 1338.78 (H7N1) |  |  |  |
|  |  |  |  |  |  |  |  |  |  |  |  |  | 806.44 (H1N2-PROC) | 690.34 (H3N1) |  |  |  |  |
|  |  |  |  |  |  |  |  |  |  |  |  |  | 968.50 (H2N2-PROC) | 852.45 (H4N1) |  |  |  |  |
|  |  |  |  |  |  |  |  |  |  |  |  |  | 1130.75 (H3N2-PROC) | 1014.58 (H5N1) |  |  |  |  |
| P | 8.05 | 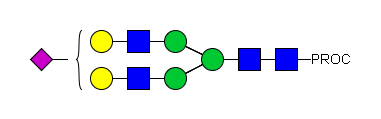 | 5 | 4 | 0 | 1 | 2151.87 | 1076.44 | 717.96 | n.d. | 1076.55 | 718.33 | 441.36 (N1-PROC) | 1333.63 (H3N3-PROC) | 690.38 (H3N1) |  |  |  |
|  |  |  |  |  |  |  |  |  |  |  |  |  | 644.38 (N2-PROC) | 1495.80 (H4N3-PROC) | 893.49 (H3N2) |  |  |  |
|  |  |  |  |  |  |  |  |  |  |  |  |  | 806.63 (H1N2-PROC) | 366.19 (H1N1) | 1055.51 (H4N2) |  |  |  |
|  |  |  |  |  |  |  |  |  |  |  |  |  | 968.59 (H2N2-PROC) | 528.18 (H2N1) | 1346.59 (H4N2S1) |  |  |  |
|  |  |  |  |  |  |  |  |  |  |  |  |  | 1130.70 (H3N2-PROC) | 657.29 (H1N1S1) | 1421.63 (H5N3) |  |  |  |
| Q | 8.31 | 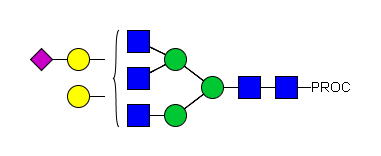 | 5 | 5 | 0 | 1 | 2354.95 | 1177.98 | 785.65 | n.d. | 1178.08 | 758.75 | 441.31 (N1-PROC) | 1171.71 (H2N3-PROC) | 366.21 (H1N1) | 1258.68 (H4N3) |  |  |
|  |  |  |  |  |  |  |  |  |  |  |  |  | 644.38 (N2-PROC) | 1333.80 (H3N3-PROC) | 528.30 (H2N1) | 1347.63 (H4N2S1) |  |  |
|  |  |  |  |  |  |  |  |  |  |  |  |  | 806.63 (H1N2-PROC) | 1495.72 (H4N3-PROC) | 657.29 (H1N1S1) | 1549.63 (H4N3S1) |  |  |
|  |  |  |  |  |  |  |  |  |  |  |  |  | 968.50 (H2N2-PROC) | 1624.75 (H3N3S1-PROC) | 691.88 (H3N1) |  |  |  |
|  |  |  |  |  |  |  |  |  |  |  |  |  | 1130.77 (H3N2-PROC) | 1699.77 (H4N4-PROC) | 1096.42 (H3N3) |  |  |  |
| R | 8.42 | 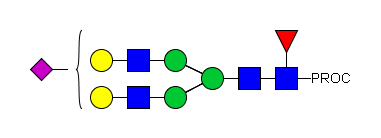 | 5 | 4 | 1 | 1 | 2297.93 | 1149.47 | 766.65 | n.d. | 1150.07 | 766.93 | 441.33 (N1-PROC) | 1114.75 (H2N2F1-PROC) | 1438.77 (H4N2F1-PROC) | 528.25 (H2N1) | 1346.59 (H4N2S1) |  |
|  |  |  |  |  |  |  |  |  |  |  |  |  | 587.43 (N1F1-PROC) | 1130.68 (H3N2-PROC) | 1480.86 (H3N3F1-PROC) | 657.27 (H1N1S1) |  |  |
|  |  |  |  |  |  |  |  |  |  |  |  |  | 644.38 (N2-PROC) | 1171.88 (H2N3-PROC) | 1495.95 (H4N3-PROC) | 894.45 (H3N2) |  |  |
|  |  |  |  |  |  |  |  |  |  |  |  |  | 806.50 (H1N2-PROC) | 1276.74 (H3N2F1-PROC) | 1641.82 (H4N3F1-PROC) | 1055.57 (H4N2) |  |  |
|  |  |  |  |  |  |  |  |  |  |  |  |  | 968.58 (H2N2-PROC) | 1333.63 (H3N3-PROC) | 366.20 (H1N1) | 1184.38 (H3N2S1) |  |  |
| S | 8.65 | **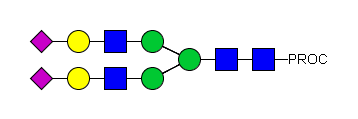** | 5 | 4 | 0 | 2 | 2442.96 | 1221.99 | 814.99 | n.d. | 1222.11 | 815.12 | 441.36 (N1-PROC) | 1333.81 (H3N3-PROC) | 657.28 (H1N1S1) | 1421.75 (H5N3) |  |  |
|  |  |  |  |  |  |  |  |  |  |  |  |  | 644.50 (N2-PROC) | 1495.82 (H4N3-PROC) | 690.38 (H3N1) | 1624.75 (H5N4) |  |  |
|  |  |  |  |  |  |  |  |  |  |  |  |  | 806.50 (H1N2-PROC) | 1786.96 (H4N3S1-PROC) | 893.95 (H3N2) |  |  |  |
|  |  |  |  |  |  |  |  |  |  |  |  |  | 968.61 (H2N2-PROC) | 366.22 (H1N1) | 1056.50 (H4N2) |  |  |  |
|  |  |  |  |  |  |  |  |  |  |  |  |  | 1130.69 (H3N2-PROC) | 528.19 (H2N1) | 1259.75 (H4N3) |  |  |  |
| S | 8.65 | **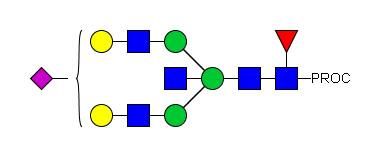** | 5 | 5 | 1 | 1 | 2501.01 | 1251.01 | 834.34 | n.d. | n.d. | 834.45 | 441.32 (N1-PROC) | 1114.67 (H2N2F1-PROC) | 1317.78 (H2N3F1-PROC) | 2007.13 (H5N4F1-PROC) | 893.38 (H3N2) |  |
|  |  |  |  |  |  |  |  |  |  |  |  |  | 587.42 (N1F1-PROC) | 1131.63 (H3N2-PROC) | 1333.80 (H3N3-PROC) | 366.21 (H1N1) | 1096.54 (H3N3) |  |
|  |  |  |  |  |  |  |  |  |  |  |  |  | 644.38 (N2-PROC) | 1155.63 (H1N3F1-PROC) | 1479.82 (H3N3F1-PROC) | 528.18 (H2N1) | 1258.61 (H4N3) |  |
|  |  |  |  |  |  |  |  |  |  |  |  |  | 806.50 (H1N2-PROC) | 1171.74 (H2N3-PROC) | 1644.00 (H4N3F1-PROC) | 657.30 (H1N1S1) | 1549.75 (H4N3S1) |  |
|  |  |  |  |  |  |  |  |  |  |  |  |  | 968.68 (H2N2-PROC) | 1276.67 (H3N2F1-PROC) | 1845.92 (H4N4F1-PROC) | 690.25 (H3N1) |  |  |
| T | 9.05 | **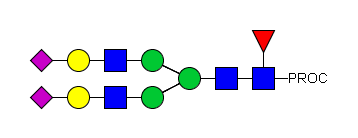** | 5 | 4 | 1 | 2 | 2589.02 | 1295.01 | 863.68 | n.d. | n.d. | 863.74 | 441.38 (N1-PROC) | 969.63 (H2N2-PROC) | 1479.85 (H3N3F1-PROC) | 528.38 (H2N1) |  |  |
|  |  |  |  |  |  |  |  |  |  |  |  |  | 588.38 (N1F1-PROC) | 1114.68 (H2N2F1-PROC) | 1495.91 (H4N3-PROC) | 657.28 (H1N1S1) |  |  |
|  |  |  |  |  |  |  |  |  |  |  |  |  | 644.38 (N2-PROC) | 1131.63 (H3N2-PROC) | 1642.86 (H4N3F1-PROC) | 893.98 (H3N2) |  |  |
|  |  |  |  |  |  |  |  |  |  |  |  |  | 790.57 (N2F1-PROC) | 1276.80 (H3N2F1-PROC) | 1934.25 (H4N3F1S1-PROC) | 1055.25 (H4N2) |  |  |
|  |  |  |  |  |  |  |  |  |  |  |  |  | 952.51 (H1N2F1-PROC) | 1334.88 (H3N3-PROC) | 366.19 (H1N1) | 1346.63 (H4N2S1) |  |  |
| T | 9.05 | **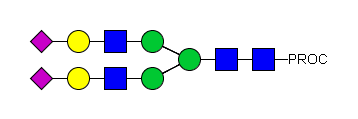** | 5 | 4 | 0 | 2 | 2442.96 | 1221.99 | 814.99 | n.d. | 1222.09 | 815.43 | 441.37 (N1-PROC) | 1333.77 (H3N3-PROC) | 657.31 (H1N1S1) |  |  |  |
|  |  |  |  |  |  |  |  |  |  |  |  |  | 644.50 (N2-PROC) | 1495.82 (H4N3-PROC) | 691.38 (H3N1) |  |  |  |
|  |  |  |  |  |  |  |  |  |  |  |  |  | 806.47 (H1N2-PROC) | 1786.98 (H4N3S1-PROC) | 894.01 (H3N2) |  |  |  |
|  |  |  |  |  |  |  |  |  |  |  |  |  | 968.63 (H2N2-PROC) | 366.22 (H1N1) | 1056.50 (H4N2) |  |  |  |
|  |  |  |  |  |  |  |  |  |  |  |  |  | 1130.73 (H3N2-PROC) | 528.19 (H2N1) | 1346.63 (H4N2S1) |  |  |  |
| U | 9.40 | **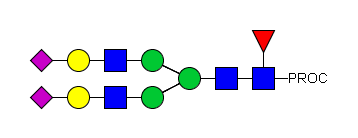** | 5 | 4 | 1 | 2 | 2589.02 | 1295.01 | 863.68 | n.d. | 1295.63 | 863.94 | 441.38 (N1-PROC) | 1114.75 (H2N2F1-PROC) | 1642.04 (H4N3F1-PROC) | 657.32 (H1N1S1) |  |  |
|  |  |  |  |  |  |  |  |  |  |  |  |  | 588.38 (N1F1-PROC) | 1131.61 (H3N2-PROC) | 1787.00 (H4N3S1-PROC) | 691.25 (H3N1) |  |  |
|  |  |  |  |  |  |  |  |  |  |  |  |  | 644.50 (N2-PROC) | 1277.69 (H3N2F1-PROC) | 1933.12 (H4N3F1S1-PROC) | 852.38 (H4N1) |  |  |
|  |  |  |  |  |  |  |  |  |  |  |  |  | 806.63 (H1N2-PROC) | 1333.88 (H3N3-PROC) | 366.22 (H1N1) | 1057.38 (H4N2) |  |  |
|  |  |  |  |  |  |  |  |  |  |  |  |  | 968.55 (H2N2-PROC) | 1495.76 (H4N3-PROC) | 528.25 (H2N1) | 1420.74 (H5N3) |  |  |
| U | 9.40 | **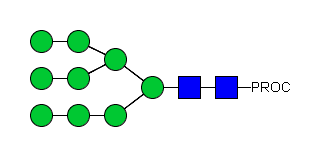** | 9 | 2 | 0 | 0 | 2102.83 | 1051.92 | 701.61 | n.d. | 1052.08 | n.d. | 441.31 (N1-PROC) | 1292.68 (H4N2-PROC) | 852.49 (H4N1) |  |  |  |
|  |  |  |  |  |  |  |  |  |  |  |  |  | 644.38 (N2-PROC) | 1454.63 (H5N2-PROC) | 1014.31 (H5N1) |  |  |  |
|  |  |  |  |  |  |  |  |  |  |  |  |  | 806.50 (H1N2-PROC) | 366.25 (H1N1) | 1176.52 (H6N1) |  |  |  |
|  |  |  |  |  |  |  |  |  |  |  |  |  | 968.63 (H2N2-PROC) | 528.15 (H2N1) | 1338.70 (H7N1) |  |  |  |
|  |  |  |  |  |  |  |  |  |  |  |  |  | 1130.69 (H3N2-PROC) | 690.27 (H3N1) | 1500.75 (H8N1) |  |  |  |
| V | 9.52 | **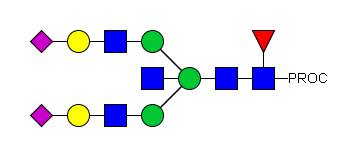** | 5 | 5 | 1 | 2 | 2792.10 | 1396.55 | 931.37 | n.d. | 1397.19 | 931.83 | 441.38 (N1-PROC) | 1009.50 (H1N3-PROC) | 1276.79 (H3N2F1-PROC) | 1642.71 (H4N3F1-PROC) | 657.33 (H1N1S1) | 1387.13 (H3N3S1) |
|  |  |  |  |  |  |  |  |  |  |  |  |  | 587.36 (N1F1-PROC) | 1114.87 (H2N2F1-PROC) | 1317.79 (H2N3F1-PROC) | 1788.27 (H4N3S1-PROC) | 690.75 (H3N1) | 1550.75 (H4N3S1) |
|  |  |  |  |  |  |  |  |  |  |  |  |  | 644.38 (N2-PROC) | 1130.63 (H3N2-PROC) | 1333.75 (H3N3-PROC) | 1935.13 (H4N3F1S1-PROC) | 893.38 (H3N2) |  |
|  |  |  |  |  |  |  |  |  |  |  |  |  | 790.39 (N2F1-PROC) | 1155.63 (H1N3F1-PROC) | 1479.88 (H3N3F1-PROC) | 366.15 (H1N1) | 1097.13 (H3N3) |  |
|  |  |  |  |  |  |  |  |  |  |  |  |  | 968.50 (H2N2-PROC) | 1171.63 (H2N3-PROC) | 1496.63 (H4N3-PROC) | 528.13 (H2N1) | 1346.63 (H4N2S1) |  |
| W | 9.73 | **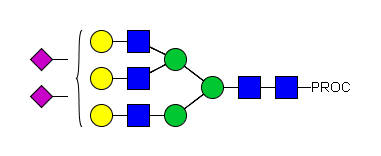** | 6 | 5 | 0 | 2 | 2808.10 | 1404.55 | 936.70 | n.d. | 1404.63 | 936.83 | 441.29 (N1-PROC) | 1171.63 (H2N3-PROC) | 1786.92 (H4N3S1-PROC) | 819.38 (H2N1S1) |  |  |
|  |  |  |  |  |  |  |  |  |  |  |  |  | 644.75 (N2-PROC) | 1333.77 (H3N3-PROC) | 1990.00 (H4N4S1-PROC) | 893.38 (H3N2) |  |  |
|  |  |  |  |  |  |  |  |  |  |  |  |  | 806.50 (H1N2-PROC) | 1495.79 (H4N3-PROC) | 366.21 (H1N1) | 1184.00 (H3N2S1) |  |  |
|  |  |  |  |  |  |  |  |  |  |  |  |  | 968.56 (H2N2-PROC) | 1624.88 (H3N3S1-PROC) | 528.25 (H2N1) |  |  |  |
|  |  |  |  |  |  |  |  |  |  |  |  |  | 1130.75 (H3N2-PROC) | 1698.76 (H4N4-PROC) | 657.22 (H1N1S1) |  |  |  |
| X | 10.11 | **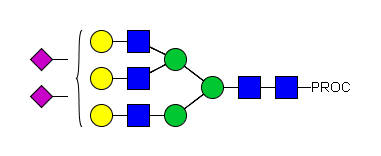** | 6 | 5 | 0 | 2 | 2808.10 | 1404.55 | 936.70 | n.d. | n.d. | 936.82 | 441.28 (N1-PROC) | 1333.63 (H3N3-PROC) | 366.20 (H1N1) | 1346.63 (H4N2S1) |  |  |
|  |  |  |  |  |  |  |  |  |  |  |  |  | 644.25 (N2-PROC) | 1495.78 (H4N3-PROC) | 657.31 (H1N1S1) | 1420.75 (H5N3) |  |  |
|  |  |  |  |  |  |  |  |  |  |  |  |  | 806.48 (H1N2-PROC) | 1787.98 (H4N3S1-PROC) | 819.63 (H2N1S1) | 1799.88 (H5N2S2) |  |  |
|  |  |  |  |  |  |  |  |  |  |  |  |  | 968.62 (H2N2-PROC) | 1860.98 (H5N4-PROC) | 893.38 (H3N2) |  |  |  |
|  |  |  |  |  |  |  |  |  |  |  |  |  | 1130.75 (H3N2-PROC) | 2153.13 (H5N4S1-PROC) | 1184.63 (H3N2S1) |  |  |  |
| Y | 10.30 | **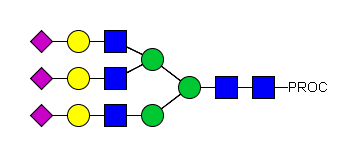** | 6 | 5 | 0 | 3 | 3099.19 | 1550.10 | 1033.74 | n.d. | n.d. | 1033.89 | 441.25 (N1-PROC) | 1333.75 (H3N3-PROC) | 528.00 (H2N1) |  |  |  |
|  |  |  |  |  |  |  |  |  |  |  |  |  | 644.38 (N2-PROC) | 1495.99 (H4N3-PROC) | 657.21 (H1N1S1) |  |  |  |
|  |  |  |  |  |  |  |  |  |  |  |  |  | 806.50 (H1N2-PROC) | 1786.92 (H4N3S1-PROC) | 1055.13 (H4N2) |  |  |  |
|  |  |  |  |  |  |  |  |  |  |  |  |  | 968.60 (H2N2-PROC) | 1990.00 (H4N4S1-PROC) | 1347.51 (H4N2S1) |  |  |  |
|  |  |  |  |  |  |  |  |  |  |  |  |  | 1131.50 (H3N2-PROC) | 366.22 (H1N1) | 1475.63 (H3N2S2) |  |  |  |
| Z | 10.69 | **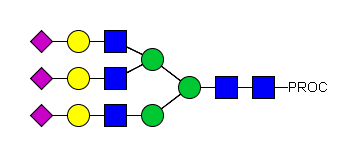** | 6 | 5 | 0 | 3 | 3099.19 | 1550.10 | 1033.74 | n.d. | n.d. | 1033.85 | 441.36 (N1-PROC) | 1333.63 (H3N3-PROC) | 2152.86 (H5N4S1-PROC) | 1184.75 (H3N2S1) |  |  |
|  |  |  |  |  |  |  |  |  |  |  |  |  | 644.25 (N2-PROC) | 1495.75 (H4N3-PROC) | 366.25 (H1N1) | 1347.88 (H4N2S1) |  |  |
|  |  |  |  |  |  |  |  |  |  |  |  |  | 806.61 (H1N2-PROC) | 1624.88 (H3N3S1-PROC) | 657.32 (H1N1S1) | 1476.50 (H3N2S2) |  |  |
|  |  |  |  |  |  |  |  |  |  |  |  |  | 969.61 (H2N2-PROC) | 1699.00 (H4N4-PROC) | 893.25 (H3N2) |  |  |  |
|  |  |  |  |  |  |  |  |  |  |  |  |  | 1130.63 (H3N2-PROC) | 1786.94 (H4N3S1-PROC) | 1056.50 (H4N2) |  |  |  |
| AA | 11.12 | **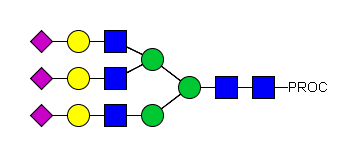** | 6 | 5 | 0 | 3 | 3099.19 | 1550.10 | 1033.74 | n.d. | n.d. | 1033.87 | 441.41 (N1-PROC) | 1333.80 (H3N3-PROC) | 2152.25 (H5N4S1-PROC) |  |  |  |
|  |  |  |  |  |  |  |  |  |  |  |  |  | 644.38 (N2-PROC) | 1495.76 (H4N3-PROC) | 366.11 (H1N1) |  |  |  |
|  |  |  |  |  |  |  |  |  |  |  |  |  | 806.47 (H1N2-PROC) | 1624.94 (H3N3S1-PROC) | 657.28 (H1N1S1) |  |  |  |
|  |  |  |  |  |  |  |  |  |  |  |  |  | 968.77 (H2N2-PROC) | 1698.88 (H4N4-PROC) | 1184.63 (H3N2S1) |  |  |  |
|  |  |  |  |  |  |  |  |  |  |  |  |  | 1130.63 (H3N2-PROC) | 1786.95 (H4N3S1-PROC) | 1476.51 (H3N2S2) |  |  |  |
| AB | 11.23 | **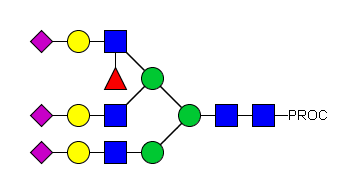** | 6 | 5 | 1 | 3 | 3245.25 | 1623.13 | 1082.42 | n.d. | n.d. | 1082.55 | 441.31 (N1-PROC) | 1495.81 (H4N3-PROC) | 1990.00 (H4N4S1) | 528.24 (H2N1) |  |  |
|  |  |  |  |  |  |  |  |  |  |  |  |  | 644.37 (N2-PROC) | 1624.90 (H3N3S1-PROC) | 2152.00 (H5N4S1-PROC) | 657.24 (H1N1S1) |  |  |
|  |  |  |  |  |  |  |  |  |  |  |  |  | 806.51 (H1N2-PROC) | 1770.00 (H3N3F1S1-PROC) | 2298.21 (H5N4F1S1-PROC) | 803.35 (H1N1F1S1) |  |  |
|  |  |  |  |  |  |  |  |  |  |  |  |  | 968.57 (H2N2-PROC) | 1787.03 (H4N3S1-PROC) | 366.14 (H1N1) | 1184.58 (H3N2S1) |  |  |
|  |  |  |  |  |  |  |  |  |  |  |  |  | 1333.79 (H3N3-PROC) | 1932.95 (H4N3F1S1-PROC) | 512.13 (H1N1F1) | 1475.66 (H3N2S2) |  |  |
| AC | 11.95 | **-** | 7 | 6 | 0 | 4 | 3755.42 | 1878.21 | 1252.48 | n.d. | n.d. | 1252.92 |  |  |  |  |  |  |
|  |  |  |  |  |  |  |  |  |  |  |  |  |  |  |  |  |  |  |
|  |  |  |  |  |  |  |  |  |  |  |  |  | No MS/MS data detected |  |  |  |  |  |
|  |  |  |  |  |  |  |  |  |  |  |  |  |  |  |  |  |  |  |
|  |  |  |  |  |  |  |  |  |  |  |  |  |  |  |  |  |  |  |

**Table D.** Structural characterization of procainamide labelled plasma *N-*glycans. Structures for *N-*glycans are depicted following the Consortium for Functional Glycomics (CFG) notation: *N*-acetylglucosamine (N; blue square), fucose (F; red triangle), galactose (H; yellow circle), mannose (H; green circle), *N*-acetylneuraminic acid (S; purple diamond). Glycan compositions are given in the terms of hexose (H), *N*-acetylhexosamine (N), deoxyhexose (F), *N*-acetylneuraminic acid (S). *^,^ **No MS data detected.

**HT29**

10

15

20

25

30

35

40

45

50

55

60

**Time [min]**

**A**

**B**

**C**

**D**

**E**

**F**

**G**

**H**

**I**

**J**

**K**

**L**

**M**

**N**

**O**

**P**

**Q**

**R**

**S**

**T**

*****

******

*******

********

**AA**

**AD**

**V**

**U**

**Z**

**Y**

**AB**

**AC**

**X**

**W**

| Average | | Possible structure | Composition | | | | HT29 human colorectal cancer cell line LC-ESI-MS (in-solution PNGaseF release) | | | | | | | | | | | |
| --- | --- | --- | --- | --- | --- | --- | --- | --- | --- | --- | --- | --- | --- | --- | --- | --- | --- | --- |
| Peak ID | GU (Procainamide) |  |  |  |  |  | [M/Z]^+^ calculated | [M/Z]^2+^ calculated | [M/Z]^3+^ calculated | [M/Z]^+^ registered | [M/Z]^2+^ registered | [M/Z]^3+^ registered | [M/Z] characteristic fragment ions (composition) | | | | | |
|  |  |  | Hex (H) | HexNAc (N) | Fuc (F) | Neu5Ac (S) |  |  |  |  |  |  |  |  |  |  |  |  |
| A | 3.28 | 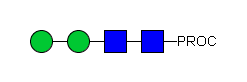 | 2 | 2 | 0 | 0 | 968.46 | 484.73 | 323.49 | 968.61 | 848.81 | n.d. | 441.34 (N1-PROC) |  |  |  |  |  |
|  |  |  |  |  |  |  |  |  |  |  |  |  | 644.39 (N2-PROC) |  |  |  |  |  |
|  |  |  |  |  |  |  |  |  |  |  |  |  | 806.42 (H1N2-PROC) |  |  |  |  |  |
|  |  |  |  |  |  |  |  |  |  |  |  |  |  |  |  |  |  |  |
|  |  |  |  |  |  |  |  |  |  |  |  |  |  |  |  |  |  |  |
| B | 3.69 | 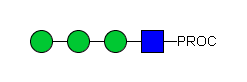 | 3 | 1 | 0 | 0 | 927.43 | 464.22 | 309.81 | 927.56 | 464.28 | n.d. | 441.44 (N1-PROC) |  |  |  |  |  |
|  |  |  |  |  |  |  |  |  |  |  |  |  | 603.40 (H1N1-PROC) |  |  |  |  |  |
|  |  |  |  |  |  |  |  |  |  |  |  |  | 765.43 (H2N1-PROC) |  |  |  |  |  |
|  |  |  |  |  |  |  |  |  |  |  |  |  |  |  |  |  |  |  |
|  |  |  |  |  |  |  |  |  |  |  |  |  |  |  |  |  |  |  |
| C | 3.76 | 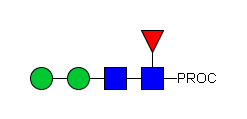 | 2 | 2 | 1 | 0 | 1114.51 | 557.76 | 372.18 | 1114.68 | 557.84 | n.d. | 441.33 (N1-PROC) | 368.22 (H1N1) |  |  |  |  |
|  |  |  |  |  |  |  |  |  |  |  |  |  | 587.35 (N1F1-PROC) | 528.23 (H2N1) |  |  |  |  |
|  |  |  |  |  |  |  |  |  |  |  |  |  | 644.38 (N2-PROC) |  |  |  |  |  |
|  |  |  |  |  |  |  |  |  |  |  |  |  | 806.47 (H1N2-PROC) |  |  |  |  |  |
|  |  |  |  |  |  |  |  |  |  |  |  |  | 968.55 (H2N2-PROC) |  |  |  |  |  |
| D | 3.81 | 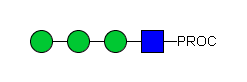 | 3 | 1 | 0 | 0 | 927.43 | 464.22 | 309.81 | 927.55 | 464.34 | n.d. | 441.29 (N1-PROC) |  |  |  |  |  |
|  |  |  |  |  |  |  |  |  |  |  |  |  | 603.31 (H1N1-PROC) |  |  |  |  |  |
|  |  |  |  |  |  |  |  |  |  |  |  |  | 765.44 (H2N1-PROC) |  |  |  |  |  |
|  |  |  |  |  |  |  |  |  |  |  |  |  |  |  |  |  |  |  |
|  |  |  |  |  |  |  |  |  |  |  |  |  |  |  |  |  |  |  |
| E | 4.24 | 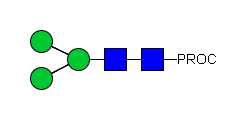 | 3 | 2 | 0 | 0 | 1130.51 | 565.76 | 377.51 | 1130.68 | 565.87 | n.d. | 441.40 (N1-PROC) | 528.13 (H2N1) |  |  |  |  |
|  |  |  |  |  |  |  |  |  |  |  |  |  | 644.37 (N2-PROC) |  |  |  |  |  |
|  |  |  |  |  |  |  |  |  |  |  |  |  | 806.48 (H1N2-PROC) |  |  |  |  |  |
|  |  |  |  |  |  |  |  |  |  |  |  |  | 968.49 (H2N2-PROC) |  |  |  |  |  |
|  |  |  |  |  |  |  |  |  |  |  |  |  | 368.23 (H1N1) |  |  |  |  |  |
| F | 4.69 | 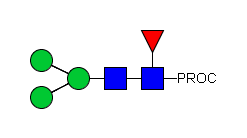 | 3 | 2 | 1 | 0 | 1276.57 | 638.79 | 426.19 | 1276.76 | 638.88 | n.d. | 441.40 (N1-PROC) | 1130.63 (H3N2-PROC) |  |  |  |  |
|  |  |  |  |  |  |  |  |  |  |  |  |  | 587.35 (N1F1-PROC) | 368.20 (H1N1) |  |  |  |  |
|  |  |  |  |  |  |  |  |  |  |  |  |  | 644.38 (N2-PROC) | 528.50 (H2N1) |  |  |  |  |
|  |  |  |  |  |  |  |  |  |  |  |  |  | 806.48 (H1N2-PROC) |  |  |  |  |  |
|  |  |  |  |  |  |  |  |  |  |  |  |  | 968.65 (H2N2-PROC) |  |  |  |  |  |
| G | 4.85 | 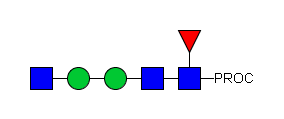 | 2 | 3 | 1 | 0 | 1317.59 | 659.30 | 439.87 | 1317.77 | 659.40 | n.d. | 441.13 (N1-PROC) |  |  |  |  |  |
|  |  |  |  |  |  |  |  |  |  |  |  |  | 643.88 (N2-PROC) |  |  |  |  |  |
|  |  |  |  |  |  |  |  |  |  |  |  |  | 789.50 (N2F1-PROC) |  |  |  |  |  |
|  |  |  |  |  |  |  |  |  |  |  |  |  | 968.75 (H2N2-PROC) |  |  |  |  |  |
|  |  |  |  |  |  |  |  |  |  |  |  |  | 1114.74 (H2N2F1-PROC) |  |  |  |  |  |
| H | 5.06 | 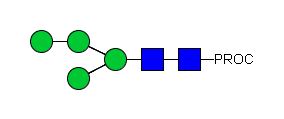 | 4 | 2 | 0 | 0 | 1292.56 | 646.78 | 431.53 | 1292.68 | 646.88 | n.d. | 441.45 (N1-PROC) | 368.34 (H1N1) |  |  |  |  |
|  |  |  |  |  |  |  |  |  |  |  |  |  | 645.38 (N2-PROC) | 528.25 (H2N1) |  |  |  |  |
|  |  |  |  |  |  |  |  |  |  |  |  |  | 806.44 (H1N2-PROC) |  |  |  |  |  |
|  |  |  |  |  |  |  |  |  |  |  |  |  | 968.55 (H2N2-PROC) |  |  |  |  |  |
|  |  |  |  |  |  |  |  |  |  |  |  |  | 1131.54 (H3N2-PROC) |  |  |  |  |  |
| I | 5.13 | 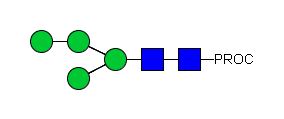 | 4 | 2 | 0 | 0 | 1292.56 | 646.78 | 431.53 | 1292.70 | 646.68 | n.d. | 441.57 (N1-PROC) |  |  |  |  |  |
|  |  |  |  |  |  |  |  |  |  |  |  |  | 644.33 (N2-PROC) |  |  |  |  |  |
|  |  |  |  |  |  |  |  |  |  |  |  |  | 806.38 (H1N2-PROC) |  |  |  |  |  |
|  |  |  |  |  |  |  |  |  |  |  |  |  | 968.88 (H2N2-PROC) |  |  |  |  |  |
|  |  |  |  |  |  |  |  |  |  |  |  |  | 1131.69 (H3N2-PROC) |  |  |  |  |  |
| J | 5.26 | 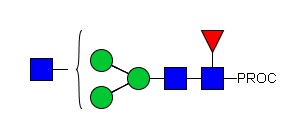 | 3 | 3 | 1 | 0 | 1479.65 | 740.32 | 493.89 | 1479.76 | 740.42 | n.d. | 441.25 (N1-PROC) | 1130.64 (H3N2-PROC) |  |  |  |  |
|  |  |  |  |  |  |  |  |  |  |  |  |  | 587.50 (N1F1-PROC) | 1276.71 (H3N2F1-PROC) |  |  |  |  |
|  |  |  |  |  |  |  |  |  |  |  |  |  | 644.25 (N2-PROC) | 366.01 (H1N1) |  |  |  |  |
|  |  |  |  |  |  |  |  |  |  |  |  |  | 790.38 (N2F1-PROC) | 893.29 (H3N2) |  |  |  |  |
|  |  |  |  |  |  |  |  |  |  |  |  |  | 969.63 (H2N2-PROC) |  |  |  |  |  |
| K | 5.59 | 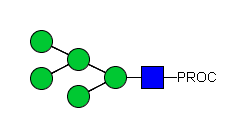 | 5 | 1 | 0 | 0 | 1251.53 | 626.27 | 417.85 | 1251.69 | 626.35 | n.d. | 441.33 (N1-PROC) |  |  |  |  |  |
|  |  |  |  |  |  |  |  |  |  |  |  |  | 603.38 (H1N1-PROC) |  |  |  |  |  |
|  |  |  |  |  |  |  |  |  |  |  |  |  | 765.46 (H2N1-PROC) |  |  |  |  |  |
|  |  |  |  |  |  |  |  |  |  |  |  |  | 927.63 (H3N1-PROC) |  |  |  |  |  |
|  |  |  |  |  |  |  |  |  |  |  |  |  |  |  |  |  |  |  |
| L | 5.79 | 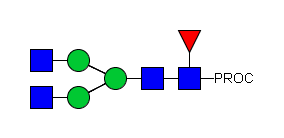 | 3 | 4 | 1 | 0 | 1682.72 | 841.87 | 561.58 | n.d. | 841.97 | n.d. | 441.26 (N1-PROC) | 1317.79 (H2N3F1-PROC) |  |  |  |  |
|  |  |  |  |  |  |  |  |  |  |  |  |  | 587.38 (N1F1-PROC) | 1333.63 (H3N3-PROC) |  |  |  |  |
|  |  |  |  |  |  |  |  |  |  |  |  |  | 645.50 (N2-PROC) | 1479.69 (H3N3F1-PROC) |  |  |  |  |
|  |  |  |  |  |  |  |  |  |  |  |  |  | 968.50 (H2N2-PROC) | 366.00 (H1N1) |  |  |  |  |
|  |  |  |  |  |  |  |  |  |  |  |  |  | 1276.63 (H3N2F1-PROC) | 689.38 (H3N1) |  |  |  |  |
| M | 6.04 | 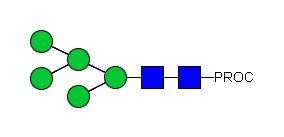 | 5 | 2 | 0 | 0 | 1454.61 | 727.81 | 485.54 | 1454.77 | 727.92 | n.d. | 441.34 (N1-PROC) | 366.23 (H1N1) |  |  |  |  |
|  |  |  |  |  |  |  |  |  |  |  |  |  | 644.39 (N2-PROC) | 528.20 (H2N1) |  |  |  |  |
|  |  |  |  |  |  |  |  |  |  |  |  |  | 806.49 (H1N2-PROC) | 690.28 (H3N1) |  |  |  |  |
|  |  |  |  |  |  |  |  |  |  |  |  |  | 968.61 (H2N2-PROC) | 852.37 (H4N1) |  |  |  |  |
|  |  |  |  |  |  |  |  |  |  |  |  |  | 1131.63 (H3N2-PROC) | 1014.46 (H5N1) |  |  |  |  |
| N | 6.21 | - | 4 | 4 | 0 | 1 | 1989.82 | 995.41 | 663.94 | n.d. | 995.20 | 663.80 |  |  |  |  |  |  |
|  |  |  |  |  |  |  |  |  |  |  |  |  |  |  |  |  |  |  |
|  |  |  |  |  |  |  |  |  |  |  |  |  | No MS/MS data detected |  |  |  |  |  |
|  |  |  |  |  |  |  |  |  |  |  |  |  |  |  |  |  |  |  |
|  |  |  |  |  |  |  |  |  |  |  |  |  |  |  |  |  |  |  |
| O | 6.45 | 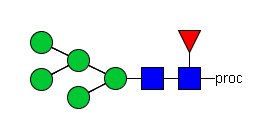 | 5 | 2 | 1 | 0 | 1600.67 | 800.84 | 534.23 | n.d. | 800.92 | n.d. | 441.32 (N1-PROC) | 1114.63 (H2N2F1-PROC) | 852.38 (H4N1) |  |  |  |
|  |  |  |  |  |  |  |  |  |  |  |  |  | 587.46 (N1F1-PROC) | 1130.75 (H3N2) |  |  |  |  |
|  |  |  |  |  |  |  |  |  |  |  |  |  | 644.28 (N2-PROC) | 366.25 (H1N1) |  |  |  |  |
|  |  |  |  |  |  |  |  |  |  |  |  |  | 790.38 (N2F1-PROC) | 528.25 (H2N1) |  |  |  |  |
|  |  |  |  |  |  |  |  |  |  |  |  |  | 968.63 (H2N2-PROC) | 690.38 (H3N1) |  |  |  |  |
| P | 6.81 | 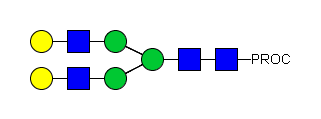 | 5 | 4 | 0 | 0 | 1860.77 | 930.89 | 620.93 | n.d. | 931.07 | n.d. | 441.50 (N1-PROC) | 1495.93 (H4N3-PROC) | 1055.25 (H4N2) |  |  |  |
|  |  |  |  |  |  |  |  |  |  |  |  |  | 644.52 (N2-PROC) | 1657.76 (H4N3-PROC) | 1420.75 (H5N3) |  |  |  |
|  |  |  |  |  |  |  |  |  |  |  |  |  | 806.38 (H1N2-PROC) | 366.19 (H1N1) |  |  |  |  |
|  |  |  |  |  |  |  |  |  |  |  |  |  | 1130.63 (H3N2-PROC) | 528.13 (H2N1) |  |  |  |  |
|  |  |  |  |  |  |  |  |  |  |  |  |  | 1334.00 (H3N3-PROC) | 731.25 (H2N2) |  |  |  |  |
| Q | 6.91 | 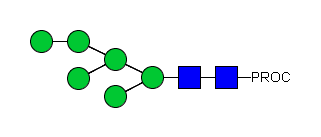 | 6 | 2 | 0 | 0 | 1616.67 | 808.84 | 539.56 | n.d. | 808.94 | n.d. | 441.34 (N1-PROC) | 690.28 (H3N1) |  |  |  |  |
|  |  |  |  |  |  |  |  |  |  |  |  |  | 644.39 (N2-PROC) | 852.38 (H4N1) |  |  |  |  |
|  |  |  |  |  |  |  |  |  |  |  |  |  | 968.63 (H2N2-PROC) | 1014.43 (H5N1) |  |  |  |  |
|  |  |  |  |  |  |  |  |  |  |  |  |  | 366.19 (H1N1) | 1176.54 (H6N1) |  |  |  |  |
|  |  |  |  |  |  |  |  |  |  |  |  |  | 528.27 (H2N1) |  |  |  |  |  |
| R | 7.26 | 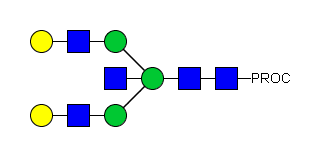 | 5 | 5 | 0 | 0 | 2063.85 | 1032.43 | 688.62 | n.d. | 1032.57 | 688.71 | 441.38 (N1-PROC) | 1130.68 (H3N2-PROC) | 569.13 (H1N2) |  |  |  |
|  |  |  |  |  |  |  |  |  |  |  |  |  | 644.88 (N2-PROC) | 1171.66 (H2N3-PROC) |  |  |  |  |
|  |  |  |  |  |  |  |  |  |  |  |  |  | 806.50 (H1N2-PROC) | 1333.82 (H3N3-PROC) |  |  |  |  |
|  |  |  |  |  |  |  |  |  |  |  |  |  | 968.50 (H2N2-PROC) | 1495.75 (H4N3-PROC) |  |  |  |  |
|  |  |  |  |  |  |  |  |  |  |  |  |  | 1009.55 (H1N3-PROC) | 366.17 (H1N1) |  |  |  |  |
| R | 7.26 | 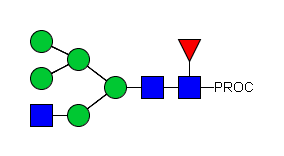 | 5 | 3 | 1 | 0 | 1803.75 | 902.38 | 601.92 | n.d. | 902.44 | n.d. | 441.25 (N1-PROC) | 1438.85 (H4N2F1-PROC) |  |  |  |  |
|  |  |  |  |  |  |  |  |  |  |  |  |  | 644.38 (N2-PROC) | 366.13 (H1N1) |  |  |  |  |
|  |  |  |  |  |  |  |  |  |  |  |  |  | 968.38 (H2N2-PROC) | 690.75 (H3N1) |  |  |  |  |
|  |  |  |  |  |  |  |  |  |  |  |  |  | 1130.71 (H3N2-PROC) |  |  |  |  |  |
|  |  |  |  |  |  |  |  |  |  |  |  |  | 1292.69 (H4N2-PROC) |  |  |  |  |  |
| S | 7.44 | 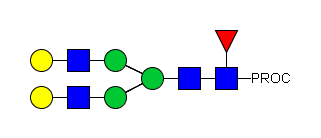 | 5 | 4 | 1 | 0 | 2006.83 | 1003.92 | 669.62 | n.d. | 1004.01 |  | 441.38 (N1-PROC) | 1495.63 (H4N3-PROC) |  |  |  |  |
|  |  |  |  |  |  |  |  |  |  |  |  |  | 587.38 (N1F1-PROC) | 1641.82 (H4N3F1-PROC) |  |  |  |  |
|  |  |  |  |  |  |  |  |  |  |  |  |  | 952.13 (H1N2F1-PROC) | 366.13 (H1N1) |  |  |  |  |
|  |  |  |  |  |  |  |  |  |  |  |  |  | 968.88 (H2N2-PROC) | 1055.50 (H4N2) |  |  |  |  |
|  |  |  |  |  |  |  |  |  |  |  |  |  | 1131.50 (H3N2-PROC) |  |  |  |  |  |
| T | 7.61 | 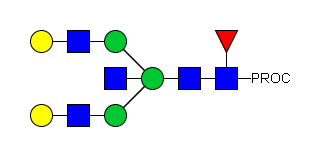 | 5 | 5 | 1 | 0 | 2209.91 | 1105.46 | 737.31 | n.d. | 1105.59 | 737.39 | 441.48 (N1-PROC) | 1009.50 (H1N3-PROC) | 366.17 (H1N1) |  |  |  |
|  |  |  |  |  |  |  |  |  |  |  |  |  | 587.63 (N1F1-PROC) | 1171.75 (H2N3-PROC) | 528.43 (H2N1) |  |  |  |
|  |  |  |  |  |  |  |  |  |  |  |  |  | 644.13 (N2-PROC) | 1317.75 (H2N3F1-PROC) | 1259.63 (H4N3) |  |  |  |
|  |  |  |  |  |  |  |  |  |  |  |  |  | 790.50 (N2F1-PROC) | 1333.75 (H3N3-PROC) |  |  |  |  |
|  |  |  |  |  |  |  |  |  |  |  |  |  | 968.50 (H2N2-PROC) | 1480.82 (H3N3F1-PROC) |  |  |  |  |
| U | 7.78 | 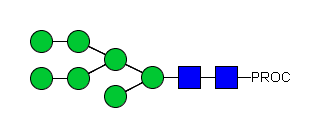 | 7 | 2 | 0 | 0 | 1778.72 | 889.86 | 593.58 | n.d. | 889.96 | n.d. | 441.34 (N1-PROC) | 366.16 (H1N1) | 1176.53 (H6N1) |  |  |  |
|  |  |  |  |  |  |  |  |  |  |  |  |  | 644.42 (N2-PROC) | 528.20 (H2N1) | 1338.63 (H7N1) |  |  |  |
|  |  |  |  |  |  |  |  |  |  |  |  |  | 806.46 (H1N2-PROC) | 690.21 (H3N1) |  |  |  |  |
|  |  |  |  |  |  |  |  |  |  |  |  |  | 968.53 (H2N2-PROC) | 852.37 (H4N1) |  |  |  |  |
|  |  |  |  |  |  |  |  |  |  |  |  |  | 1130.67 (H3N2-PROC) | 1014.41 (H5N1) |  |  |  |  |
| V | 7.82 |  | 7 | 2 | 0 | 0 | 1778.72 | 889.86 | 593.58 | n.d. | 889.97 | n.d. | 441.34 (N1-PROC) | 366.13 (H1N1) | 1176.59 (H6N1) |  |  |  |
|  |  |  |  |  |  |  |  |  |  |  |  |  | 644.45 (N2-PROC) | 528.20 (H2N1) | 1338.60 (H7N1) |  |  |  |
|  |  |  |  |  |  |  |  |  |  |  |  |  | 806.54 (H1N2-PROC) | 690.27 (H3N1) |  |  |  |  |
|  |  |  |  |  |  |  |  |  |  |  |  |  | 968.82 (H2N2-PROC) | 852.43 (H4N1) |  |  |  |  |
|  |  |  |  |  |  |  |  |  |  |  |  |  | 1131.63 (H3N2-PROC) | 1014.53 (H5N1) |  |  |  |  |
| W | 8.03 |  | 5 | 5 | 0 | 1 | 2501.01 | 1251.01 | 834.34 | n.d. | n.d. | 833.85 | 1333.85 (H3N3-PROC) | 657.25 (H1N1S1) |  |  |  |  |
|  |  |  |  |  |  |  |  |  |  |  |  |  | 1479.68 (H3N3F1-PROC) | 1258.63 (H4N3) |  |  |  |  |
|  |  |  |  |  |  |  |  |  |  |  |  |  | 1787.13 (H4N3S1-PROC) |  |  |  |  |  |
|  |  |  |  |  |  |  |  |  |  |  |  |  | 366.20 (H1N1) |  |  |  |  |  |
|  |  |  |  |  |  |  |  |  |  |  |  |  | 528.38 (H2N1) |  |  |  |  |  |
| W | 8.03 |  | 5 | 5 | 2 | 0 | 2355.97 | 1178.49 | 785.99 | n.d. | 1178.59 | 786.10 | 441.45 (N1-PROC) | 1171.88 (H2N3-PROC) | 512.32 (H1N1F1) | 1258.50 (H4N3) |  |  |
|  |  |  |  |  |  |  |  |  |  |  |  |  | 587.38 (N1F1-PROC) | 1317.75 (H2N3F1-PROC) | 528.17 (H2N1) |  |  |  |
|  |  |  |  |  |  |  |  |  |  |  |  |  | 645.50 (N2-PROC) | 1333.63 (H3N3-PROC) | 690.00 (H3N1) |  |  |  |
|  |  |  |  |  |  |  |  |  |  |  |  |  | 806.38 (H1N2-PROC) | 1479.81 (H3N3F1-PROC) | 852.51 (H4N1) |  |  |  |
|  |  |  |  |  |  |  |  |  |  |  |  |  | 1155.75 (H1N3F1-PROC) | 366.18 (H1N1) | 893.38 (H3N2) |  |  |  |
| X | 8.18 | - | 6 | 5 | 0 | 1 | 2517.00 | 1259.00 | 839.67 | n.d. | n.d. | 839.88 |  |  |  |  |  |  |
|  |  |  |  |  |  |  |  |  |  |  |  |  |  |  |  |  |  |  |
|  |  |  |  |  |  |  |  |  |  |  |  |  | No MS/MS data detected |  |  |  |  |  |
|  |  |  |  |  |  |  |  |  |  |  |  |  |  |  |  |  |  |  |
|  |  |  |  |  |  |  |  |  |  |  |  |  |  |  |  |  |  |  |
| Y | 8.35 |  | 5 | 5 | 2 | 0 | 2355.97 | 1178.49 | 785.99 | n.d. | 1179.09 | 786.12 | 441.33 (N1-PROC) | 1114.50 (H2N2F1-PROC) | 1479.81 (H3N3F1-PROC) | 893.38 (H3N2) |  |  |
|  |  |  |  |  |  |  |  |  |  |  |  |  | 587.49 (N1F1-PROC) | 1131.38 (H3N2-PROC) | 1495.88 (H4N3-PROC) | 1258.63 (H4N3) |  |  |
|  |  |  |  |  |  |  |  |  |  |  |  |  | 644.56 (N2-PROC) | 1155.88 (H1N3F1-PROC) | 366.22 (H1N1) |  |  |  |
|  |  |  |  |  |  |  |  |  |  |  |  |  | 968.75 (H2N2-PROC) | 1317.51 (H2N3F1-PROC) | 512.33 (H1N1F1) |  |  |  |
|  |  |  |  |  |  |  |  |  |  |  |  |  | 1009.50 (H1N3-PROC) | 1333.82 (H3N3-PROC) | 528.50 (H2N1) |  |  |  |
| Z | 8.46 |  | 3 | 6 | 3 | 0 | 2380.99 | 1191.00 | 794.34 | n.d. | 1191.12 | 794.44 | 441.50 (N1-PROC) | 1130.69 (H3N2-PROC) | 1464.00 (H2N3F2-PROC) | 731.88 (H2N2) |  |  |
|  |  |  |  |  |  |  |  |  |  |  |  |  | 587.28 (N1F1-PROC) | 1171.64 (H2N3-PROC) | 1479.92 (H3N3F1-PROC) | 1242.63 (H3N3F1) |  |  |
|  |  |  |  |  |  |  |  |  |  |  |  |  | 644.25 (N2-PROC) | 1276.87 (H3N2F1-PROC) | 1626.00 (H3N3F2-PROC) | 1388.75 (H3N3F2) |  |  |
|  |  |  |  |  |  |  |  |  |  |  |  |  | 806.50 (H1N2-PROC) | 1317.63 (H2N3F1-PROC) | 366.13 (H1N1) | 1445.75 (H3N4F1) |  |  |
|  |  |  |  |  |  |  |  |  |  |  |  |  | 968.53 (H2N2-PROC) | 1333.75 (H3N3-PROC) | 690.25 (H3N1) |  |  |  |
| AA | 8.69 |  | 8 | 2 | 0 | 0 | 1940.77 | 970.89 | 647.60 | n.d. | 970.99 | n.d. | 441.33 (N1-PROC) | 1455.88 (H5N2-PROC) | 1014.36 (H5N1) |  |  |  |
|  |  |  |  |  |  |  |  |  |  |  |  |  | 644.40 (N2-PROC) | 366.25 (H1N1) | 1176.62 (H6N1) |  |  |  |
|  |  |  |  |  |  |  |  |  |  |  |  |  | 806.54 (H1N2-PROC) | 528.29 (H2N1) | 1338.75 (H7N1) |  |  |  |
|  |  |  |  |  |  |  |  |  |  |  |  |  | 1130.60 (H3N2-PROC) | 690.22 (H3N1) | 1500.61 (H8N1) |  |  |  |
|  |  |  |  |  |  |  |  |  |  |  |  |  | 1292.70 (H4N2-PROC) | 852.38 (H4N1) |  |  |  |  |
| AB | 8.99 |  | 5 | 4 | 3 | 0 | 2298.95 | 1149.98 | 766.99 | n.d. | 1150.05 | 767.33 | 587.25 (N1F1-PROC) | 366.16 (H1N1) | 1201.63 (H4N2F1) |  |  |  |
|  |  |  |  |  |  |  |  |  |  |  |  |  | 968.00 (H2N2-PROC) | 512.29 (H1N1F1) | 1347.63 (H4N2F2) |  |  |  |
|  |  |  |  |  |  |  |  |  |  |  |  |  | 1114.63 (H2N2F1-PROC) | 528.25 (H2N1) | 1363.75 (H5N2F1) |  |  |  |
|  |  |  |  |  |  |  |  |  |  |  |  |  | 1130.75 (H3N2-PROC) | 674.50 (H2N1F1) |  |  |  |  |
|  |  |  |  |  |  |  |  |  |  |  |  |  | 1276.74 (H3N2F1-PROC) | 1185.63 (H3N2F2) |  |  |  |  |
| AC | 9.09 |  | 5 | 4 | 2 | 1 | 2243.98 | 1222.50 | 815.33 | n.d. | n.d. | 815.14 | 806.25 (H1N2-PROC) | 657.22 (H1N1S1) | 1711.76 (H5N3S1) |  |  |  |
|  |  |  |  |  |  |  |  |  |  |  |  |  | 968.48 (H2N2-PROC) | 981.38 (H3N1S1) |  |  |  |  |
|  |  |  |  |  |  |  |  |  |  |  |  |  | 1114.75 (H2N2F1-PROC) | 998.75 (H4N1F1) |  |  |  |  |
|  |  |  |  |  |  |  |  |  |  |  |  |  | 1130.71 (H3N2-PROC) | 1096.50 (H3N3) |  |  |  |  |
|  |  |  |  |  |  |  |  |  |  |  |  |  | 366.26 (H1N1) | 1346.50 (H4N2S1) |  |  |  |  |
| AD | 9.38 |  | 9 | 2 | 0 | 0 | 2102.83 | 1051.92 | 701.61 | n.d. | 1052.02 | n.d. | 441.29 (N1-PROC) | 1292.75 (H4N2-PROC) | 690.40 (H3N1) | 1500.65 (H8N1) |  |  |
|  |  |  |  |  |  |  |  |  |  |  |  |  | 644.34 (N2-PROC) | 1455.75 (H5N2-PROC) | 852.50 (H4N1) | 1662.75 (H9N1) |  |  |
|  |  |  |  |  |  |  |  |  |  |  |  |  | 806.53 (H1N2-PROC) | 1617.63 (H6N2-PROC) | 1014.50 (H5N1) |  |  |  |
|  |  |  |  |  |  |  |  |  |  |  |  |  | 968.38 (H2N2-PROC) | 366.13 (H1N1) | 1176.47 (H6N1) |  |  |  |
|  |  |  |  |  |  |  |  |  |  |  |  |  | 1130.76 (H3N2-PROC) | 528.14 (H2N1) | 1338.88 (H7N1) |  |  |  |

**Table E.** Structural characterization of procainamide labelled HT29 human colorectal cancer cell line *N-*glycans from experiment 1. Structures for *N-*glycans are depicted following the Consortium for Functional Glycomics (CFG) notation: *N*-acetylglucosamine (N; blue square), fucose (F; red triangle), galactose (H; yellow circle), mannose (H; green circle), *N*-acetylneuraminic acid (S; purple diamond). Glycan compositions are given in the terms of hexose (H), *N*-acetylhexosamine (N), deoxyhexose (F), *N*-acetylneuraminic acid (S). *^,^ **^,^ ***^,^ ****No MS data detected.

**HT29**

15

20

25

30

35

40

45

50

**Time [min]**

**A**

**B**

**C**

**D**

**E**

**F**

**G**

**H**

**I**

**J**

**K**

**L**

**M**

**N**

**O**

**P**

**Q**

**R**

**S**

**T**

**U**

**V**

|  | |  |  | | | |  | | | | | | | | | | | |
| --- | --- | --- | --- | --- | --- | --- | --- | --- | --- | --- | --- | --- | --- | --- | --- | --- | --- | --- |
|  | |  |  | | | |  | | | | | | | | | | | |
| Average | | Possible structure | Composition | | | | HT29 LC-ESI-MS (in-solution PNGaseF release) | | | | | | | | | | | |
| Peak ID | GU (Procainamide) |  |  |  |  |  | [M/Z]^+^ calculated | [M/Z]^2+^ calculated | [M/Z]^3+^ calculated | [M/Z]^+^ registered | [M/Z]^2+^ registered | [M/Z]^3+^ registered | [M/Z] characteristic fragment ions (composition) | | | | | |
|  |  |  | Hex (H) | HexNAc (N) | Fuc (F) | Neu5Ac (S) |  |  |  |  |  |  |  |  |  |  |  |  |
| A | 3.61 |  | 3 | 1 | 0 | 0 | 927.43 | 464.22 | 309.81 | 927.41 | 464.22 | n.d. | 441.17 (N1-PROC) |  |  |  |  |  |
|  |  |  |  |  |  |  |  |  |  |  |  |  | 603.34 (H1N1-PROC) |  |  |  |  |  |
|  |  |  |  |  |  |  |  |  |  |  |  |  | 765.37 (H2N1-PROC) |  |  |  |  |  |
|  |  |  |  |  |  |  |  |  |  |  |  |  |  |  |  |  |  |  |
|  |  |  |  |  |  |  |  |  |  |  |  |  |  |  |  |  |  |  |
| B | 3.72 |  | 2 | 2 | 1 | 0 | 1114.51 | 557.76 | 372.18 | 1114.54 | 557.77 | 372.25 | 441.14 (N1-PROC) |  |  |  |  |  |
|  |  |  |  |  |  |  |  |  |  |  |  |  | 587.30 (N1F1-PROC) |  |  |  |  |  |
|  |  |  |  |  |  |  |  |  |  |  |  |  | 644.38 (N2-PROC) |  |  |  |  |  |
|  |  |  |  |  |  |  |  |  |  |  |  |  | 806.41 (H1N2-PROC) |  |  |  |  |  |
|  |  |  |  |  |  |  |  |  |  |  |  |  | 968.42 (H2N2-PROC) |  |  |  |  |  |
| C | 3.73 |  | 3 | 1 | 0 | 0 | 927.43 | 464.22 | 309.81 | 927.43 | 464.25 | n.d. | 441.19 (N1-PROC) |  |  |  |  |  |
|  |  |  |  |  |  |  |  |  |  |  |  |  | 603.35 (H1N1-PROC) |  |  |  |  |  |
|  |  |  |  |  |  |  |  |  |  |  |  |  | 765.41 (H2N1-PROC) |  |  |  |  |  |
|  |  |  |  |  |  |  |  |  |  |  |  |  |  |  |  |  |  |  |
|  |  |  |  |  |  |  |  |  |  |  |  |  |  |  |  |  |  |  |
| D | 4.24 |  | 3 | 2 | 0 | 0 | 1130.51 | 565.76 | 377.51 | 1130.48 | 565.78 | n.d. | 441.18 (N1-PROC) |  |  |  |  |  |
|  |  |  |  |  |  |  |  |  |  |  |  |  | 644.35 (N2-PROC) |  |  |  |  |  |
|  |  |  |  |  |  |  |  |  |  |  |  |  | 806.45 (H1N2-PROC) |  |  |  |  |  |
|  |  |  |  |  |  |  |  |  |  |  |  |  | 968.45 (H2N2-PROC) |  |  |  |  |  |
|  |  |  |  |  |  |  |  |  |  |  |  |  | 690.25 (H3N1) |  |  |  |  |  |
| E | 4.55 |  | 4 | 1 | 0 | 0 | 1089.48 | 545.24 | 363.83 | 1089.46 | 545.24 | n.d. | 441.22 (N1-PROC) |  |  |  |  |  |
|  |  |  |  |  |  |  |  |  |  |  |  |  | 603.30 (H1N1-PROC) |  |  |  |  |  |
|  |  |  |  |  |  |  |  |  |  |  |  |  | 765.40 (H2N1-PROC) |  |  |  |  |  |
|  |  |  |  |  |  |  |  |  |  |  |  |  | 927.40 (H3N1-PROC) |  |  |  |  |  |
|  |  |  |  |  |  |  |  |  |  |  |  |  |  |  |  |  |  |  |
| F | 4.66 | - | 3 | 2 | 1 | 0 | 1276.57 | 638.79 | 426.19 | 1276.56 | 638.79 | n.d. |  |  |  |  |  |  |
|  |  |  |  |  |  |  |  |  |  |  |  |  |  |  |  |  |  |  |
|  |  |  |  |  |  |  |  |  |  |  |  |  | No MS/MS data detected |  |  |  |  |  |
|  |  |  |  |  |  |  |  |  |  |  |  |  |  |  |  |  |  |  |
|  |  |  |  |  |  |  |  |  |  |  |  |  |  |  |  |  |  |  |
| F | 4.66 |  | 4 | 1 | 0 | 0 | 1089.48 | 545.24 | 363.83 | 1089.46 | 545.23 | n.d. | 441.19 (N1-PROC) |  |  |  |  |  |
|  |  |  |  |  |  |  |  |  |  |  |  |  | 603.28 (H1N1-PROC) |  |  |  |  |  |
|  |  |  |  |  |  |  |  |  |  |  |  |  | 765.42 (H2N1-PROC) |  |  |  |  |  |
|  |  |  |  |  |  |  |  |  |  |  |  |  | 927.38 (H3N1-PROC) |  |  |  |  |  |
|  |  |  |  |  |  |  |  |  |  |  |  |  |  |  |  |  |  |  |
| G | 5.57 |  | 5 | 1 | 0 | 0 | 1251.53 | 626.27 | 417.85 | 1251.52 | 626.26 | n.d. | 441.29 (N1-PROC) |  |  |  |  |  |
|  |  |  |  |  |  |  |  |  |  |  |  |  | 603.33 (H1N1-PROC) |  |  |  |  |  |
|  |  |  |  |  |  |  |  |  |  |  |  |  | 765.41 (H2N1-PROC) |  |  |  |  |  |
|  |  |  |  |  |  |  |  |  |  |  |  |  | 927.41 (H3N1-PROC) |  |  |  |  |  |
|  |  |  |  |  |  |  |  |  |  |  |  |  | 1089.45 (H4N1-PROC) |  |  |  |  |  |
| H | 5.88 |  | 3 | 3 | 1 | 0 | 1479.65 | 740.33 | 493.89 | n.d. | 740.32 | n.d. | 1114.38 (H2N2F1-PROC) |  |  |  |  |  |
|  |  |  |  |  |  |  |  |  |  |  |  |  | 1130.25 (H3N2-PROC) |  |  |  |  |  |
|  |  |  |  |  |  |  |  |  |  |  |  |  | 1276.56 (H3N2F1-PROC) |  |  |  |  |  |
|  |  |  |  |  |  |  |  |  |  |  |  |  | 731.50 (H2N2) |  |  |  |  |  |
|  |  |  |  |  |  |  |  |  |  |  |  |  |  |  |  |  |  |  |
| I | 6.09 |  | 5 | 2 | 0 | 0 | 1454.61 | 727.81 | 485.54 | 1454.60 | 727.80 | n.d. | 441.22 (N1-PROC) | 690.22 (H3N1) |  |  |  |  |
|  |  |  |  |  |  |  |  |  |  |  |  |  | 644.38 (N2-PROC) | 852.29 (H4N1) |  |  |  |  |
|  |  |  |  |  |  |  |  |  |  |  |  |  | 807.38 (H1N2-PROC) | 1014.33 (H5N1) |  |  |  |  |
|  |  |  |  |  |  |  |  |  |  |  |  |  | 366.00 (H1N1) |  |  |  |  |  |
|  |  |  |  |  |  |  |  |  |  |  |  |  | 528.13 (H2N1) |  |  |  |  |  |
| I | 6.09 |  | 4 | 3 | 1 | 0 | 1641.69 | 821.35 | 547.90 | n.d. | 821.78 | n.d. | 587.37 (N1F1-PROC) |  |  |  |  |  |
|  |  |  |  |  |  |  |  |  |  |  |  |  | 968.50 (H2N2-PROC) | 690.88 (H3N1) |  |  |  |  |
|  |  |  |  |  |  |  |  |  |  |  |  |  | 1114.64 (H2N2F1-PROC) | 1055.36 (H4N2) |  |  |  |  |
|  |  |  |  |  |  |  |  |  |  |  |  |  | 1130.63 (H3N2-PROC) |  |  |  |  |  |
|  |  |  |  |  |  |  |  |  |  |  |  |  | 1276.56 (H3N2F1-PROC) |  |  |  |  |  |
| J | 6.21 |  | 6 | 1 | 0 | 0 | 1413.59 | 707.29 | 471.87 | n.d. | 707.28 | n.d. | 441.25 (N1-PROC) |  |  |  |  |  |
|  |  |  |  |  |  |  |  |  |  |  |  |  | 603.63 (H1N1-PROC) |  |  |  |  |  |
|  |  |  |  |  |  |  |  |  |  |  |  |  | 765.38 (H2N1-PROC) |  |  |  |  |  |
|  |  |  |  |  |  |  |  |  |  |  |  |  | 927.28 (H3N1-PROC) |  |  |  |  |  |
|  |  |  |  |  |  |  |  |  |  |  |  |  | 690.75 (H3N1) |  |  |  |  |  |
| K | 6.41 |  | 4 | 3 | 1 | 0 | 1641.69 | 821.35 | 547.90 | n.d. | 821.31 | 547.92 | 441.28 (N1-PROC) | 1317.38 (H2N2F1-PROC) |  |  |  |  |
|  |  |  |  |  |  |  |  |  |  |  |  |  | 644.25 (N2-PROC) | 365.99 (H1N1) |  |  |  |  |
|  |  |  |  |  |  |  |  |  |  |  |  |  | 968.37 (H2N2-PROC) | 512.20 (H1N1F1) |  |  |  |  |
|  |  |  |  |  |  |  |  |  |  |  |  |  | 1130.50 (H3N2-PROC) | 527.50 (H2N1) |  |  |  |  |
|  |  |  |  |  |  |  |  |  |  |  |  |  | 1276.63 (H3N2F1-PROC) | 1055.75 (H4N2) |  |  |  |  |
| L | 6.81 |  | 6 | 2 | 0 | 0 | 1616.67 | 808.84 | 539.56 | n.d. | 808.82 | n.d. | 441.23 (N1-PROC) | 690.27 (H3N1) |  |  |  |  |
|  |  |  |  |  |  |  |  |  |  |  |  |  | 644.25 (N2-PROC) | 852.28 (H4N1) |  |  |  |  |
|  |  |  |  |  |  |  |  |  |  |  |  |  | 968.38 (H2N2-PROC) | 1014.35 (H5N1) |  |  |  |  |
|  |  |  |  |  |  |  |  |  |  |  |  |  | 366.01 (H1N1) | 1176.34 (H6N1) |  |  |  |  |
|  |  |  |  |  |  |  |  |  |  |  |  |  | 528.07 (H2N1) |  |  |  |  |  |
| M | 7.11 |  | 7 | 1 | 0 | 0 | 1575.64 | 788.32 | 525.88 | n.d. | 788.29 | 525.40 | 441.23 (N1-PROC) | 366.63 (H1N1) |  |  |  |  |
|  |  |  |  |  |  |  |  |  |  |  |  |  | 603.38 (H1N1-PROC) | 690.50 (H3N1) |  |  |  |  |
|  |  |  |  |  |  |  |  |  |  |  |  |  | 765.25 (H2N1-PROC) |  |  |  |  |  |
|  |  |  |  |  |  |  |  |  |  |  |  |  | 927.47 (H3N1-PROC) |  |  |  |  |  |
|  |  |  |  |  |  |  |  |  |  |  |  |  | 1089.50 (H4N1-PROC) |  |  |  |  |  |
| N | 7.41 |  | 6 | 3 | 0 | 0 | 1819.75 | 910.38 | 607.25 | n.d. | 910.36 | 607.62 | 441.35 (N1-PROC) | 1292.63 (H4N2-PROC) | 1379.88 (H6N2) |  |  |  |
|  |  |  |  |  |  |  |  |  |  |  |  |  | 644.38 (N2-PROC) | 1454.58 (H5N2-PROC) |  |  |  |  |
|  |  |  |  |  |  |  |  |  |  |  |  |  | 806.25 (H1N2-PROC) | 366.02 (H1N1) |  |  |  |  |
|  |  |  |  |  |  |  |  |  |  |  |  |  | 968.63 (H2N2-PROC) | 528.25 (H2N1) |  |  |  |  |
|  |  |  |  |  |  |  |  |  |  |  |  |  | 1130.63 (H3N2-PROC) | 690.14 (H3N1) |  |  |  |  |
| O | 7.42 |  | 5 | 5 | 1 | 0 | 2209.91 | 1105.46 | 737.31 | n.d. | 1105.91 | 737.29 | 441.25 (N1-PROC) | 366.00 (H1N1) |  |  |  |  |
|  |  |  |  |  |  |  |  |  |  |  |  |  | 587.19 (N1F1-PROC) | 528.25 (H2N1) |  |  |  |  |
|  |  |  |  |  |  |  |  |  |  |  |  |  | 1114.51 (H2N2F1-PROC) | 690.88 (H3N1) |  |  |  |  |
|  |  |  |  |  |  |  |  |  |  |  |  |  | 1317.50 (H2N3F1-PROC) | 1258.55 (H4N3) |  |  |  |  |
|  |  |  |  |  |  |  |  |  |  |  |  |  | 1479.65 (H3N3F1-PROC) |  |  |  |  |  |
| P | 7.82 |  | 7 | 2 | 0 | 0 | 1778.72 | 889.86 | 593.58 | n.d. | 889.83 | n.d. | 441.22 (N1-PROC) | 366.00 (H1N1) | 1176.34 (H6N1) |  |  |  |
|  |  |  |  |  |  |  |  |  |  |  |  |  | 644.36 (N2-PROC) | 528.12 (H2N1) |  |  |  |  |
|  |  |  |  |  |  |  |  |  |  |  |  |  | 806.35 (H1N2-PROC) | 690.23 (H3N1) |  |  |  |  |
|  |  |  |  |  |  |  |  |  |  |  |  |  | 968.52 (H2N2-PROC) | 852.13 (H4N1) |  |  |  |  |
|  |  |  |  |  |  |  |  |  |  |  |  |  | 1130.59 (H2N2-PROC) | 1014.34 (H5N1) |  |  |  |  |
| Q | 8.12 |  | 5 | 2 | 0 | 2 | 2442.96 | 1221.99 | 814.99 | n.d. | n.d. | 814.93 | 441.09 (N1-PROC) | 1333.50 (H3N3-PROC) | 657.22 (H1N1S1) | 1346.63 (H4N2S1) |  |  |
|  |  |  |  |  |  |  |  |  |  |  |  |  | 644.13 (N2-PROC) | 1495.75 (H4N3-PROC) | 690.63 (H3N1) |  |  |  |
|  |  |  |  |  |  |  |  |  |  |  |  |  | 806.25 (H1N2-PROC) | 1787.75 (H4N3S1-PROC) | 730.75 (H2N2) |  |  |  |
|  |  |  |  |  |  |  |  |  |  |  |  |  | 968.31 (H2N2-PROC) | 366.01 (H1N1) | 893.13 (H3N2) |  |  |  |
|  |  |  |  |  |  |  |  |  |  |  |  |  | 1130.58 (H3N2-PROC) | 528.25 (H2N1) | 1055.25 (H4N2) |  |  |  |
| R | 8.52 |  | 4 | 5 | 3 | 0 | 2339.97 | 1170.49 | 780.66 | n.d. | 1170.44 | 780.64 | 441.25 (N1-PROC) | 1114.38 (H2N2F1-PROC) | 365.90 (H1N1) | 877.25 (H2N2F1) |  |  |
|  |  |  |  |  |  |  |  |  |  |  |  |  | 587.50 (N1F1-PROC) | 1276.75 (H3N2F1-PROC) | 512.16 (H1N1F1) | 893.38 (H3N2) |  |  |
|  |  |  |  |  |  |  |  |  |  |  |  |  | 644.58 (N2-PROC) | 1479.59 (H3N3F1-PROC) | 569.14 (H1N2) | 1258.75 (H4N3) |  |  |
|  |  |  |  |  |  |  |  |  |  |  |  |  | 806.50 (H1N2-PROC) | 1624.63 (H3N3F2-PROC) | 658.38 (H1N1F2) | 1404.50 (H4N3F1) |  |  |
|  |  |  |  |  |  |  |  |  |  |  |  |  | 968.75 (H2N2-PROC) | 1771.63 (H3N3F3-PROC) | 731.98 (H2N2) |  |  |  |
| S | 8.62 |  | 8 | 2 | 0 | 0 | 1940.77 | 970.89 | 647.59 | n.d. | 970.84 | n.d. | 441.20 (N1-PROC) | 366.02 (H1N1) | 1176.38 (H6N1) |  |  |  |
|  |  |  |  |  |  |  |  |  |  |  |  |  | 644.38 (N2-PROC) | 528.19 (H2N1) | 1338.46 (H7N1) |  |  |  |
|  |  |  |  |  |  |  |  |  |  |  |  |  | 806.38 (H1N2-PROC) | 690.13 (H3N1) | 1500.46 (H8N1) |  |  |  |
|  |  |  |  |  |  |  |  |  |  |  |  |  | 1130.50 (H3N2-PROC) | 852.26 (H4N1) |  |  |  |  |
|  |  |  |  |  |  |  |  |  |  |  |  |  | 1292.54 (H4N2-PROC) | 1014.38 (H5N1) |  |  |  |  |
| T | 8.92 |  | 5 | 4 | 3 | 0 | 2298.95 | 1149.98 | 766.99 | n.d. | 1149.90 | 766.97 | 441.13 (N1-PROC) | 1130.38 (H3N2-PROC) | 674.21 (H2N1F1) | 1201.38 (H4N2F1) |  |  |
|  |  |  |  |  |  |  |  |  |  |  |  |  | 587.42 (N1F1-PROC) | 1276.58 (H3N2F1-PROC) | 690.75 (H3N1) |  |  |  |
|  |  |  |  |  |  |  |  |  |  |  |  |  | 789.88 (N2F1-PROC) | 366.06 (H1N1) | 731.67 (H2N2) |  |  |  |
|  |  |  |  |  |  |  |  |  |  |  |  |  | 968.39 (H2N2-PROC) | 512.18 (H1N1F1) | 893.95 (H3N2) |  |  |  |
|  |  |  |  |  |  |  |  |  |  |  |  |  | 1114.13 (H2N2F1-PROC) | 528.13 (H2N1) | 1055.25 (H4N2) |  |  |  |
| U | 9.02 |  | 5 | 5 | 3 | 0 | 2502.03 | 1251.52 | 834.68 | n.d. | n.d. | 834.66 | 587.38 (N1F1-PROC) | 366.08 (H1N1) | 1550.38 (H4N3F2) |  |  |  |
|  |  |  |  |  |  |  |  |  |  |  |  |  | 806.14 (H1N2-PROC) | 512.22 (H1N1F1) |  |  |  |  |
|  |  |  |  |  |  |  |  |  |  |  |  |  | 1114.50 (H2N2F1-PROC) | 877.38 (H2N2F1) |  |  |  |  |
|  |  |  |  |  |  |  |  |  |  |  |  |  | 1479.55 (H3N3F1-PROC) | 1404.63 (H4N3F1) |  |  |  |  |
|  |  |  |  |  |  |  |  |  |  |  |  |  | 1625.75 (H3N3F2-PROC) | 1420.75 (H5N3) |  |  |  |  |
| V | 9.32 |  | 9 | 2 | 0 | 0 | 2102.83 | 1051.91 | 701.61 | n.d. | 1051.87 | n.d. | 441.19 (N1-PROC) | 1292.50 (H4N2-PROC) | 690.30 (H3N1) | 1500.55 (H8N1) |  |  |
|  |  |  |  |  |  |  |  |  |  |  |  |  | 644.25 (N2-PROC) | 1454.75 (H5N2-PROC) | 852.21 (H4N1) | 1662.60 (H9N1) |  |  |
|  |  |  |  |  |  |  |  |  |  |  |  |  | 806.38 (H1N2-PROC( | 1616.63 (H6N2-PROC) | 1014.35 (H5N1) |  |  |  |
|  |  |  |  |  |  |  |  |  |  |  |  |  | 968.50 (H2N2-PROC) | 366.13 (H1N1) | 1176.47 (H6N1) |  |  |  |
|  |  |  |  |  |  |  |  |  |  |  |  |  | 1130.63 (H3N2-PROC) | 528.21 (H2N1) | 1338.38 (H7N1) |  |  |  |

**Table F.** Structural characterization of procainamide labelled HT29 human colorectal cancer cell line *N-*glycans from experiment 2. Structures for *N-*glycans are depicted following the Consortium for Functional Glycomics (CFG) notation: *N*-acetylglucosamine (N; blue square), fucose (F; red triangle), galactose (H; yellow circle), mannose (H; green circle), *N*-acetylneuraminic acid (S; purple diamond). Glycan compositions are given in the terms of hexose (H), *N*-acetylhexosamine (N), deoxyhexose (F), *N*-acetylneuraminic acid (S).

**HCT15**

15

20

25

30

35

40

45

50

55

**Time [min]**

**A**

**B**

**C**

**D**

**E**

**F**

**G**

**H**

**I**

**J**

**K**

**L**

**M**

**N**

**O**

**P**

**Q**

**R**

**S**

**T**

**U**

**V**

|  | |  |  | | | |  | | | | | | | | | | | |
| --- | --- | --- | --- | --- | --- | --- | --- | --- | --- | --- | --- | --- | --- | --- | --- | --- | --- | --- |
|  | |  |  | | | |  | | | | | | | | | | | |
| Average | | Possible structure | Composition | | | | HCT15 LC-ESI-MS (in-solution PNGaseF release) | | | | | | | | | | | |
| Peak ID | GU (Procainamide) |  |  |  |  |  | [M/Z]^+^ calculated | [M/Z]^2+^ calculated | [M/Z]^3+^ calculated | [M/Z]^+^ registered | [M/Z]^2+^ registered | [M/Z]^3+^ registered | [M/Z] characteristic fragment ions (composition) | | | | | |
|  |  |  | Hex (H) | HexNAc (N) | Fuc (F) | Neu5Ac (S) |  |  |  |  |  |  |  |  |  |  |  |  |
| A | 3.72 |  | 2 | 2 | 1 | 0 | 1114.51 | 557.76 | 372.18 | 1114.50 | 557.75 | n.d. | 441.30 (N1-PROC) |  |  |  |  |  |
|  |  |  |  |  |  |  |  |  |  |  |  |  | 587.31 (N1F1-PROC) |  |  |  |  |  |
|  |  |  |  |  |  |  |  |  |  |  |  |  | 644.35 (N2-PROC) |  |  |  |  |  |
|  |  |  |  |  |  |  |  |  |  |  |  |  | 806.41 (H1N2-PROC) |  |  |  |  |  |
|  |  |  |  |  |  |  |  |  |  |  |  |  | 968.42 (H1N2-PROC) |  |  |  |  |  |
| A | 3.72 |  | 3 | 1 | 0 | 0 | 927.43 | 464.22 | 309.81 | 927.46 | n.d. | n.d. | 441.25 (N1-PROC) |  |  |  |  |  |
|  |  |  |  |  |  |  |  |  |  |  |  |  | 603.32 (H1N1-PROC) |  |  |  |  |  |
|  |  |  |  |  |  |  |  |  |  |  |  |  | 765.35 (H2N1-PROC) |  |  |  |  |  |
|  |  |  |  |  |  |  |  |  |  |  |  |  |  |  |  |  |  |  |
|  |  |  |  |  |  |  |  |  |  |  |  |  |  |  |  |  |  |  |
| B | 3.83 |  | 3 | 1 | 0 | 0 | 927.43 | 464.22 | 309.81 | 927.40 | n.d. | n.d. | 441.18 (N1-PROC) |  |  |  |  |  |
|  |  |  |  |  |  |  |  |  |  |  |  |  | 603.34 (H1N1-PROC) |  |  |  |  |  |
|  |  |  |  |  |  |  |  |  |  |  |  |  | 765.45 (H2N1-PROC) |  |  |  |  |  |
|  |  |  |  |  |  |  |  |  |  |  |  |  |  |  |  |  |  |  |
|  |  |  |  |  |  |  |  |  |  |  |  |  |  |  |  |  |  |  |
| C | 4.24 |  | 3 | 2 | 0 | 0 | 1130.51 | 565.76 | 377.51 | 1130.48 | 565.31 | n.d. | 441.20 (N1-PROC) |  |  |  |  |  |
|  |  |  |  |  |  |  |  |  |  |  |  |  | 644.46 (N2-PROC) |  |  |  |  |  |
|  |  |  |  |  |  |  |  |  |  |  |  |  | 806.13 (H1N2-PROC) |  |  |  |  |  |
|  |  |  |  |  |  |  |  |  |  |  |  |  | 968.53 (H2N2-PROC) |  |  |  |  |  |
|  |  |  |  |  |  |  |  |  |  |  |  |  | 366.63 (H1N1) |  |  |  |  |  |
| D | 4.75 |  | 4 | 1 | 0 | 0 | 1089.48 | 545.24 | 363.83 | 1089.47 | 545.25 | n.d. | 441.14 (N1-PROC) |  |  |  |  |  |
|  |  |  |  |  |  |  |  |  |  |  |  |  | 603.36 (H1N1-PROC) |  |  |  |  |  |
|  |  |  |  |  |  |  |  |  |  |  |  |  | 765.48 (H2N1-PROC) |  |  |  |  |  |
|  |  |  |  |  |  |  |  |  |  |  |  |  | 927.37 (H3N1-PROC) |  |  |  |  |  |
|  |  |  |  |  |  |  |  |  |  |  |  |  |  |  |  |  |  |  |
| D | 4.75 |  | 3 | 2 | 1 | 0 | 1276.57 | 638.79 | 426.19 | 1276.58 | 638.79 | n.d. | 441.21 (N1-PROC) | 1130.56 (H3N2-PROC) |  |  |  |  |
|  |  |  |  |  |  |  |  |  |  |  |  |  | 587.31 (N1F1-PROC) | 528.13 (H2N1) |  |  |  |  |
|  |  |  |  |  |  |  |  |  |  |  |  |  | 644.39 (N2-PROC) |  |  |  |  |  |
|  |  |  |  |  |  |  |  |  |  |  |  |  | 806.40 (H1N2-PROC) |  |  |  |  |  |
|  |  |  |  |  |  |  |  |  |  |  |  |  | 968.39 (H2N2-PROC) |  |  |  |  |  |
| E | 5.16 | - | 4 | 2 | 0 | 0 | 1292.56 | 646.78 | 431.53 | 1292.52 | 646.78 | n.d. |  |  |  |  |  |  |
|  |  |  |  |  |  |  |  |  |  |  |  |  |  |  |  |  |  |  |
|  |  |  |  |  |  |  |  |  |  |  |  |  | No MS/MS data detected |  |  |  |  |  |
|  |  |  |  |  |  |  |  |  |  |  |  |  |  |  |  |  |  |  |
|  |  |  |  |  |  |  |  |  |  |  |  |  |  |  |  |  |  |  |
| F | 5.27 |  | 3 | 3 | 1 | 0 | 1479.65 | 740.33 | 493.89 | n.d. | 740.32 | n.d. | 587.27 (N1F1-PROC) |  |  |  |  |  |
|  |  |  |  |  |  |  |  |  |  |  |  |  | 1114.62 (H2N2F1-PROC) |  |  |  |  |  |
|  |  |  |  |  |  |  |  |  |  |  |  |  | 1130.58 (H3N2-PROC) |  |  |  |  |  |
|  |  |  |  |  |  |  |  |  |  |  |  |  | 1276.56 (H3N2F1-PROC) |  |  |  |  |  |
|  |  |  |  |  |  |  |  |  |  |  |  |  | 893.33 (H3N2) |  |  |  |  |  |
| G | 5.61 |  | 5 | 1 | 0 | 0 | 1251.53 | 626.27 | 417.85 | 1251.53 | 626.27 | n.d. | 441.22 (N1-PROC) |  |  |  |  |  |
|  |  |  |  |  |  |  |  |  |  |  |  |  | 603.29 (H1N1-PROC) |  |  |  |  |  |
|  |  |  |  |  |  |  |  |  |  |  |  |  | 765.37 (H2N1-PROC) |  |  |  |  |  |
|  |  |  |  |  |  |  |  |  |  |  |  |  | 927.37 (H3N1-PROC) |  |  |  |  |  |
|  |  |  |  |  |  |  |  |  |  |  |  |  | 1089.48 (H4N1-PROC) |  |  |  |  |  |
| H | 6.01 |  | 5 | 2 | 0 | 0 | 1454.61 | 727.81 | 485.54 | 1454.58 | 727.80 | n.d. | 441.18 (N1-PROC) | 690.26 (H3N1) |  |  |  |  |
|  |  |  |  |  |  |  |  |  |  |  |  |  | 644.75 (N2-PROC) | 852.21 (H4N1) |  |  |  |  |
|  |  |  |  |  |  |  |  |  |  |  |  |  | 969.38 (H2N2-PROC) |  |  |  |  |  |
|  |  |  |  |  |  |  |  |  |  |  |  |  | 366.13 (H1N1) |  |  |  |  |  |
|  |  |  |  |  |  |  |  |  |  |  |  |  | 528.13 (H2N1) |  |  |  |  |  |
| I | 6.42 |  | 6 | 1 | 0 | 0 | 1413.59 | 707.30 | 471.87 | 1413.50 | 707.28 | n.d. | 441.13 (N1-PROC) |  |  |  |  |  |
|  |  |  |  |  |  |  |  |  |  |  |  |  | 603.44 (H1N1-PROC) |  |  |  |  |  |
|  |  |  |  |  |  |  |  |  |  |  |  |  | 765.14 (H2N1-PROC) |  |  |  |  |  |
|  |  |  |  |  |  |  |  |  |  |  |  |  | 927.32 (H3N1-PROC) |  |  |  |  |  |
|  |  |  |  |  |  |  |  |  |  |  |  |  |  |  |  |  |  |  |
| J | 6.92 |  | 6 | 2 | 0 | 0 | 1616.67 | 808.84 | 539.56 | 1616.63 | 808.82 | n.d. | 441.22 (N1-PROC) | 366.05 (H1N1) | 1176.35 (H6N1) |  |  |  |
|  |  |  |  |  |  |  |  |  |  |  |  |  | 644.25 (N2-PROC) | 528.13 (H2N1) |  |  |  |  |
|  |  |  |  |  |  |  |  |  |  |  |  |  | 806.38 (H1N2-PROC) | 690.29 (H3N1) |  |  |  |  |
|  |  |  |  |  |  |  |  |  |  |  |  |  | 968.38 (H2N2-PROC) | 852.25 (H4N1) |  |  |  |  |
|  |  |  |  |  |  |  |  |  |  |  |  |  | 1130.63 (H3N2-PROC) | 1014.34 (H5N1) |  |  |  |  |
| K | 7.12 |  | 7 | 1 | 0 | 0 | 1575.64 | 788.32 | 525.88 | n.d. | 788.29 | n.d. | 441.21 (N1-PROC) | 487.07 (H3) |  |  |  |  |
|  |  |  |  |  |  |  |  |  |  |  |  |  | 603.34 (H1N1-PROC) | 811.15 (H5) |  |  |  |  |
|  |  |  |  |  |  |  |  |  |  |  |  |  | 765.29 (H2N1-PROC) |  |  |  |  |  |
|  |  |  |  |  |  |  |  |  |  |  |  |  | 927.42 (H3N1-PROC) |  |  |  |  |  |
|  |  |  |  |  |  |  |  |  |  |  |  |  | 1089.51 (H4N1-PROC) |  |  |  |  |  |
| L | 7.43 |  | 5 | 4 | 1 | 0 | 2006.83 | 1003.92 | 669.62 | n.d. | 1003.92 | n.d. | 587.50 (N1F1-PROC) | 1641.73 (H4N3F1-PROC) |  |  |  |  |
|  |  |  |  |  |  |  |  |  |  |  |  |  | 968.75 (H2N2-PROC) | 366.12 (H1N1) |  |  |  |  |
|  |  |  |  |  |  |  |  |  |  |  |  |  | 1277.38 (H3N2F1-PROC) | 512.00 (H1N1F1) |  |  |  |  |
|  |  |  |  |  |  |  |  |  |  |  |  |  | 1479.50 (H3N3F1-PROC) |  |  |  |  |  |
|  |  |  |  |  |  |  |  |  |  |  |  |  | 1495.63 (H4N3-PROC) |  |  |  |  |  |
| M | 7.53 |  | 7 | 1 | 0 | 0 | 1575.64 | 788.32 | 525.88 | n.d. | 788.28 | n.d. | 441.21 (N1-PROC) |  |  |  |  |  |
|  |  |  |  |  |  |  |  |  |  |  |  |  | 603.13 (H1N1-PROC) |  |  |  |  |  |
|  |  |  |  |  |  |  |  |  |  |  |  |  | 765.25 (H2N1-PROC) |  |  |  |  |  |
|  |  |  |  |  |  |  |  |  |  |  |  |  | 927.43 (H3N1-PROC) |  |  |  |  |  |
|  |  |  |  |  |  |  |  |  |  |  |  |  | 1089.63 (H4N1-PROC) |  |  |  |  |  |
| N | 7.82 |  | 7 | 2 | 0 | 0 | 1778.72 | 889.86 | 593.58 | n.d. | 889.83 | n.d. | 441.20 (N1-PROC) | 366.00 (H1N1) | 1176.38 (H6N1) |  |  |  |
|  |  |  |  |  |  |  |  |  |  |  |  |  | 644.36 (N2-PROC) | 528.00 (H2N1) | 1338.38 (H7N1) |  |  |  |
|  |  |  |  |  |  |  |  |  |  |  |  |  | 806.38 (H1N2-PROC) | 690.38 (H3N1) |  |  |  |  |
|  |  |  |  |  |  |  |  |  |  |  |  |  | 968.38 (H2N2-PROC) | 852.25 (H4N1) |  |  |  |  |
|  |  |  |  |  |  |  |  |  |  |  |  |  | 1292.51 (H4N2-PROC) | 1014.13 (H5N1) |  |  |  |  |
| O | 8.13 |  | 8 | 1 | 0 | 0 | 1737.69 | 869.35 | 579.90 | n.d. | 869.28 | n.d. | 442.26 (N1-PROC) | 1251.76 (H5N1-PROC) |  |  |  |  |
|  |  |  |  |  |  |  |  |  |  |  |  |  | 604.13 (H1N1-PROC) | 325.75 (H2) |  |  |  |  |
|  |  |  |  |  |  |  |  |  |  |  |  |  | 766.25 (H2N1-PROC) | 487.13 (H3) |  |  |  |  |
|  |  |  |  |  |  |  |  |  |  |  |  |  | 927.50 (H3N1-PROC) | 811.25 (H5) |  |  |  |  |
|  |  |  |  |  |  |  |  |  |  |  |  |  | 1089.50 (H4N1-PROC) |  |  |  |  |  |
| P | 8.43 |  | 8 | 1 | 0 | 0 | 1737.69 | 869.35 | 579.90 | n.d. | 869.30 | n.d. | 442.01 (N1-PROC) | 1251.54 (H5N1-PROC) | 973.25 (H6) |  |  |  |
|  |  |  |  |  |  |  |  |  |  |  |  |  | 604.40 (H1N1-PROC) | 324.14 (H2) | 1136.75 (H7 |  |  |  |
|  |  |  |  |  |  |  |  |  |  |  |  |  | 766.25 (H2N1-PROC) | 486.76 (H3) |  |  |  |  |
|  |  |  |  |  |  |  |  |  |  |  |  |  | 927.88 (H3N1-PROC) | 649.29 (H4) |  |  |  |  |
|  |  |  |  |  |  |  |  |  |  |  |  |  | 1089.38 (H4N1-PROC) | 812.38 (H5) |  |  |  |  |
| Q | 8.54 |  | 5 | 4 | 1 | 1 | 2297.93 | 1149.47 | 766.65 | n.d. | 1149.91 | 766.67 | 440.88 (N1-PROC) | 366.09 (H1N1) | 819.38 (H2N1S1) |  |  |  |
|  |  |  |  |  |  |  |  |  |  |  |  |  | 790.88 (N2F1-PROC) | 512.11 (H1N1F1) | 893.39 (H3N2) |  |  |  |
|  |  |  |  |  |  |  |  |  |  |  |  |  | 968.38 (H2N2-PROC) | 658.13 (H1N1S1) |  |  |  |  |
|  |  |  |  |  |  |  |  |  |  |  |  |  | 1115.38 (H2N2F1-PROC) | 674.25 (H2N1F1) |  |  |  |  |
|  |  |  |  |  |  |  |  |  |  |  |  |  | 1276.54 (H3N2F1-PROC) | 731.51 (H2N2) |  |  |  |  |
| R | 8.74 |  | 8 | 2 | 0 | 0 | 1940.77 | 970.89 | 647.60 | n.d. | 970.84 | n.d. | 441.24 (N1-PROC) | 1454.49 (H5N2-PROC) | 1014.33 (H5N1) |  |  |  |
|  |  |  |  |  |  |  |  |  |  |  |  |  | 644.39 (N2-PROC) | 366.00 (H1N1) | 1176.33 (H6N1) |  |  |  |
|  |  |  |  |  |  |  |  |  |  |  |  |  | 806.38 (H1N2-PROC) | 528.15 (H2N1) | 1338.42 (H7N1) |  |  |  |
|  |  |  |  |  |  |  |  |  |  |  |  |  | 1130.48 (H3N2-PROC) | 690.19 (H3N1) | 1500.51 (H8N1) |  |  |  |
|  |  |  |  |  |  |  |  |  |  |  |  |  | 1292.54 (H4N2-PROC) | 852.29 (H4N1) |  |  |  |  |
| S | 8.84 |  | 6 | 5 | 0 | 1 | 2517.00 | 1259.00 | 839.67 | n.d. | n.d. | 839.64 | 1333.50 (H3N3-PROC) | 658.25 (H1N1S1) |  |  |  |  |
|  |  |  |  |  |  |  |  |  |  |  |  |  | 1495.50 (H4N3-PROC) | 690.50 (H3N1) |  |  |  |  |
|  |  |  |  |  |  |  |  |  |  |  |  |  | 1787.63 (H4N3S1-PROC) | 819.24 (H2N1S1) |  |  |  |  |
|  |  |  |  |  |  |  |  |  |  |  |  |  | 366.02 (H1N1) | 893.25 (H3N2) |  |  |  |  |
|  |  |  |  |  |  |  |  |  |  |  |  |  | 528.25 (H2N1) |  |  |  |  |  |
| T | 8.94 |  | 5 | 4 | 1 | 1 | 2297.93 | 1149.47 | 766.65 | n.d. | n.d. | 766.73 | 967.38 (H2N2-PROC) | 894.36 (H3N2) |  |  |  |  |
|  |  |  |  |  |  |  |  |  |  |  |  |  | 1131.75 (H3N2-PROC) | 998.76 (H4N1F1) |  |  |  |  |
|  |  |  |  |  |  |  |  |  |  |  |  |  | 1495.13 (H4N3-PROC) |  |  |  |  |  |
|  |  |  |  |  |  |  |  |  |  |  |  |  | 366.88 (H1N1) |  |  |  |  |  |
|  |  |  |  |  |  |  |  |  |  |  |  |  | 512.08 (H1N1F1) |  |  |  |  |  |
| U | 9.14 |  | 6 | 5 | 3 | 0 | 2664.08 | 1332.54 | 888.70 | n.d. | n.d. | 888.67 | 441.25 (N1-PROC) | 1509.38 (H5N2F2) |  |  |  |  |
|  |  |  |  |  |  |  |  |  |  |  |  |  | 806.66 (H1N2-PROC) | 1641.78 (H4N2F1) |  |  |  |  |
|  |  |  |  |  |  |  |  |  |  |  |  |  | 366.13 (H1N1) | 1916.63 (H5N4F2) |  |  |  |  |
|  |  |  |  |  |  |  |  |  |  |  |  |  | 512.07 (H1N1F1) |  |  |  |  |  |
|  |  |  |  |  |  |  |  |  |  |  |  |  | 1185.26 (H3N2F2) |  |  |  |  |  |
| V | 9.34 |  | 9 | 2 | 0 | 0 | 2102.83 | 1051.92 | 701.61 | n.d. | 1051.87 | n.d. | 441.19 (N1-PROC) | 1292.59 (H4N2-PROC) | 690.22 (H3N1) | 1500.50 (H8N1) |  |  |
|  |  |  |  |  |  |  |  |  |  |  |  |  | 644.39 (N2-PROC) | 1455.52 (H5N2-PROC) | 852.26 (H4N1) | 1662.54 (H9N1) |  |  |
|  |  |  |  |  |  |  |  |  |  |  |  |  | 806.50 (H1N2-PROC) | 1617.63 (H6N2-PROC) | 1014.30 (H5N1) |  |  |  |
|  |  |  |  |  |  |  |  |  |  |  |  |  | 968.50 (H2N2-PROC) | 365.62 (H1N1) | 1176.38 (H6N1) |  |  |  |
|  |  |  |  |  |  |  |  |  |  |  |  |  | 1130.59 (H3N2-PROC) | 528.11 (H2N1) | 1338.54 (H7N1) |  |  |  |

**Table G.** Structural characterization of procainamide labelled HCT15 human colorectal cancer cell line *N-*glycans from experiment 2. Structures for *N-*glycans are depicted following the Consortium for Functional Glycomics (CFG) notation: *N*-acetylglucosamine (N; blue square), fucose (F; red triangle), galactose (H; yellow circle), mannose (H; green circle), *N*-acetylneuraminic acid (S; purple diamond). Glycan compositions are given in the terms of hexose (H), *N*-acetylhexosamine (N), deoxyhexose (F), *N*-acetylneuraminic acid (S).

**HCT116**

15

20

25

30

35

40

45

50

55

**Time [min]**

**A**

**B**

**C**

**E**

**D**

**F**

**G**

**H**

**I**

**J**

**K**

**L**

**M**

**N**

**O**

|  | |  |  | | | |  | | | | | | | | | | | |
| --- | --- | --- | --- | --- | --- | --- | --- | --- | --- | --- | --- | --- | --- | --- | --- | --- | --- | --- |
|  | |  |  | | | |  | | | | | | | | | | | |
| Average | | Possible structure | Composition | | | | HCT116 LC-ESI-MS (in-solution PNGaseF release) | | | | | | | | | | | |
| Peak ID | GU (Procainamide) |  |  |  |  |  | [M/Z]^+^ calculated | [M/Z]^2+^ calculated | [M/Z]^3+^ calculated | [M/Z]^+^ registered | [M/Z]^2+^ registered | [M/Z]^3+^ registered | [M/Z] characteristic fragment ions (composition) | | | | | |
|  |  |  | Hex (H) | HexNAc (N) | Fuc (F) | Neu5Ac (S) |  |  |  |  |  |  |  |  |  |  |  |  |
| A | 3.71 |  | 3 | 1 | 0 | 0 | 927.43 | 464.22 | 309.81 | 927.39 | n.d. | n.d. | 441.20 (N1-PROC) |  |  |  |  |  |
|  |  |  |  |  |  |  |  |  |  |  |  |  | 603.27 (H1N1-PROC) |  |  |  |  |  |
|  |  |  |  |  |  |  |  |  |  |  |  |  | 765.36 (H2N1-PROC) |  |  |  |  |  |
|  |  |  |  |  |  |  |  |  |  |  |  |  |  |  |  |  |  |  |
|  |  |  |  |  |  |  |  |  |  |  |  |  |  |  |  |  |  |  |
| B | 4.22 |  | 3 | 2 | 0 | 0 | 1130.51 | 565.76 | 377.51 | 1130.52 | 565.76 | n.d. | 441.21 (N1-PROC) | 690.25 (H3N1) |  |  |  |  |
|  |  |  |  |  |  |  |  |  |  |  |  |  | 644.35 (N2-PROC) |  |  |  |  |  |
|  |  |  |  |  |  |  |  |  |  |  |  |  | 806.38 (H1N2-PROC) |  |  |  |  |  |
|  |  |  |  |  |  |  |  |  |  |  |  |  | 968.45 (H2N2-PROC) |  |  |  |  |  |
|  |  |  |  |  |  |  |  |  |  |  |  |  | 528.24 (H2N1) |  |  |  |  |  |
| C | 6.03 |  | 5 | 2 | 0 | 0 | 1454.61 | 727.81 | 485.54 | 1454.59 | 727.80 | n.d. | 441.23 (N1-PROC) |  |  |  |  |  |
|  |  |  |  |  |  |  |  |  |  |  |  |  | 644.25 (N2-PROC) |  |  |  |  |  |
|  |  |  |  |  |  |  |  |  |  |  |  |  | 690.16 (H3N1) |  |  |  |  |  |
|  |  |  |  |  |  |  |  |  |  |  |  |  | 852.27 (H4N1) |  |  |  |  |  |
|  |  |  |  |  |  |  |  |  |  |  |  |  | 1014.33 (H5N1) |  |  |  |  |  |
| D | 6.54 | - | 5 | 3 | 0 | 0 | 1657.69 | 829.35 | 553.24 | n.d. | 829.33 | 553.24 |  |  |  |  |  |  |
|  |  |  |  |  |  |  |  |  |  |  |  |  |  |  |  |  |  |  |
|  |  |  |  |  |  |  |  |  |  |  |  |  | No MS/MS data detected |  |  |  |  |  |
|  |  |  |  |  |  |  |  |  |  |  |  |  |  |  |  |  |  |  |
|  |  |  |  |  |  |  |  |  |  |  |  |  |  |  |  |  |  |  |
| E | 6.65 |  | 4 | 3 | 0 | 1 | 1786.74 | 893.87 | 596.25 | n.d. | 893.84 | 596.23 | 441.17 (N1-PROC) | 852.63 (H4N1) |  |  |  |  |
|  |  |  |  |  |  |  |  |  |  |  |  |  | 968.51 (H2N2-PROC) | 1346.51 (H4N2S1) |  |  |  |  |
|  |  |  |  |  |  |  |  |  |  |  |  |  | 1130.50 (H3N2-PROC) |  |  |  |  |  |
|  |  |  |  |  |  |  |  |  |  |  |  |  | 365.99 (H1N1) |  |  |  |  |  |
|  |  |  |  |  |  |  |  |  |  |  |  |  | 657.20 (H1N1S1) |  |  |  |  |  |
| F | 6.96 |  | 6 | 2 | 0 | 0 | 1616.67 | 808.84 | 539.56 | 1616.65 | 808.81 | n.d. | 441.18 (N1-PROC) | 690.21 (H3N1) |  |  |  |  |
|  |  |  |  |  |  |  |  |  |  |  |  |  | 644.38 (N2-PROC) | 852.21 (H4N1) |  |  |  |  |
|  |  |  |  |  |  |  |  |  |  |  |  |  | 806.35 (H1N2-PROC) | 1014.24 (H5N1) |  |  |  |  |
|  |  |  |  |  |  |  |  |  |  |  |  |  | 366.25 (H1N1) | 1176.39 (H6N1) |  |  |  |  |
|  |  |  |  |  |  |  |  |  |  |  |  |  | 528.09 (H2N1) |  |  |  |  |  |
| G | 7.07 |  | 5 | 4 | 0 | 0 | 1860.77 | 930.89 | 620.93 | n.d. | 930.86 | 620.91 | 441.38 (N1-PROC) | 1055.38 (H4N2) |  |  |  |  |
|  |  |  |  |  |  |  |  |  |  |  |  |  | 968.38 (H2N2-PROC) | 1420.50 (H5N3) |  |  |  |  |
|  |  |  |  |  |  |  |  |  |  |  |  |  | 1495.62 (H4N3-PROC) |  |  |  |  |  |
|  |  |  |  |  |  |  |  |  |  |  |  |  | 366.02 (H1N1) |  |  |  |  |  |
|  |  |  |  |  |  |  |  |  |  |  |  |  | 893.53 (H3N2) |  |  |  |  |  |
| H | 7.38 |  | 3 | 5 | 0 | 1 | 1948.79 | 974.89 | 650.27 | n.d. | 974.87 | n.d. | 441.38 (N1-PROC) | 366.02 (H1N1) |  |  |  |  |
|  |  |  |  |  |  |  |  |  |  |  |  |  | 927.47 (H3N1-PROC) | 657.21 (H1N1S1) |  |  |  |  |
|  |  |  |  |  |  |  |  |  |  |  |  |  | 1130.55 (H3N2-PROC) | 819.20 (H2N1S1) |  |  |  |  |
|  |  |  |  |  |  |  |  |  |  |  |  |  | 1292.56 (H4N2-PROC) | 852.38 (H4N1) |  |  |  |  |
|  |  |  |  |  |  |  |  |  |  |  |  |  | 1583.50 (H4N2S1-PROC) | 893.75 (H3N2) |  |  |  |  |
| I | 7.49 |  | 6 | 3 | 0 | 0 | 1819.75 | 910.38 | 607.25 | n.d. | 910.36 | 607.28 | 441.25 (N1-PROC) | 1379.38 (H6N2) |  |  |  |  |
|  |  |  |  |  |  |  |  |  |  |  |  |  | 1292.58 (H4N2-PROC) |  |  |  |  |  |
|  |  |  |  |  |  |  |  |  |  |  |  |  | 1454.63 (H5N2-PROC) |  |  |  |  |  |
|  |  |  |  |  |  |  |  |  |  |  |  |  | 366.05 (H1N1) |  |  |  |  |  |
|  |  |  |  |  |  |  |  |  |  |  |  |  | 528.25 (H2N1) |  |  |  |  |  |
| I | 7.49 |  | 5 | 4 | 0 | 1 | 2151.87 | 1076.44 | 717.96 | n.d. | 1076.42 | 717.95 | 441.00 (N1-PROC) | 1495.68 (H4N3-PROC) | 657.21 (H1N1S1) |  |  |  |
|  |  |  |  |  |  |  |  |  |  |  |  |  | 644.50 (N2-PROC) | 1624.50 (H3N3S1-PROC) | 1055.76 (H4N2) |  |  |  |
|  |  |  |  |  |  |  |  |  |  |  |  |  | 1130.51 (H3N2-PROC) | 1786.88 (H4N3S1-PROC) |  |  |  |  |
|  |  |  |  |  |  |  |  |  |  |  |  |  | 1171.53 (H2N3-PROC) | 365.99 (H1N1) |  |  |  |  |
|  |  |  |  |  |  |  |  |  |  |  |  |  | 1333.54 (H3N3-PROC) | 528.13 (H2N1) |  |  |  |  |
| J | 7.71 |  | 7 | 2 | 0 | 0 | 1778.72 | 889.86 | 593.58 | n.d. | 889.82 | n.d. | 441.22 (N1-PROC) | 366.11 (H1N1) | 1176.46 (H6N1) |  |  |  |
|  |  |  |  |  |  |  |  |  |  |  |  |  | 644.32 (N2-PROC) | 528.13 (H2N1) | 1338.43 (H7N1) |  |  |  |
|  |  |  |  |  |  |  |  |  |  |  |  |  | 806.38 (H1N2-PROC) | 690.18 (H3N1) |  |  |  |  |
|  |  |  |  |  |  |  |  |  |  |  |  |  | 968.50 (H2N2-PROC) | 852.28 (H4N1) |  |  |  |  |
|  |  |  |  |  |  |  |  |  |  |  |  |  | 1130.88 (H3N2-PROC) | 1014.34 (H5N1) |  |  |  |  |
| K | 8.11 |  | 5 | 4 | 0 | 2 | 2442.96 | 1221.99 | 814.99 | n.d. | 1221.96 | 814.99 | 441.28 (N1-PROC) | 1171.75 (H2N3-PROC) | 366.00 (H1N1) | 1184.41 (H3N2S1) |  |  |
|  |  |  |  |  |  |  |  |  |  |  |  |  | 644.38 (N2-PROC) | 1333.67 (H3N3-PROC) | 528.16 (H2N1) | 1420.50 (H5N3) |  |  |
|  |  |  |  |  |  |  |  |  |  |  |  |  | 806.00 (H1N2-PROC) | 1495.63 (H4N3-PROC) | 657.21 (H1N1S1) |  |  |  |
|  |  |  |  |  |  |  |  |  |  |  |  |  | 968.44 (H2N2-PROC) | 1624.76 (H3N3S1-PROC) | 731.13 (H2N2) |  |  |  |
|  |  |  |  |  |  |  |  |  |  |  |  |  | 1130.50 (H3N2-PROC) | 1786.88 (H4N3S1-PROC) | 893.75 (H3N2) |  |  |  |
| L | 8.61 |  | 8 | 2 | 0 | 0 | 1940.77 | 970.89 | 647.59 | n.d. | 970.84 | n.d. | 441.20 (N1-PROC) | 1454.63 (H5N2-PROC) | 1014.40 (H5N2) |  |  |  |
|  |  |  |  |  |  |  |  |  |  |  |  |  | 644.25 (N2-PROC) | 366.00 (H1N1) | 1176.25 (H6N1) |  |  |  |
|  |  |  |  |  |  |  |  |  |  |  |  |  | 806.36 (H1N2-PROC) | 528.13 (H2N1) | 1338.43 (H7N1) |  |  |  |
|  |  |  |  |  |  |  |  |  |  |  |  |  | 1130.39 (H3N2-PROC) | 690.21 (H3N1) | 1500.38 (H8N1) |  |  |  |
|  |  |  |  |  |  |  |  |  |  |  |  |  | 1292.50 (H4N2-PROC) | 852.22 (H4N1) |  |  |  |  |
| M | 8.81 |  | 6 | 5 | 0 | 1 | 2517.00 | 1259.00 | 839.67 | n.d. | n.d. | 839.64 | 441.12 (N1-PROC) | 1495.75 (H4N3-PROC) | 1184.33 (H3N2S1) |  |  |  |
|  |  |  |  |  |  |  |  |  |  |  |  |  | 644.18 (N2-PROC) | 1624.75 (H3N3S1-PROC) | 1346.51 (H4N2S1) |  |  |  |
|  |  |  |  |  |  |  |  |  |  |  |  |  | 968.50 (H2N2-PROC) | 366.06 (H1N1) | 1420.50 (H5N3) |  |  |  |
|  |  |  |  |  |  |  |  |  |  |  |  |  | 1130.26 (H3N2-PROC) | 657.27 (H1N1S1) |  |  |  |  |
|  |  |  |  |  |  |  |  |  |  |  |  |  | 1333.50 (H3N3-PROC) | 893.75 (H3N2) |  |  |  |  |
| N | 9.01 |  | 6 | 5 | 0 | 2 | 2808.09 | 1404.55 | 936.70 | n.d. | n.d. | 936.65 | 441.26 (N1-PROC) | 2151.75 (H5N4S1-PROC) | 1711.61 (H5N3S1) |  |  |  |
|  |  |  |  |  |  |  |  |  |  |  |  |  | 968.39 (H2N2-PROC) | 366.02 (H1N1) | 1799.38 (H5N2S2) |  |  |  |
|  |  |  |  |  |  |  |  |  |  |  |  |  | 1333.63 (H3N3-PROC) | 657.18 (H1N1S1) |  |  |  |  |
|  |  |  |  |  |  |  |  |  |  |  |  |  | 1495.63 (H4N3-PROC) | 731.75 (H2N2) |  |  |  |  |
|  |  |  |  |  |  |  |  |  |  |  |  |  | 1624.75 (H3N3S1-PROC) | 893.50 (H3N2) |  |  |  |  |
| O | 9.32 |  | 9 | 2 | 0 | 0 | 2102.83 | 1051.92 | 701.61 | n.d. | 1051.86 | n.d. | 441.21 (N1-PROC) | 1292.54 (H4N2-PROC) | 852.23 (H4N1) | 1662.50 (H9N1) |  |  |
|  |  |  |  |  |  |  |  |  |  |  |  |  | 644.35 (N2-PROC) | 1454.75 (H5N2-PROC) | 1014.50 (H5N1) |  |  |  |
|  |  |  |  |  |  |  |  |  |  |  |  |  | 806.38 (H1N2-PROC) | 366.00 (H1N1) | 1176.46 (H6N1) |  |  |  |
|  |  |  |  |  |  |  |  |  |  |  |  |  | 968.38 (H2N2-PROC) | 528.21 (H2N1) | 1338.64 (H7N1) |  |  |  |
|  |  |  |  |  |  |  |  |  |  |  |  |  | 1130.51 (H3N2-PROC) | 690.26 (H3N1) | 1500.51 (H8N1) |  |  |  |

**Table H.** Structural characterization of procainamide labelled HCT116 human colorectal cancer cell line *N-*glycans from experiment 2. Structures for *N-*glycans are depicted following the Consortium for Functional Glycomics (CFG) notation: *N*-acetylglucosamine (N; blue square), fucose (F; red triangle), galactose (H; yellow circle), mannose (H; green circle), *N*-acetylneuraminic acid (S; purple diamond). Glycan compositions are given in the terms of hexose (H), *N*-acetylhexosamine (N), deoxyhexose (F), *N*-acetylneuraminic acid (S).

**KM12**

15

20

25

30

35

40

45

50

55

**Time [min]**

**A**

**B**

**C**

**D**

**E**

**F**

**G**

**H**

**I**

**J**

**K**

**L**

**M**

**N**

**O**

|  | |  |  | | | |  | | | | | | | | | | | |
| --- | --- | --- | --- | --- | --- | --- | --- | --- | --- | --- | --- | --- | --- | --- | --- | --- | --- | --- |
|  | |  |  | | | |  | | | | | | | | | | | |
| Average | | Possible structure | Composition | | | | KM12 LC-ESI-MS (in-solution PNGaseF release) | | | | | | | | | | | |
| Peak ID | GU (Procainamide) |  |  |  |  |  | [M/Z]^+^ calculated | [M/Z]^2+^ calculated | [M/Z]^3+^ calculated | [M/Z]^+^ registered | [M/Z]^2+^ registered | [M/Z]^3+^ registered | [M/Z] characteristic fragment ions (composition) | | | | | |
|  |  |  | Hex (H) | HexNAc (N) | Fuc (F) | Neu5Ac (S) |  |  |  |  |  |  |  |  |  |  |  |  |
| A | 3.71 |  | 2 | 2 | 1 | 0 | 1114.51 | 557.76 | 372.18 | 1114.53 | 557.77 | n.d. | 441.28 (N1-PROC) | 952.38 (H1N2F1-PROC) |  |  |  |  |
|  |  |  |  |  |  |  |  |  |  |  |  |  | 587.33 (N1F1-PROC) | 968.42 (H2N2-PROC) |  |  |  |  |
|  |  |  |  |  |  |  |  |  |  |  |  |  | 644.37 (N2-PROC) | 366.13 (H1N1) |  |  |  |  |
|  |  |  |  |  |  |  |  |  |  |  |  |  | 790.38 (N2F1-PROC) | 529.00 (H2N1) |  |  |  |  |
|  |  |  |  |  |  |  |  |  |  |  |  |  | 806.43 (H1N2-PROC) |  |  |  |  |  |
| B | 4.22 | - | 3 | 2 | 0 | 0 | 1130.51 | 565.76 | 377.51 | 1130.49 | 565.38 | n.d. |  |  |  |  |  |  |
|  |  |  |  |  |  |  |  |  |  |  |  |  |  |  |  |  |  |  |
|  |  |  |  |  |  |  |  |  |  |  |  |  | No MS/MS data detected |  |  |  |  |  |
|  |  |  |  |  |  |  |  |  |  |  |  |  |  |  |  |  |  |  |
|  |  |  |  |  |  |  |  |  |  |  |  |  |  |  |  |  |  |  |
| C | 4.63 |  | 3 | 2 | 1 | 0 | 1276.57 | 638.79 | 426.19 | 1276.52 | 638.79 | n.d. | 441.38 (N1-PROC) |  |  |  |  |  |
|  |  |  |  |  |  |  |  |  |  |  |  |  | 587.32 (N1F1-PROC) |  |  |  |  |  |
|  |  |  |  |  |  |  |  |  |  |  |  |  | 644.27 (N2-PROC) |  |  |  |  |  |
|  |  |  |  |  |  |  |  |  |  |  |  |  |  |  |  |  |  |  |
|  |  |  |  |  |  |  |  |  |  |  |  |  |  |  |  |  |  |  |
| D | 5.04 |  | 3 | 4 | 1 | 0 | 1682.72 | 841.87 | 561.58 | n.d. | 841.74 | n.d. | 441.25 (N1-PROC) |  |  |  |  |  |
|  |  |  |  |  |  |  |  |  |  |  |  |  | 587.63 (N1F1-PROC) |  |  |  |  |  |
|  |  |  |  |  |  |  |  |  |  |  |  |  | 952.13 (H1N2F1-PROC) |  |  |  |  |  |
|  |  |  |  |  |  |  |  |  |  |  |  |  |  |  |  |  |  |  |
|  |  |  |  |  |  |  |  |  |  |  |  |  |  |  |  |  |  |  |
| E | 5.65 | - | 5 | 1 | 0 | 0 | 1251.53 | 626.27 | 417.85 | 1251.45 | n.d. | n.d. |  |  |  |  |  |  |
|  |  |  |  |  |  |  |  |  |  |  |  |  |  |  |  |  |  |  |
|  |  |  |  |  |  |  |  |  |  |  |  |  | No MS/MS data detected |  |  |  |  |  |
|  |  |  |  |  |  |  |  |  |  |  |  |  |  |  |  |  |  |  |
|  |  |  |  |  |  |  |  |  |  |  |  |  |  |  |  |  |  |  |
| F | 6.06 |  | 5 | 2 | 0 | 0 | 1454.61 | 727.81 | 485.54 | 1454.57 | 727.78 | n.d. | 441.24 (N1-PROC) | 1014.29 (H5N1) |  |  |  |  |
|  |  |  |  |  |  |  |  |  |  |  |  |  | 644.38 (N2-PROC) |  |  |  |  |  |
|  |  |  |  |  |  |  |  |  |  |  |  |  | 527.98 (H2N1) |  |  |  |  |  |
|  |  |  |  |  |  |  |  |  |  |  |  |  | 690.23 (H3N1) |  |  |  |  |  |
|  |  |  |  |  |  |  |  |  |  |  |  |  | 852.21 (H4N1) |  |  |  |  |  |
| G | 6.97 |  | 6 | 2 | 0 | 0 | 1616.67 | 808.84 | 539.56 | 1616.65 | 808.81 | n.d. | 441.22 (N1-PROC) | 528.17 (H2N1) |  |  |  |  |
|  |  |  |  |  |  |  |  |  |  |  |  |  | 644.25 (N2-PROC) | 690.20 (H3N1) |  |  |  |  |
|  |  |  |  |  |  |  |  |  |  |  |  |  | 806.38 (H1N2-PROC) | 852.25 (H4N1) |  |  |  |  |
|  |  |  |  |  |  |  |  |  |  |  |  |  | 968.41 (H2N2-PROC) | 1014.32 (H5N1) |  |  |  |  |
|  |  |  |  |  |  |  |  |  |  |  |  |  | 366.04 (H1N1) | 1176.37 (H6N1) |  |  |  |  |
| H | 7.48 |  | 7 | 1 | 0 | 0 | 1575.64 | 788.32 | 525.88 | n.d. | 788.21 | n.d. | 441.37 (N1-PROC) | 811.75 (H5) |  |  |  |  |
|  |  |  |  |  |  |  |  |  |  |  |  |  | 603.33 (H1N1-PROC) | 1135.00 (H7) |  |  |  |  |
|  |  |  |  |  |  |  |  |  |  |  |  |  | 765.58 (H2N1-PROC) |  |  |  |  |  |
|  |  |  |  |  |  |  |  |  |  |  |  |  | 927.51 (H3N1-PROC) |  |  |  |  |  |
|  |  |  |  |  |  |  |  |  |  |  |  |  | 1090.39 (H4N1-PROC) |  |  |  |  |  |
| I | 7.89 |  | 7 | 2 | 0 | 0 | 1778.72 | 889.86 | 593.58 | n.d. | 889.81 | n.d. | 441.19 (N1-PROC) | 366.13 (H1N1) | 1176.37 (H6N1) |  |  |  |
|  |  |  |  |  |  |  |  |  |  |  |  |  | 644.25 (N2-PROC) | 528.18 (H2N1) | 1338.46 (H7N1) |  |  |  |
|  |  |  |  |  |  |  |  |  |  |  |  |  | 806.38 (H1N2-PROC) | 690.24 (H3N1) |  |  |  |  |
|  |  |  |  |  |  |  |  |  |  |  |  |  | 968.50 (H2N2-PROC) | 852.50 (H4N1) |  |  |  |  |
|  |  |  |  |  |  |  |  |  |  |  |  |  | 1130.38 (H3N2-PROC) | 1014.25 (H5N1) |  |  |  |  |
| J | 8.11 |  | 5 | 4 | 1 | 1 | 2297.93 | 1149.47 | 766.65 | n.d. | 1149.88 | 766.64 | 441.11 (N1-PROC) | 1130.60 (H3N2-PROC) | 366.08 (H1N1) |  |  |  |
|  |  |  |  |  |  |  |  |  |  |  |  |  | 587.35 (N1F1-PROC) | 1276.54 (H3N2F1-PROC) | 528.13 (H2N1) |  |  |  |
|  |  |  |  |  |  |  |  |  |  |  |  |  | 644.38 (N2-PROC) | 1479.73 (H3N3F1-PROC) | 657.24 (H1N1S1) |  |  |  |
|  |  |  |  |  |  |  |  |  |  |  |  |  | 968.13 (H2N2) | 1495.75 (H4N3-PROC) | 852.25 (H4N1) |  |  |  |
|  |  |  |  |  |  |  |  |  |  |  |  |  | 1114.54 (H2N2F1-PROC) | 1641.53 (H4N3F1-PROC) | 1420.38 (H5N3) |  |  |  |
| K | 8.61 |  | 8 | 2 | 0 | 0 | 1940.77 | 970.89 | 647.59 | n.d. | 970.82 | n.d. | 441.21 (N1-PROC) | 365.88 (H1N1) | 1176.46 (H6N1) |  |  |  |
|  |  |  |  |  |  |  |  |  |  |  |  |  | 644.35 (N2-PROC) | 528.18 (H2N1) | 1338.43 (H7N1) |  |  |  |
|  |  |  |  |  |  |  |  |  |  |  |  |  | 806.38 (H1N2-PROC) | 690.25 (H3N1) | 1500.47 (H8N1) |  |  |  |
|  |  |  |  |  |  |  |  |  |  |  |  |  | 1130.51 (H3N2-PROC) | 852.28 (H4N1) |  |  |  |  |
|  |  |  |  |  |  |  |  |  |  |  |  |  | 1292.50 (H4N2-PROC) | 1014.29 (H5N1) |  |  |  |  |
| L | 8.81 |  | 6 | 5 | 1 | 0 | 2371.96 | 1186.48 | 791.33 | n.d. | n.d. | 791.33 | 586.50 (N1F1-PROC) | 1420.76 (H5N3) |  |  |  |  |
|  |  |  |  |  |  |  |  |  |  |  |  |  | 1276.33 (H3N2F1-PROC) | 1462.25 (H4N4) |  |  |  |  |
|  |  |  |  |  |  |  |  |  |  |  |  |  | 1642.63 (H4N3F1-PROC) |  |  |  |  |  |
|  |  |  |  |  |  |  |  |  |  |  |  |  | 365.95 (H1N1) |  |  |  |  |  |
|  |  |  |  |  |  |  |  |  |  |  |  |  | 731.50 (H2N2) |  |  |  |  |  |
| L | 8.81 |  | 5 | 4 | 2 | 1 | 2443.98 | 1222.49 | 815.33 | n.d. | 1222.96 | 815.63 | 587.45 (N1F1-PROC) | 1276.63 (H3N2F1-PROC) | 658.13 (H1N1S1) |  |  |  |
|  |  |  |  |  |  |  |  |  |  |  |  |  | 644.75 (N2-PROC) | 1479.75 (H3N3F1-PROC) | 690.70 (H3N1) |  |  |  |
|  |  |  |  |  |  |  |  |  |  |  |  |  | 806.38 (H1N2-PROC) | 1786.13 (H4N3S1-PROC) | 730.75 (H2N2) |  |  |  |
|  |  |  |  |  |  |  |  |  |  |  |  |  | 967.88 (H2N2-PROC) | 365.90 (H1N1) | 1040.08 (H3N2F1) |  |  |  |
|  |  |  |  |  |  |  |  |  |  |  |  |  | 1114.63 (H2N2F1-PROC) | 512.13 (H1N1F1) | 1055.38 (H4N2) |  |  |  |
| M | 9.01 |  | 9 | 1 | 0 | 0 | 1899.75 | 950.38 | 633.92 | n.d. | 950.30 | n.d. | 441.25 (N1-PROC) | 1251.46 (H5N1-PROC) |  |  |  |  |
|  |  |  |  |  |  |  |  |  |  |  |  |  | 603.29 (H1N1-PROC) | 1413.50 (H6N1-PROC) |  |  |  |  |
|  |  |  |  |  |  |  |  |  |  |  |  |  | 765.50 (H2N1-PROC) | 1576.26 (H7N1-PROC) |  |  |  |  |
|  |  |  |  |  |  |  |  |  |  |  |  |  | 927.38 (H3N1-PROC) | 852.38 (H4N1) |  |  |  |  |
|  |  |  |  |  |  |  |  |  |  |  |  |  | 1089.56 (H4N1-PROC) |  |  |  |  |  |
| N | 9.31 |  | 9 | 2 | 0 | 0 | 2102.83 | 1051.92 | 701.61 | n.d. | 1051.85 | n.d. | 441.18 (N1-PROC) | 1292.67 (H4N2-PROC) | 690.24 (H3N1) | 1500.53 (H8N1) |  |  |
|  |  |  |  |  |  |  |  |  |  |  |  |  | 644.38 (N2-PROC) | 1454.39 (H5N2-PROC) | 852.19 (H4N1) | 1662.50 (H9N1) |  |  |
|  |  |  |  |  |  |  |  |  |  |  |  |  | 806.50 (H1N2-PROC) | 1616.63 (H6N2-PROC) | 1014.25 (H5N1) |  |  |  |
|  |  |  |  |  |  |  |  |  |  |  |  |  | 968.38 (H2N2-PROC) | 366.13 (H1N1) | 1176.43 (H6N1) |  |  |  |
|  |  |  |  |  |  |  |  |  |  |  |  |  | 1130.42 (H3N2-PROC) | 528.07 (H2N1) | 1338.31 (H7N1) |  |  |  |
| O | 9.82 |  | 7 | 6 | 1 | 0 | 2737.09 | 1369.05 | 913.04 | n.d. | n.d. | 912.64 | 789.63 (N2F1-PROC) | 365.93 (H1N1) |  |  |  |  |
|  |  |  |  |  |  |  |  |  |  |  |  |  | 1642.63 (H4N3F1-PROC) | 528.25 (H2N1) |  |  |  |  |
|  |  |  |  |  |  |  |  |  |  |  |  |  | 1682.63 (H3N4F1-PROC) | 1987.75 (H6N5) |  |  |  |  |
|  |  |  |  |  |  |  |  |  |  |  |  |  | 2006.76 (H5N4F1-PROC) |  |  |  |  |  |
|  |  |  |  |  |  |  |  |  |  |  |  |  | 2372.75 (H6N5F1-PROC) |  |  |  |  |  |

**Table I.** Structural characterization of procainamide labelled KM12 human colorectal cancer cell line *N-*glycans from experiment 2. Structures for *N-*glycans are depicted following the Consortium for Functional Glycomics (CFG) notation: *N*-acetylglucosamine (N; blue square), fucose (F; red triangle), galactose (H; yellow circle), mannose (H; green circle), *N*-acetylneuraminic acid (S; purple diamond). Glycan compositions are given in the terms of hexose (H), *N*-acetylhexosamine (N), deoxyhexose (F), *N*-acetylneuraminic acid (S).

| Peak ID | Possible  structure | Composition | | | | Avg. GU |  | Human IgG *N*-glycans released with: | | | I.M. Data  *(inter-method data)* |
| --- | --- | --- | --- | --- | --- | --- | --- | --- | --- | --- | --- |
|  |  |  |  |  |  |  |  | IS *(n=3)* | PVDF *(n=3)* | N-Hy *(n=3)* |  |
|  |  | Hex | HexNAc | Fuc | Neu5Ac |  |  |  |  |  |  |
| A |  | 3 | 3 | 1 | 0 | 5.28 | Average % area | 1.15 | - | 0.50 | 0.55 |
|  |  |  |  |  |  |  | Standard deviation (SD) | 0.04 | - | 0.10 | - |
|  |  |  |  |  |  |  | Coefficient of variation (CV) | **3.83** | **-** | **19.74** | - |
| B |  | 3 | 4 | 0 | 0 | 5.40 | Average % area | 0.68 | 0.53 | 0.82 | 0.68 |
|  |  |  |  |  |  |  | Standard deviation (SD) | 0.06 | 0.07 | 0.10 | 0.15 |
|  |  |  |  |  |  |  | Coefficient of variation (CV) | **8.85** | **13.40** | **12.61** | 21.52 |
| C |  | 3 | 3 | 1 | 0 | 5.79 | Average % area | 17.07 | 17.38 | 17.05 | 17.16 |
|  |  |  |  |  |  |  | Standard deviation (SD) | 0.43 | 0.02 | 0.47 | 0.36 |
|  |  |  |  |  |  |  | Coefficient of variation (CV) | **2.54** | **0.10** | **2.75** | 2.09 |
| C |  | 3 | 4 | 1 | 0 | 5.79 |  |  |  |  |  |
| D |  | 5 | 2 | 0 | 0 | 6.05 | Average % area | 1.25 | 0.67 | 0.92 | 0.95 |
|  |  |  |  |  |  |  | Standard deviation (SD) | 0.08 | 0.13 | 0.15 | 0.27 |
|  |  |  |  |  |  |  | Coefficient of variation (CV) | **6.36** | **19.17** | **16.49** | 28.86 |
| D |  | 4 | 3 | 1 | 0 | 6.05 |  |  |  |  |  |
| E |  | 3 | 5 | 1 | 0 | 6.12 | Average % area | 3.33 | 3.40 | 3.62 | 3.45 |
|  |  |  |  |  |  |  | Standard deviation (SD) | 0.06 | 0.01 | 0.02 | 0.13 |
|  |  |  |  |  |  |  | Coefficient of variation (CV) | **1.74** | **0.35** | **0.48** | 3.89 |
| F |  | 4 | 4 | 0 | 0 | 6.19 | Average % area | 1.39 | 0.91 | 1.25 | 1.18 |
|  |  |  |  |  |  |  | Standard deviation (SD) | 0.04 | 0.04 | 0.15 | 0.23 |
|  |  |  |  |  |  |  | Coefficient of variation (CV) | **2.63** | **4.40** | **12.09** | 19.35 |
| G |  | 4 | 4 | 0 | 0 | 6.29 | Average % area | 0.62 | 0.58 | 0.69 | 0.63 |
|  |  |  |  |  |  |  | Standard deviation (SD) | 0.02 | 0.11 | 0.17 | 0.11 |
|  |  |  |  |  |  |  | Coefficient of variation (CV) | **4.02** | **18.98** | **24.39** | 17.61 |
| I |  | 4 | 4 | 1 | 0 | 6.56 | Average % area | 17.10 | 18.65 | 18.23 | 17.99 |
|  |  |  |  |  |  |  | Standard deviation (SD) | 0.40 | 0.16 | 0.41 | 0.75 |
|  |  |  |  |  |  |  | Coefficient of variation (CV) | **2.36** | **0.83** | **2.23** | 4.19 |
| J |  | 4 | 4 | 1 | 0 | 6.68 | Average % area | 7.93 | 7.58 | 9.67 | 8.39 |
|  |  |  |  |  |  |  | Standard deviation (SD) | 0.15 | 0.14 | 0.22 | 0.98 |
|  |  |  |  |  |  |  | Coefficient of variation (CV) | **1.92** | **1.81** | **2.24** | 11.67 |
| K |  | 4 | 5 | 1 | 0 | 6.80 | Average % area | 4.53 | 4.96 | 5.64 | 5.04 |
|  |  |  |  |  |  |  | Standard deviation (SD) | 0.09 | 0.14 | 0.10 | 0.49 |
|  |  |  |  |  |  |  | Coefficient of variation (CV) | **1.98** | **2.87** | **1.84** | 9.75 |
| L |  | 6 | 2 | 0 | 0 | 6.91 | Average % area | 1.29 | 1.14 | 1.49 | 1.31 |
|  |  |  |  |  |  |  | Standard deviation (SD) | 0.13 | 0.07 | 0.16 | 0.19 |
|  |  |  |  |  |  |  | Coefficient of variation (CV) | **10.23** | **6.44** | **10.90** | 14.57 |
| M |  | 5 | 4 | 0 | 0 | 7.08 | Average % area | 0.97 | 0.73 | 1.06 | 0.92 |
|  |  |  |  |  |  |  | Standard deviation (SD) | 0.13 | 0.06 | 0.13 | 0.18 |
|  |  |  |  |  |  |  | Coefficient of variation (CV) | **13.14** | **8.17** | **12.70** | 19.14 |
| O |  | 5 | 4 | 1 | 0 | 7.45 | Average % area | 12.93 | 14.53 | 14.85 | 14.10 |
|  |  |  |  |  |  |  | Standard deviation (SD) | 0.26 | 0.23 | 0.23 | 0.91 |
|  |  |  |  |  |  |  | Coefficient of variation (CV) | **2.01** | **1.62** | **1.56** | 6.48 |
| P |  | 5 | 5 | 1 | 0 | 7.60 | Average % area | 2.06 | 2.00 | 1.97 | 2.01 |
|  |  |  |  |  |  |  | Standard deviation (SD) | 0.05 | 0.05 | 0.03 | 0.05 |
|  |  |  |  |  |  |  | Coefficient of variation (CV) | **2.44** | **2.33** | **1.73** | 2.62 |
| Q |  | 4 | 4 | 1 | 1 | 7.68 | Average % area | 2.59 | 2.09 | 2.63 | 2.44 |
|  |  |  |  |  |  |  | Standard deviation (SD) | 0.09 | 0.04 | 0.09 | 0.27 |
|  |  |  |  |  |  |  | Coefficient of variation (CV) | **3.44** | **1.71** | **3.60** | 11.07 |
| S |  | 5 | 4 | 0 | 1 | 8.06 | Average % area | 2.13 | 1.89 | 1.42 | 1.82 |
|  |  |  |  |  |  |  | Standard deviation (SD) | 0.13 | 0.17 | 0.13 | 0.34 |
|  |  |  |  |  |  |  | Coefficient of variation (CV) | **6.06** | **9.02** | **9.45** | 18.60 |
| T |  | 5 | 5 | 0 | 1 | 8.32 | Average % area | 0.94 | 0.80 | 0.58 | 0.78 |
|  |  |  |  |  |  |  | Standard deviation (SD) | 0.15 | 0.07 | 0.13 | 0.19 |
|  |  |  |  |  |  |  | Coefficient of variation (CV) | **15.61** | **8.61** | **21.60** | 24.20 |
| U |  | 5 | 4 | 1 | 1 | 8.42 | Average % area | 9.61 | 10.08 | 9.66 | 9.78 |
|  |  |  |  |  |  |  | Standard deviation (SD) | 0.07 | 0.14 | 0.16 | 0.25 |
|  |  |  |  |  |  |  | Coefficient of variation (CV) | **0.78** | **1.35** | **1.62** | 2.55 |
| V |  | 5 | 5 | 1 | 1 | 8.65 | Average % area | 3.45 | 3.26 | 2.44 | 3.05 |
|  |  |  |  |  |  |  | Standard deviation (SD) | 0.24 | 0.20 | 0.08 | 0.49 |
|  |  |  |  |  |  |  | Coefficient of variation (CV) | **6.84** | **6.14** | **3.10** | 16.12 |
| W |  | 5 | 4 | 0 | 2 | 9.06 | Average % area | 1.29 | 1.13 | 0.80 | 1.07 |
|  |  |  |  |  |  |  | Standard deviation (SD) | 0.10 | 0.05 | 0.07 | 0.23 |
|  |  |  |  |  |  |  | Coefficient of variation (CV) | **7.53** | **4.00** | **8.43** | 21.04 |
| X | No MS/MS data detected | 5 | 5 | 0 | 2 | 9.20 | Average % area | 0.64 | 0.36 | 0.45 | 0.48 |
|  |  |  |  |  |  |  | Standard deviation (SD) | 0.12 | 0.02 | 0.09 | 0.14 |
|  |  |  |  |  |  |  | Coefficient of variation (CV) | **18.78** | **5.35** | **19.31** | 29.57 |
| Y |  | 5 | 4 | 1 | 2 | 9.41 | Average % area | 3.60 | 3.76 | 2.11 | 3.16 |
|  |  |  |  |  |  |  | Standard deviation (SD) | 0.04 | 0.13 | 0.04 | 0.79 |
|  |  |  |  |  |  |  | Coefficient of variation (CV) | **1.07** | **3.38** | **1.74** | 24.96 |
| Z |  | 5 | 5 | 1 | 2 | 9.52 | Average % area | 3.43 | 3.57 | 2.13 | 3.04 |
|  |  |  |  |  |  |  | Standard deviation (SD) | 0.02 | 0.07 | 0.05 | 0.69 |
|  |  |  |  |  |  |  | Coefficient of variation (CV) | **0.70** | **1.87** | **2.51** | 22.54 |

**Table J.** Glycan compositions and proposed structures, average GU values, average relative areas (average % area), standard deviations (SDs) and coefficients of variation (CVs) for the most abundant *N*-glycan structures detected in human IgG from experiment 1 and calculated after triplicate analysis. Structures for *N-*glycans are depicted following the Consortium for Functional Glycomics (CFG) notation: *N*-acetylglucosamine (N; blue square), fucose (F; red triangle), galactose (H; yellow circle), mannose (H; green circle), *N*-acetylneuraminic acid (S; purple diamond). Glycan compositions are given in the terms of hexose (H), *N*-acetylhexosamine (N), deoxyhexose (F), *N*-acetylneuraminic acid (S).

| Peak ID | Possible  structure | Composition | | | | Avg. GU |  | Human plasma *N*-glycans released with: | | | I.M. Data  *(inter-method data)* |
| --- | --- | --- | --- | --- | --- | --- | --- | --- | --- | --- | --- |
|  |  |  |  |  |  |  |  | IS *(n=3)* | PVDF *(n=3)* | N-Hy *(n=3)* |  |
|  |  | Hex | HexNAc | Fuc | Neu5Ac |  |  |  |  |  |  |
| B |  | 3 | 4 | 1 | 0 | 5.79 | Average % area | 3.79 | 3.32 | 5.13 | 4.08 |
|  |  |  |  |  |  |  | Standard deviation (SD) | 0.03 | 1.31 | 0.03 | 1.05 |
|  |  |  |  |  |  |  | Coefficient of variation (CV) | **0.71** | **39.54** | **0.67** | 25.62 |
| F |  | 4 | 4 | 1 | 0 | 6.56 | Average % area | 3.83 | 4.10 | 5.23 | 4.38 |
|  |  |  |  |  |  |  | Standard deviation (SD) | 0.01 | 1.67 | 0.05 | 1.06 |
|  |  |  |  |  |  |  | Coefficient of variation (CV) | **0.19** | **40.84** | **0.87** | 24.09 |
| G |  | 4 | 4 | 1 | 0 | 6.68 | Average % area | 1.63 | 1.87 | 2.48 | 1.99 |
|  |  |  |  |  |  |  | Standard deviation (SD) | 0.00 | 0.82 | 0.02 | 0.56 |
|  |  |  |  |  |  |  | Coefficient of variation (CV) | **0.04** | **43.86** | **0.93** | 28.12 |
| H |  | 4 | 5 | 1 | 0 | 6.80 | Average % area | 1.12 | 1.27 | 1.57 | 1.32 |
|  |  |  |  |  |  |  | Standard deviation (SD) | 0.01 | 0.43 | 0.08 | 0.30 |
|  |  |  |  |  |  |  | Coefficient of variation (CV) | **0.80** | **33.97** | **5.21** | 22.42 |
| H |  | 6 | 2 | 0 | 0 | 6.80 |  |  |  |  |  |
| H |  | 4 | 3 | 0 | 1 | 6.80 |  |  |  |  |  |
| L |  | 5 | 4 | 1 | 0 | 7.46 | Average % area | 2.77 | 4.11 | 4.33 | 3.74 |
|  |  |  |  |  |  |  | Standard deviation (SD) | 0.01 | 1.85 | 0.01 | 1.18 |
|  |  |  |  |  |  |  | Coefficient of variation (CV) | **0.44** | **44.98** | **0.12** | 31.50 |
| M |  | 4 | 4 | 1 | 1 | 7.61 | Average % area | 1.01 | 0.88 | 1.28 | 1.06 |
|  |  |  |  |  |  |  | Standard deviation (SD) | 0.01 | 0.24 | 0.03 | 0.22 |
|  |  |  |  |  |  |  | Coefficient of variation (CV) | **1.16** | **27.72** | **2.69** | 20.44 |
| P |  | 5 | 4 | 0 | 1 | 8.07 | Average % area | 8.97 | 5.52 | 11.03 | 8.52 |
|  |  |  |  |  |  |  | Standard deviation (SD) | 0.04 | 1.65 | 0.19 | 2.55 |
|  |  |  |  |  |  |  | Coefficient of variation (CV) | **0.45** | **29.82** | **1.74** | 29.98 |
| R |  | 5 | 4 | 1 | 1 | 8.43 | Average % area | 4.69 | 3.44 | 5.60 | 4.58 |
|  |  |  |  |  |  |  | Standard deviation (SD) | 0.08 | 0.72 | 0.04 | 1.00 |
|  |  |  |  |  |  |  | Coefficient of variation (CV) | **1.71** | **20.83** | **0.72** | 21.93 |
| S |  | 5 | 4 | 0 | 2 | 8.66 | Average % area | 6.34 | 4.56 | 5.73 | 5.54 |
|  |  |  |  |  |  |  | Standard deviation (SD) | 0.12 | 1.78 | 0.08 | 1.19 |
|  |  |  |  |  |  |  | Coefficient of variation (CV) | **1.93** | **39.02** | **1.31** | 21.45 |
| S |  | 5 | 5 | 1 | 1 | 8.66 |  |  |  |  |  |
| T |  | 5 | 4 | 1 | 2 | 9.06 | Average % area | 38.42 | 32.02 | 31.35 | 33.93 |
|  |  |  |  |  |  |  | Standard deviation (SD) | 0.02 | 11.92 | 0.10 | 6.85 |
|  |  |  |  |  |  |  | Coefficient of variation (CV) | **0.04** | **37.21** | **0.31** | 20.19 |
| T |  | 5 | 4 | 0 | 2 | 9.06 |  |  |  |  |  |
| U |  | 5 | 4 | 1 | 2 | 9.41 | Average % area | 6.91 | 6.62 | 6.13 | 6.56 |
|  |  |  |  |  |  |  | Standard deviation (SD) | 0.03 | 1.18 | 0.06 | 0.68 |
|  |  |  |  |  |  |  | Coefficient of variation (CV) | **0.37** | **17.86** | **1.05** | 10.43 |
| U |  | 9 | 2 | 0 | 0 | 9.41 |  |  |  |  |  |
| V |  | 5 | 5 | 1 | 2 | 9.53 | Average % area | 2.28 | 1.58 | 1.92 | 1.93 |
|  |  |  |  |  |  |  | Standard deviation (SD) | 0.01 | 0.27 | 0.04 | 0.34 |
|  |  |  |  |  |  |  | Coefficient of variation (CV) | **0.60** | **17.23** | **2.00** | 17.39 |
| W |  | 6 | 5 | 0 | 2 | 9.74 | Average % area | 1.01 | 1.12 | 1.58 | 1.24 |
|  |  |  |  |  |  |  | Standard deviation (SD) | 0.02 | 0.42 | 0.02 | 0.34 |
|  |  |  |  |  |  |  | Coefficient of variation (CV) | **1.66** | **37.72** | **1.06** | 27.18 |
| X |  | 6 | 5 | 0 | 2 | 10.12 | Average % area | 1.14 | 1.09 | 1.79 | 1.34 |
|  |  |  |  |  |  |  | Standard deviation (SD) | 0.01 | 0.07 | 0.01 | 0.34 |
|  |  |  |  |  |  |  | Coefficient of variation (CV) | **0.93** | **6.87** | **0.69** | 25.38 |
| Y |  | 6 | 5 | 0 | 3 | 10.31 | Average % area | 1.19 | 2.60 | 1.40 | 1.73 |
|  |  |  |  |  |  |  | Standard deviation (SD) | 0.02 | 0.94 | 0.01 | 0.81 |
|  |  |  |  |  |  |  | Coefficient of variation (CV) | **1.92** | **36.28** | **0.44** | 46.94 |
| Z |  | 6 | 5 | 0 | 3 | 10.71 | Average % area | 7.25 | 9.13 | 5.81 | 7.40 |
|  |  |  |  |  |  |  | Standard deviation (SD) | 0.05 | 5.34 | 0.05 | 3.04 |
|  |  |  |  |  |  |  | Coefficient of variation (CV) | **0.66** | **58.49** | **0.85** | 41.05 |
| AA |  | 6 | 5 | 0 | 3 | 11.13 | Average % area | 2.51 | 3.73 | 2.16 | 2.80 |
|  |  |  |  |  |  |  | Standard deviation (SD) | 0.04 | 2.93 | 0.01 | 1.63 |
|  |  |  |  |  |  |  | Coefficient of variation (CV) | **1.62** | **78.62** | **0.66** | 58.18 |
| AB |  | 6 | 5 | 1 | 3 | 11.25 | Average % area | 3.01 | 5.07 | 2.46 | 3.52 |
|  |  |  |  |  |  |  | Standard deviation (SD) | 0.03 | 4.05 | 0.03 | 2.35 |
|  |  |  |  |  |  |  | Coefficient of variation (CV) | **0.98** | **79.87** | **1.40** | 66.90 |
| AC | No MS/MS data detected | 7 | 6 | 0 | 4 | 11.97 | Average % area | 1.15 | 2.99 | 1.15 | 1.76 |
|  |  |  |  |  |  |  | Standard deviation (SD) | 0.03 | 3.32 | 0.03 | 1.90 |
|  |  |  |  |  |  |  | Coefficient of variation (CV) | **2.68** | **110.81** | **2.72** | 107.58 |

**Table K.** Glycan compositions and proposed structures, average GU values, average relative areas (average % area), standard deviations (SDs) and coefficients of variation (CVs) for the most abundant *N*-glycan structures detected in human plasma from experiment 1 and calculated after triplicate analysis. Structures for *N-*glycans are depicted following the Consortium for Functional Glycomics (CFG) notation: *N*-acetylglucosamine (N; blue square), fucose (F; red triangle), galactose (H; yellow circle), mannose (H; green circle), *N*-acetylneuraminic acid (S; purple diamond). Glycan compositions are given in the terms of hexose (H), *N*-acetylhexosamine (N), deoxyhexose (F), *N*-acetylneuraminic acid (S).

| Peak ID | Possible  structure | Composition | | | | Avg. GU |  | HT29 *N*-Glycans released with: | | | I.M. Data  *(inter-method data)* |
| --- | --- | --- | --- | --- | --- | --- | --- | --- | --- | --- | --- |
|  |  |  |  |  |  |  |  | IS *(n=3)* | PVDF *(n=3)* | N-Hy *(n=3)* |  |
|  |  | Hex | HexNAc | Fuc | Neu5Ac |  |  |  |  |  |  |
| E |  | 3 | 2 | 0 | 0 | 4.25 | Average % area | 4.32 | 4.16 | 17.55 | 8.68 |
|  |  |  |  |  |  |  | Standard deviation (SD) | 0.37 | 0.52 | 2.20 | 6.75 |
|  |  |  |  |  |  |  | Coefficient of variation (CV) | **8.67** | **12.58** | **12.51** | 77.85 |
| F |  | 3 | 2 | 1 | 0 | 4.69 | Average % area | 12.11 | 11.44 | 10.48 | 11.34 |
|  |  |  |  |  |  |  | Standard deviation (SD) | 0.63 | 0.67 | 0.41 | 0.87 |
|  |  |  |  |  |  |  | Coefficient of variation (CV) | **5.19** | **5.89** | **3.88** | 7.68 |
| H |  | 4 | 2 | 0 | 0 | 5.06 | Average % area | 1.00 | 0.91 | 19.25 | 7.05 |
|  |  |  |  |  |  |  | Standard deviation (SD) | 0.14 | 0.07 | 3.84 | 9.35 |
|  |  |  |  |  |  |  | Coefficient of variation (CV) | **13.65** | **7.99** | **19.92** | 132.50 |
| K |  | 5 | 1 | 0 | 0 | 5.60 | Average % area | 2.73 | 2.48 | 4.16 | 3.12 |
|  |  |  |  |  |  |  | Standard deviation (SD) | 0.50 | 0.47 | 0.87 | 0.96 |
|  |  |  |  |  |  |  | Coefficient of variation (CV) | **18.16** | **18.98** | **20.88** | 30.72 |
| M |  | 5 | 2 | 0 | 0 | 6.05 | Average % area | 10.54 | 10.58 | 9.39 | 10.17 |
|  |  |  |  |  |  |  | Standard deviation (SD) | 0.31 | 0.67 | 1.39 | 0.98 |
|  |  |  |  |  |  |  | Coefficient of variation (CV) | **2.97** | **6.37** | **14.83** | 9.67 |
| Q |  | 6 | 2 | 0 | 0 | 6.91 | Average % area | 16.48 | 16.24 | 11.31 | 14.68 |
|  |  |  |  |  |  |  | Standard deviation (SD) | 0.57 | 0.22 | 0.87 | 2.58 |
|  |  |  |  |  |  |  | Coefficient of variation (CV) | **3.44** | **1.38** | **7.72** | 17.59 |
| U |  | 7 | 2 | 0 | 0 | 7.78 | Average % area | 3.45 | 3.55 | 1.56 | 2.86 |
|  |  |  |  |  |  |  | Standard deviation (SD) | 0.39 | 0.21 | 0.22 | 1.00 |
|  |  |  |  |  |  |  | Coefficient of variation (CV) | **11.40** | **6.02** | **13.89** | 35.13 |
| V |  | 7 | 2 | 0 | 0 | 7.82 | Average % area | 10.05 | 9.83 | 6.80 | 8.89 |
|  |  |  |  |  |  |  | Standard deviation (SD) | 0.08 | 0.44 | 0.75 | 1.63 |
|  |  |  |  |  |  |  | Coefficient of variation (CV) | **0.83** | **4.43** | **10.95** | 18.31 |
| Y |  | 5 | 5 | 2 | 0 | 8.35 | Average % area | 4.01 | 4.46 | 1.51 | 3.33 |
|  |  |  |  |  |  |  | Standard deviation (SD) | 0.28 | 0.59 | 0.22 | 1.42 |
|  |  |  |  |  |  |  | Coefficient of variation (CV) | **6.92** | **13.30** | **14.50** | 42.74 |
| AA |  | 8 | 2 | 0 | 0 | 8.69 | Average % area | 22.36 | 22.67 | 11.54 | 18.86 |
|  |  |  |  |  |  |  | Standard deviation (SD) | 0.20 | 0.54 | 0.31 | 5.50 |
|  |  |  |  |  |  |  | Coefficient of variation (CV) | **0.88** | **2.40** | **2.70** | 29.16 |
| AD |  | 9 | 2 | 0 | 0 | 9.38 | Average % area | 12.95 | 13.68 | 6.46 | 11.03 |
|  |  |  |  |  |  |  | Standard deviation (SD) | 0.73 | 0.68 | 0.08 | 3.48 |
|  |  |  |  |  |  |  | Coefficient of variation (CV) | **5.67** | **4.99** | **1.19** | 31.56 |

**Table L.** Glycan compositions and proposed structures, average GU values, average relative areas (average % area), standard deviations (SDs) and coefficients of variation (CVs) for the most abundant *N*-glycan structures detected in HT29 human colorectal cancer cell line from experiment 1 and calculated after triplicate analysis. Structures for *N-*glycans are depicted following the Consortium for Functional Glycomics (CFG) notation: *N*-acetylglucosamine (N; blue square), fucose (F; red triangle), galactose (H; yellow circle), mannose (H; green circle), *N*-acetylneuraminic acid (S; purple diamond). Glycan compositions are given in the terms of hexose (H), *N*-acetylhexosamine (N), deoxyhexose (F), *N*-acetylneuraminic acid (S).

| Peak ID | Possible  structure | Composition | | | | Avg. GU |  | IgG samples released with: | | | I.M. Data  *(inter-method data)* |
| --- | --- | --- | --- | --- | --- | --- | --- | --- | --- | --- | --- |
|  |  |  |  |  |  |  |  | IS *(n=3)* | PVDF *(n=3)* | N-Hy *(n=3)* |  |
|  |  | Hex | HexNAc | Fuc | Neu5Ac |  |  |  |  |  |  |
| A |  | 3 | 3 | 1 | 0 | 5.28 | Average % area | 0.52 | 0.14 | 0.85 | 0.50 |
|  |  |  |  |  |  |  | Standard deviation (SD) | 0.05 | 0.01 | 0.06 | 0.31 |
|  |  |  |  |  |  |  | Coefficient of variation (CV) | **10.44** | **5.22** | **7.38** | 62.00 |
| B |  | 3 | 4 | 0 | 0 | 5.40 | Average % area | 0.63 | 0.56 | 1.33 | 0.84 |
|  |  |  |  |  |  |  | Standard deviation (SD) | 0.04 | 0.05 | 0.08 | 0.37 |
|  |  |  |  |  |  |  | Coefficient of variation (CV) | **5.92** | **8.83** | **5.79** | 44.09 |
| C |  | 3 | 3 | 1 | 0 | 5.79 | Average % area | 16.88 | 17.27 | 17.33 | 17.16 |
|  |  |  |  |  |  |  | Standard deviation (SD) | 0.09 | 0.23 | 0.26 | 0.28 |
|  |  |  |  |  |  |  | Coefficient of variation (CV) | **0.51** | **1.34** | **1.51** | 1.61 |
| C |  | 3 | 4 | 1 | 0 | 5.79 |  |  |  |  |  |
| D |  | 5 | 2 | 0 | 0 | 6.05 | Average % area | 0.30 | - | 0.82 | 0.38 |
|  |  |  |  |  |  |  | Standard deviation (SD) | 0.03 | - | 0.09 | 0.29 |
|  |  |  |  |  |  |  | Coefficient of variation (CV) | **10.14** | **-** | **10.91** | 77.91 |
| D |  | 4 | 3 | 1 | 0 | 6.05 |  |  | - |  |  |
| E |  | 3 | 5 | 1 | 0 | 6.12 | Average % area | 4.09 | 3.82 | 4.51 | 4.14 |
|  |  |  |  |  |  |  | Standard deviation (SD) | 0.27 | 0.30 | 0.07 | 0.36 |
|  |  |  |  |  |  |  | Coefficient of variation (CV) | **6.58** | **7.91** | **1.53** | 8.79 |
| F |  | 4 | 4 | 0 | 0 | 6.19 | Average % area | 1.25 | 1.00 | 1.36 | 1.20 |
|  |  |  |  |  |  |  | Standard deviation (SD) | 0.03 | 0.03 | 0.07 | 0.17 |
|  |  |  |  |  |  |  | Coefficient of variation (CV) | **2.42** | **2.75** | **4.91** | 13.80 |
| G |  | 4 | 4 | 0 | 0 | 6.29 | Average % area | 0.54 | 0.36 | 0.73 | 0.54 |
|  |  |  |  |  |  |  | Standard deviation (SD) | 0.02 | 0.05 | 0.02 | 0.16 |
|  |  |  |  |  |  |  | Coefficient of variation (CV) | **4.12** | **13.47** | **2.99** | 29.98 |
| H |  | 4 | 4 | 1 | 0 | 6.56 | Average % area | 16.92 | 18.35 | 17.95 | 17.74 |
|  |  |  |  |  |  |  | Standard deviation (SD) | 0.06 | 0.16 | 0.35 | 0.67 |
|  |  |  |  |  |  |  | Coefficient of variation (CV) | **0.37** | **0.87** | **1.93** | 3.77 |
| I |  | 4 | 4 | 1 | 0 | 6.68 | Average % area | 7.64 | 7.41 | 8.97 | 8.01 |
|  |  |  |  |  |  |  | Standard deviation (SD) | 0.05 | 0.11 | 0.10 | 0.73 |
|  |  |  |  |  |  |  | Coefficient of variation (CV) | **0.62** | **1.49** | **1.12** | 9.16 |
| J |  | 4 | 5 | 1 | 0 | 6.80 | Average % area | 4.71 | 4.55 | 5.39 | 4.88 |
|  |  |  |  |  |  |  | Standard deviation (SD) | 0.04 | 0.10 | 0.07 | 0.39 |
|  |  |  |  |  |  |  | Coefficient of variation (CV) | **0.75** | **2.17** | **1.38** | 7.95 |
| K |  | 6 | 2 | 0 | 0 | 6.91 | Average % area | 1.38 | 1.34 | 1.70 | 1.48 |
|  |  |  |  |  |  |  | Standard deviation (SD) | 0.00 | 0.04 | 0.12 | 0.18 |
|  |  |  |  |  |  |  | Coefficient of variation (CV) | **0.33** | **2.76** | **7.15** | 12.16 |
| L |  | 5 | 4 | 0 | 0 | 7.08 | Average % area | 1.20 | 1.08 | 1.18 | 1.15 |
|  |  |  |  |  |  |  | Standard deviation (SD) | 0.04 | 0.04 | 0.02 | 0.06 |
|  |  |  |  |  |  |  | Coefficient of variation (CV) | **3.47** | **3.57** | **1.90** | 5.46 |
| M |  | 5 | 4 | 1 | 0 | 7.45 | Average % area | 15.32 | 16.27 | 16.23 | 15.94 |
|  |  |  |  |  |  |  | Standard deviation (SD) | 0.07 | 0.24 | 0.27 | 0.50 |
|  |  |  |  |  |  |  | Coefficient of variation (CV) | **0.43** | **1.47** | **1.69** | 3.14 |
| N |  | 4 | 4 | 1 | 1 | 7.68 | Average % area | 1.63 | 1.47 | 2.06 | 1.72 |
|  |  |  |  |  |  |  | Standard deviation (SD) | 0.02 | 0.07 | 0.01 | 0.27 |
|  |  |  |  |  |  |  | Coefficient of variation (CV) | **1.12** | **4.84** | **0.39** | 15.44 |
| O |  | 4 | 4 | 0 | 1 | 8.06 | Average % area | 2.57 | 2.02 | 1.50 | 2.03 |
|  |  |  |  |  |  |  | Standard deviation (SD) | 0.07 | 0.23 | 0.07 | 0.48 |
|  |  |  |  |  |  |  | Coefficient of variation (CV) | **2.66** | **11.36** | **4.91** | 23.63 |
| P |  | 5 | 5 | 0 | 1 | 8.32 | Average % area | 1.01 | 0.72 | 0.71 | 0.81 |
|  |  |  |  |  |  |  | Standard deviation (SD) | 0.03 | 0.05 | 0.05 | 0.15 |
|  |  |  |  |  |  |  | Coefficient of variation (CV) | **2.69** | **7.37** | **7.11** | 18.62 |
| Q |  | 5 | 4 | 1 | 1 | 8.42 | Average % area | 10.17 | 10.97 | 9.79 | 10.31 |
|  |  |  |  |  |  |  | Standard deviation (SD) | 0.02 | 0.09 | 0.07 | 0.52 |
|  |  |  |  |  |  |  | Coefficient of variation (CV) | **0.19** | **0.86** | **0.67** | 5.08 |
| R |  | 5 | 5 | 1 | 1 | 8.65 | Average % area | 3.42 | 3.53 | 2.22 | 3.06 |
|  |  |  |  |  |  |  | Standard deviation (SD) | 0.04 | 0.32 | 0.06 | 0.65 |
|  |  |  |  |  |  |  | Coefficient of variation (CV) | **1.14** | **9.17** | **2.68** | 21.25 |
| S |  | 5 | 4 | 0 | 2 | 9.06 | Average % area | 1.75 | 1.57 | 0.94 | 1.42 |
|  |  |  |  |  |  |  | Standard deviation (SD) | 0.05 | 0.12 | 0.02 | 0.37 |
|  |  |  |  |  |  |  | Coefficient of variation (CV) | **2.88** | **7.94** | **2.41** | 26.30 |
| T | No MS/MS data detected | 5 | 5 | 0 | 2 | 9.20 | Average % area | 0.55 | 0.41 | 0.33 | 0.43 |
|  |  |  |  |  |  |  | Standard deviation (SD) | 0.02 | 0.08 | 0.03 | 0.10 |
|  |  |  |  |  |  |  | Coefficient of variation (CV) | **3.50** | **19.88** | **7.79** | 24.58 |

**Table M.** Glycan compositions and proposed structures, average GU values, average relative areas (average % area), standard deviations (SDs) and coefficients of variation (CVs) for the most abundant *N*-glycan structures detected in human IgG from experiment 2 and calculated after triplicate analysis. Structures for *N-*glycans are depicted following the Consortium for Functional Glycomics (CFG) notation: *N*-acetylglucosamine (N; blue square), fucose (F; red triangle), galactose (H; yellow circle), mannose (H; green circle), *N*-acetylneuraminic acid (S; purple diamond). Glycan compositions are given in the terms of hexose (H), *N*-acetylhexosamine (N), deoxyhexose (F), *N*-acetylneuraminic acid (S).

| Peak ID | Possible  structure | Composition | | | | Avg. GU |  | Plasma samples released with: | | | I.M. Data  *(inter-method data)* |
| --- | --- | --- | --- | --- | --- | --- | --- | --- | --- | --- | --- |
|  |  |  |  |  |  |  |  | IS *(n=3)* | PVDF *(n=3)* | N-Hy *(n=3)* |  |
|  |  | Hex | HexNAc | Fuc | Neu5Ac |  |  |  |  |  |  |
| A |  | 3 | 4 | 1 | 0 | 5.79 | Average % area | 3.94 | 4.17 | 6.80 | 4.97 |
|  |  |  |  |  |  |  | Standard deviation (SD) | 0.08 | 0.30 | 0.52 | 1.41 |
|  |  |  |  |  |  |  | Coefficient of variation (CV) | **1.96** | **7.31** | **7.65** | 28.38 |
| B |  | 4 | 4 | 1 | 0 | 6.56 | Average % area | 3.61 | 3.37 | 5.99 | 4.32 |
|  |  |  |  |  |  |  | Standard deviation (SD) | 0.07 | 0.16 | 0.29 | 1.26 |
|  |  |  |  |  |  |  | Coefficient of variation (CV) | **2.01** | **4.75** | **4.83** | 29.25 |
| C |  | 4 | 4 | 1 | 0 | 6.68 | Average % area | 1.75 | 1.48 | 2.91 | 2.05 |
|  |  |  |  |  |  |  | Standard deviation (SD) | 0.09 | 0.08 | 0.12 | 0.66 |
|  |  |  |  |  |  |  | Coefficient of variation (CV) | **5.35** | **5.42** | **4.15** | 32.43 |
| D |  | 4 | 5 | 1 | 0 | 6.80 | Average % area | 0.81 | 0.64 | 1.48 | 0.98 |
|  |  |  |  |  |  |  | Standard deviation (SD) | 0.09 | 0.07 | 0.10 | 0.39 |
|  |  |  |  |  |  |  | Coefficient of variation (CV) | **10.69** | **11.46** | **7.01** | 40.24 |
| D |  | 6 | 2 | 0 | 0 | 6.80 |  |  |  |  |  |
| D |  | 4 | 3 | 0 | 1 | 6.80 |  |  |  |  |  |
| E |  | 5 | 4 | 1 | 0 | 7.46 | Average % area | 3.39 | 3.07 | 5.52 | 3.99 |
|  |  |  |  |  |  |  | Standard deviation (SD) | 0.08 | 0.14 | 0.04 | 1.16 |
|  |  |  |  |  |  |  | Coefficient of variation (CV) | **2.22** | **4.49** | **0.68** | 29.02 |
| F |  | 4 | 4 | 1 | 1 | 7.61 | Average % area | 0.46 | 0.32 | 1.63 | 0.81 |
|  |  |  |  |  |  |  | Standard deviation (SD) | 0.04 | 0.04 | 0.12 | 0.63 |
|  |  |  |  |  |  |  | Coefficient of variation (CV) | **7.61** | **11.55** | **7.18** | 77.84 |
| G |  | 5 | 4 | 0 | 1 | 8.07 | Average % area | 8.91 | 8.85 | 9.73 | 9.16 |
|  |  |  |  |  |  |  | Standard deviation (SD) | 0.11 | 0.25 | 0.33 | 0.48 |
|  |  |  |  |  |  |  | Coefficient of variation (CV) | **1.19** | **2.82** | **3.40** | 5.19 |
| H |  | 5 | 4 | 1 | 1 | 8.43 | Average % area | 8.79 | 8.76 | 8.97 | 8.84 |
|  |  |  |  |  |  |  | Standard deviation (SD) | 0.08 | 0.76 | 0.15 | 0.40 |
|  |  |  |  |  |  |  | Coefficient of variation (CV) | **0.91** | **8.65** | **1.64** | 4.53 |
| I |  | 5 | 4 | 0 | 2 | 8.66 | Average % area | 1.91 | 1.52 | 1.84 | 1.87 |
|  |  |  |  |  |  |  | Standard deviation (SD) | 0.03 | 0.32 | 0.25 | 0.21 |
|  |  |  |  |  |  |  | Coefficient of variation (CV) | **1.34** | **17.20** | **13.52** | 10.98 |
| I |  | 5 | 5 | 1 | 1 | 8.66 |  |  |  |  |  |

| J |  | 5 | 4 | 1 | 2 | 9.06 | Average % area | 41.01 | 46.95 | 33.90 | 40.62 |
| --- | --- | --- | --- | --- | --- | --- | --- | --- | --- | --- | --- |
|  |  |  |  |  |  |  | Standard deviation (SD) | 0.29 | 1.61 | 0.87 | 5.74 |
|  |  |  |  |  |  |  | Coefficient of variation (CV) | **0.70** | **3.43** | **2.56** | 14.12 |
| J |  | 5 | 4 | 0 | 2 | 9.06 |  |  |  |  |  |
| K |  | 5 | 4 | 1 | 2 | 9.41 | Average % area | 6.31 | 5.70 | 4.84 | 5.62 |
|  |  |  |  |  |  |  | Standard deviation (SD) | 0.07 | 0.17 | 0.14 | 0.65 |
|  |  |  |  |  |  |  | Coefficient of variation (CV) | **1.18** | **3.00** | **2.82** | 11.57 |
| K |  | 9 | 2 | 0 | 0 | 9.41 |  |  |  |  |  |
| L |  | 5 | 5 | 1 | 2 | 9.53 | Average % area | 2.31 | 1.90 | 1.87 | 2.03 |
|  |  |  |  |  |  |  | Standard deviation (SD) | 0.03 | 0.14 | 0.14 | 0.23 |
|  |  |  |  |  |  |  | Coefficient of variation (CV) | **1.24** | **7.40** | **7.33** | 11.50 |
| M |  | 6 | 5 | 0 | 2 | 9.74 | Average % area | 0.88 | 0.49 | 0.80 | 0.72 |
|  |  |  |  |  |  |  | Standard deviation (SD) | 0.01 | 0.02 | 0.04 | 0.18 |
|  |  |  |  |  |  |  | Coefficient of variation (CV) | **1.15** | **5.06** | **5.25** | 24.63 |
| N |  | 6 | 5 | 0 | 2 | 10.12 | Average % area | 1.14 | 1.08 | 1.38 | 1.20 |
|  |  |  |  |  |  |  | Standard deviation (SD) | 0.033 | 0.30 | 0.17 | 0.22 |
|  |  |  |  |  |  |  | Coefficient of variation (CV) | **2.64** | **27.80** | **12.61** | 18.55 |
| O |  | 6 | 5 | 0 | 3 | 10.31 | Average % area | 1.56 | 1.20 | 1.65 | 1.47 |
|  |  |  |  |  |  |  | Standard deviation (SD) | 0.02 | 0.13 | 0.20 | 0.24 |
|  |  |  |  |  |  |  | Coefficient of variation (CV) | **1.08** | **11.20** | **12.39** | 16.22 |
| P |  | 6 | 5 | 0 | 3 | 10.71 | Average % area | 7.04 | 5.72 | 5.56 | 6.14 |
|  |  |  |  |  |  |  | Standard deviation (SD) | 0.07 | 0.23 | 0.05 | 0.69 |
|  |  |  |  |  |  |  | Coefficient of variation (CV) | **1.07** | **4.08** | **0.86** | 11.20 |
| Q |  | 6 | 5 | 0 | 3 | 11.13 | Average % area | 2.55 | 1.80 | 2.17 | 2.17 |
|  |  |  |  |  |  |  | Standard deviation (SD) | 0.05 | 0.08 | 0.05 | 0.33 |
|  |  |  |  |  |  |  | Coefficient of variation (CV) | **1.78** | **4.58** | **2.40** | 15.10 |
| R |  | 6 | 5 | 1 | 3 | 11.25 | Average % area | 3.65 | 2.63 | 2.87 | 3.05 |
|  |  |  |  |  |  |  | Standard deviation (SD) | 0.03 | 0.20 | 0.06 | 0.48 |
|  |  |  |  |  |  |  | Coefficient of variation (CV) | **0.74** | **7.70** | **2.22** | 15.62 |

**Table N.** Glycan compositions and proposed structures, average GU values, average relative areas (average % area), standard deviations (SDs) and coefficients of variation (CVs) for the most abundant *N*-glycan structures detected in human plasma from experiment 2 and calculated after triplicate analysis. Structures for *N-*glycans are depicted following the Consortium for Functional Glycomics (CFG) notation: *N*-acetylglucosamine (N; blue square), fucose (F; red triangle), galactose (H; yellow circle), mannose (H; green circle), *N*-acetylneuraminic acid (S; purple diamond). Glycan compositions are given in the terms of hexose (H), *N*-acetylhexosamine (N), deoxyhexose (F), *N*-acetylneuraminic acid (S).

| Peak ID | Possible  structure | Composition | | | | Avg. GU |  | HT29 samples released with: | | | I.M. Data  *(inter-method data)* |
| --- | --- | --- | --- | --- | --- | --- | --- | --- | --- | --- | --- |
|  |  |  |  |  |  |  |  | IS *(n=3)* | PVDF *(n=3)* | N-Hy *(n=3)* |  |
|  |  | Hex | HexNAc | Fuc | Neu5Ac |  |  |  |  |  |  |
| A |  | 3 | 1 | 0 | 0 | 3.61 | Average % area | 5.06 | - | - | - |
|  |  |  |  |  |  |  | Standard deviation (SD) | 0.08 | - | - | - |
|  |  |  |  |  |  |  | Coefficient of variation (CV) | **1.51** | **-** | **-** | - |
| B |  | 2 | 2 | 1 | 0 | 3.72 | Average % area | 0.83 | - | 0.62 | - |
|  |  |  |  |  |  |  | Standard deviation (SD) | 0.05 | - | 0.04 | - |
|  |  |  |  |  |  |  | Coefficient of variation (CV) | **6.05** | **-** | **6.10** | - |
| C |  | 3 | 1 | 0 | 0 | 3.73 | Average % area | 3.13 | - | 3.03 | - |
|  |  |  |  |  |  |  | Standard deviation (SD) | 0.04 | - | 0.37 | - |
|  |  |  |  |  |  |  | Coefficient of variation (CV) | **1.29** | **-** | **12.12** | - |
| D |  | 3 | 2 | 0 | 0 | 4.24 | Average % area | 1.83 | 4.14 | 7.19 | 4.39 |
|  |  |  |  |  |  |  | Standard deviation (SD) | 0.03 | 1.07 | 0.30 | 2.39 |
|  |  |  |  |  |  |  | Coefficient of variation (CV) | **1.43** | **25.81** | **4.17** | 54.54 |
| E |  | 4 | 1 | 0 | 0 | 4.55 | Average % area | 2.23 | - | 4.89 | - |
|  |  |  |  |  |  |  | Standard deviation (SD) | 0.15 | - | 0.28 | - |
|  |  |  |  |  |  |  | Coefficient of variation (CV) | **6.59** | **-** | **5.81** | - |
| F | - | 3 | 2 | 1 | 0 | 4.66 | Average % area | 10.66 | 7.04 | 22.33 | 13.35 |
|  |  |  |  |  |  |  | Standard deviation (SD) | 0.45 | 0.83 | 1.36 | 6.97 |
|  |  |  |  |  |  |  | Coefficient of variation (CV) | **4.26** | **11.84** | **6.08** | 52.23 |
| F |  | 4 | 1 | 0 | 0 | 4.66 | Average % area |  |  |  |  |
|  |  |  |  |  |  |  | Standard deviation (SD) |  |  |  |  |
|  |  |  |  |  |  |  | Coefficient of variation (CV) |  |  |  |  |
| G |  | 5 | 1 | 0 | 0 | 5.57 | Average % area | 10.12 | 7.42 | 7.32 | 8.28 |
|  |  |  |  |  |  |  | Standard deviation (SD) | 0.33 | 0.81 | 1.49 | 1.62 |
|  |  |  |  |  |  |  | Coefficient of variation (CV) | **3.22** | **10.98** | **20.37** | 19.61 |
| H |  | 3 | 3 | 1 | 0 | 5.88 | Average % area | 0.73 | - | 3.10 | - |
|  |  |  |  |  |  |  | Standard deviation (SD) | 0.04 | - | 0.33 | - |
|  |  |  |  |  |  |  | Coefficient of variation (CV) | **5.07** | **-** | **10.73** | - |
| I |  | 5 | 2 | 0 | 0 | 6.09 | Average % area | 5.40 | 9.74 | 5.24 | 6.79 |
|  |  |  |  |  |  |  | Standard deviation (SD) | 0.06 | 1.42 | 0.29 | 2.33 |
|  |  |  |  |  |  |  | Coefficient of variation (CV) | **1.05** | **14.59** | **5.56** | 34.25 |
| I |  | 4 | 3 | 1 | 0 | 6.09 | Average % area |  |  |  |  |
|  |  |  |  |  |  |  | Standard deviation (SD) |  |  |  |  |
|  |  |  |  |  |  |  | Coefficient of variation (CV) |  |  |  |  |
| J |  | 6 | 1 | 0 | 0 | 6.21 | Average % area | 0.75 | - | 0.76 | - |
|  |  |  |  |  |  |  | Standard deviation (SD) | 0.03 | - | 0.12 | - |
|  |  |  |  |  |  |  | Coefficient of variation (CV) | **3.46** | **-** | **15.57** | - |
| K |  | 4 | 3 | 1 | 0 | 6.41 | Average % area | 2.68 | 5.83 | 2.27 | 3.60 |
|  |  |  |  |  |  |  | Standard deviation (SD) | 0.06 | 0.46 | 0.08 | 1.70 |
|  |  |  |  |  |  |  | Coefficient of variation (CV) | **2.06** | **7.87** | **3.32** | 47.32 |

| L |  | 6 | 2 | 0 | 0 | 6.81 | Average % area | 9.43 | 11.17 | 8.03 | 9.55 |
| --- | --- | --- | --- | --- | --- | --- | --- | --- | --- | --- | --- |
|  |  |  |  |  |  |  | Standard deviation (SD) | 0.05 | 2.97 | 0.22 | 2.02 |
|  |  |  |  |  |  |  | Coefficient of variation (CV) | **0.52** | **26.57** | **2.79** | 21.12 |
| M |  | 7 | 1 | 0 | 0 | 7.11 | Average % area | 2.33 | 3.53 | 0.94 | 2.27 |
|  |  |  |  |  |  |  | Standard deviation (SD) | 0.02 | 0.98 | 0.01 | 1.23 |
|  |  |  |  |  |  |  | Coefficient of variation (CV) | **1.05** | **27.69** | **1.44** | 54.06 |
| N |  | 6 | 3 | 0 | 0 | 7.41 | Average % area | 2.21 | 7.51 | 1.49 | 3.74 |
|  |  |  |  |  |  |  | Standard deviation (SD) | 0.07 | 0.89 | 0.13 | 2.88 |
|  |  |  |  |  |  |  | Coefficient of variation (CV) | **2.98** | **11.82** | **8.61** | 77.05 |
| O |  | 5 | 5 | 1 | 0 | 7.42 | Average % area | 0.90 | - | 0.47 | - |
|  |  |  |  |  |  |  | Standard deviation (SD) | 0.02 | - | 0.05 | - |
|  |  |  |  |  |  |  | Coefficient of variation (CV) | **2.00** | **-** | **10.99** | - |
| P |  | 7 | 2 | 0 | 0 | 7.82 | Average % area | 6.76 | 9.85 | 4.19 | 6.93 |
|  |  |  |  |  |  |  | Standard deviation (SD) | 0.08 | 1.67 | 0.16 | 2.60 |
|  |  |  |  |  |  |  | Coefficient of variation (CV) | **1.14** | **16.97** | **3.88** | 37.45 |
| Q |  | 5 | 4 | 0 | 2 | 8.12 | Average % area | 3.49 | - | 2.31 | - |
|  |  |  |  |  |  |  | Standard deviation (SD) | 0.08 | - | 0.09 | - |
|  |  |  |  |  |  |  | Coefficient of variation (CV) | **2.16** | **-** | **3.73** | - |
| R |  | 4 | 5 | 3 | 0 | 8.52 | Average % area | 2.24 | 5.19 | 2.53 | 3.32 |
|  |  |  |  |  |  |  | Standard deviation (SD) | 0.06 | 5.05 | 0.23 | 2.89 |
|  |  |  |  |  |  |  | Coefficient of variation (CV) | **2.77** | **97.16** | **9.19** | 87.09 |
| S |  | 8 | 2 | 0 | 0 | 8.62 | Average % area | 13.38 | 15.94 | 9.31 | 12.88 |
|  |  |  |  |  |  |  | Standard deviation (SD) | 0.29 | 4.08 | 0.64 | 3.56 |
|  |  |  |  |  |  |  | Coefficient of variation (CV) | **2.16** | **25.60** | **6.86** | 27.64 |
| T |  | 5 | 4 | 3 | 0 | 8.92 | Average % area | 3.21 | 3.90 | 2.27 | 3.13 |
|  |  |  |  |  |  |  | Standard deviation (SD) | 0.07 | 0.64 | 0.20 | 0.79 |
|  |  |  |  |  |  |  | Coefficient of variation (CV) | **2.32** | **16.40** | **8.78** | 25.21 |
| U |  | 5 | 5 | 3 | 0 | 9.02 | Average % area | 2.58 | - | 2.42 | - |
|  |  |  |  |  |  |  | Standard deviation (SD) | 0.03 | - | 0.25 | - |
|  |  |  |  |  |  |  | Coefficient of variation (CV) | **1.08** | **-** | **10.39** | - |
| V |  | 9 | 2 | 0 | 0 | 9.32 | Average % area | 10.03 | 8.73 | 8.90 | 9.22 |
|  |  |  |  |  |  |  | Standard deviation (SD) | 0.28 | 1.36 | 0.60 | 0.98 |
|  |  |  |  |  |  |  | Coefficient of variation (CV) | **2.79** | **15.64** | **6.70** | 10.59 |

**Table O.** Glycan compositions and proposed structures, average GU values, average relative areas (average % area), standard deviations (SDs) and coefficients of variation (CVs) for the most abundant *N*-glycan structures detected in HT29 human colorectal cancer cell line from experiment 2 and calculated after triplicate analysis. Structures for *N-*glycans are depicted following the Consortium for Functional Glycomics (CFG) notation: *N*-acetylglucosamine (N; blue square), fucose (F; red triangle), galactose (H; yellow circle), mannose (H; green circle), *N*-acetylneuraminic acid (S; purple diamond). Glycan compositions are given in the terms of hexose (H), *N*-acetylhexosamine (N), deoxyhexose (F), *N*-acetylneuraminic acid (S).

| Peak ID | Possible  structure | Composition | | | | Avg. GU |  | HCT15 samples released with: | | | I.M. Data  *(inter-method data)* |
| --- | --- | --- | --- | --- | --- | --- | --- | --- | --- | --- | --- |
|  |  |  |  |  |  |  |  | IS *(n=3)* | PVDF *(n=3)* | N-Hy *(n=3)* |  |
|  |  | Hex | HexNAc | Fuc | Neu5Ac |  |  |  |  |  |  |
| A |  | 2 | 2 | 1 | 0 | 3.72 | Average % area | 1.93 | - | 2.24 | - |
|  |  |  |  |  |  |  | Standard deviation (SD) | 0.28 | - | 0.45 | - |
|  |  |  |  |  |  |  | Coefficient of variation (CV) | **14.71** | **-** | **20.19** | - |
| A |  | 3 | 1 | 0 | 0 | 3.72 | Average % area |  |  |  |  |
|  |  |  |  |  |  |  | Standard deviation (SD) |  |  |  |  |
|  |  |  |  |  |  |  | Coefficient of variation (CV) |  |  |  |  |
| B |  | 3 | 1 | 0 | 0 | 3.83 | Average % area | 0.91 | - | 0.66 | - |
|  |  |  |  |  |  |  | Standard deviation (SD) | 0.12 | - | 0.12 | - |
|  |  |  |  |  |  |  | Coefficient of variation (CV) | **13.40** | **-** | **18.63** | - |
| C |  | 3 | 2 | 0 | 0 | 4.24 | Average % area | 1.05 | 3.78 | 15.25 | 6.69 |
|  |  |  |  |  |  |  | Standard deviation (SD) | 0.00 | 0.56 | 0.77 | 6.54 |
|  |  |  |  |  |  |  | Coefficient of variation (CV) | **0.19** | **14.76** | **5.08** | 97.70 |
| D |  | 4 | 1 | 0 | 0 | 4.75 | Average % area | 7.15 | 6.91 | 18.63 | 10.90 |
|  |  |  |  |  |  |  | Standard deviation (SD) | 0.13 | 0.27 | 2.08 | 5.90 |
|  |  |  |  |  |  |  | Coefficient of variation (CV) | **1.78** | **3.95** | **11.19** | 54.10 |
| D |  | 3 | 2 | 1 | 0 | 4.75 | Average % area |  |  |  |  |
|  |  |  |  |  |  |  | Standard deviation (SD) |  |  |  |  |
|  |  |  |  |  |  |  | Coefficient of variation (CV) |  |  |  |  |
| E | - | 4 | 2 | 0 | 0 | 5.16 | Average % area | 0.36 | - | 1.47 | - |
|  |  |  |  |  |  |  | Standard deviation (SD) | 0.03 | - | 0.36 | - |
|  |  |  |  |  |  |  | Coefficient of variation (CV) | **9.23** | **-** | **24.77** | - |
| F |  | 3 | 3 | 1 | 0 | 5.27 | Average % area | 0.76 | - | 3.76 | - |
|  |  |  |  |  |  |  | Standard deviation (SD) | 0.03 | - | 0.74 | - |
|  |  |  |  |  |  |  | Coefficient of variation (CV) | **4.34** | **-** | **19.71** | - |
| G |  | 5 | 1 | 0 | 0 | 5.61 | Average % area | 13.70 | - | 8.53 | - |
|  |  |  |  |  |  |  | Standard deviation (SD) | 0.38 | - | 1.06 | - |
|  |  |  |  |  |  |  | Coefficient of variation (CV) | **2.74** | **-** | **12.43** | - |
| H |  | 5 | 2 | 0 | 0 | 6.01 | Average % area | 4.43 | 11.23 | 3.74 | 6.47 |
|  |  |  |  |  |  |  | Standard deviation (SD) | 0.27 | 1.62 | 0.31 | 3.68 |
|  |  |  |  |  |  |  | Coefficient of variation (CV) | **6.20** | **14.47** | **8.29** | 56.90 |
| I |  | 6 | 1 | 0 | 0 | 6.42 | Average % area | 3.05 | - | 2.92 | - |
|  |  |  |  |  |  |  | Standard deviation (SD) | 0.22 | - | 0.18 | - |
|  |  |  |  |  |  |  | Coefficient of variation (CV) | **7.28** | **-** | **6.05** | - |
| J |  | 6 | 2 | 0 | 0 | 6.92 | Average % area | 10.58 | 14.60 | 8.11 | 11.10 |
|  |  |  |  |  |  |  | Standard deviation (SD) | 0.25 | 0.55 | 0.61 | 2.87 |
|  |  |  |  |  |  |  | Coefficient of variation (CV) | **2.38** | **3.79** | **7.47** | 25.83 |
| K |  | 7 | 1 | 0 | 0 | 7.12 | Average % area | 2.16 | - | 0.96 | - |
|  |  |  |  |  |  |  | Standard deviation (SD) | 0.18 | - | 0.11 | - |
|  |  |  |  |  |  |  | Coefficient of variation (CV) | **8.28** | **-** | **11.43** | - |

| L |  | 5 | 4 | 1 | 0 | 7.43 | Average % area | 2.20 | 6.47 | 1.71 | 3.46 |
| --- | --- | --- | --- | --- | --- | --- | --- | --- | --- | --- | --- |
|  |  |  |  |  |  |  | Standard deviation (SD) | 0.07 | 2.55 | 0.07 | 2.60 |
|  |  |  |  |  |  |  | Coefficient of variation (CV) | **3.24** | **39.42** | **3.93** | 75.23 |
| M |  | 7 | 1 | 0 | 0 | 7.53 | Average % area | 1.69 | - | 0.93 | - |
|  |  |  |  |  |  |  | Standard deviation (SD) | 0.12 | - | 0.14 | - |
|  |  |  |  |  |  |  | Coefficient of variation (CV) | **6.81** | **-** | **15.40** | - |
| N |  | 7 | 2 | 0 | 0 | 7.82 | Average % area | 5.25 | 8.15 | 3.50 | 5.63 |
|  |  |  |  |  |  |  | Standard deviation (SD) | 0.47 | 0.89 | 0.13 | 2.10 |
|  |  |  |  |  |  |  | Coefficient of variation (CV) | **8.91** | **10.91** | **3.83** | 37.23 |
| O |  | 8 | 1 | 0 | 0 | 8.13 | Average % area | 3.24 | 3.27 | 1.94 | 2.82 |
|  |  |  |  |  |  |  | Standard deviation (SD) | 0.29 | 0.79 | 0.48 | 0.82 |
|  |  |  |  |  |  |  | Coefficient of variation (CV) | **9.04** | **24.25** | **24.96** | 29.05 |
| P |  | 8 | 1 | 0 | 0 | 8.43 | Average % area | 2.42 | - | 1.75 | - |
|  |  |  |  |  |  |  | Standard deviation (SD) | 0.25 | - | 0.56 | - |
|  |  |  |  |  |  |  | Coefficient of variation (CV) | **10.30** | **-** | **32.20** | - |
| Q |  | 5 | 4 | 1 | 1 | 8.54 | Average % area | 1.96 | - | 1.87 | - |
|  |  |  |  |  |  |  | Standard deviation (SD) | 0.20 | - | 0.16 | - |
|  |  |  |  |  |  |  | Coefficient of variation (CV) | **10.27** | **-** | **8.69** | - |
| R |  | 8 | 2 | 0 | 0 | 8.74 | Average % area | 15.28 | 19.54 | 9.44 | 14.75 |
|  |  |  |  |  |  |  | Standard deviation (SD) | 0.36 | 3.91 | 0.30 | 4.81 |
|  |  |  |  |  |  |  | Coefficient of variation (CV) | **2.33** | **20.03** | **3.18** | 32.61 |
| S |  | 6 | 5 | 0 | 1 | 8.84 | Average % area | 1.38 | - | 0.56 | - |
|  |  |  |  |  |  |  | Standard deviation (SD) | 0.19 | - | 0.17 | - |
|  |  |  |  |  |  |  | Coefficient of variation (CV) | **13.93** | **-** | **31.09** | - |
| T |  | 5 | 4 | 1 | 1 | 8.94 | Average % area | 1.60 | - | 0.87 | - |
|  |  |  |  |  |  |  | Standard deviation (SD) | 0.30 | - | 0.24 | - |
|  |  |  |  |  |  |  | Coefficient of variation (CV) | **18.51** | **-** | **27.99** | - |
| U |  | 6 | 5 | 3 | 0 | 9.14 | Average % area | 3.85 | - | 2.09 | - |
|  |  |  |  |  |  |  | Standard deviation (SD) | 0.22 | - | 0.38 | - |
|  |  |  |  |  |  |  | Coefficient of variation (CV) | **5.71** | **-** | **18.11** | - |
| V |  | 9 | 2 | 0 | 0 | 9.34 | Average % area | 15.03 | 26.06 | 9.09 | 16.73 |
|  |  |  |  |  |  |  | Standard deviation (SD) | 0.25 | 1.49 | 0.20 | 7.50 |
|  |  |  |  |  |  |  | Coefficient of variation (CV) | **1.65** | **5.71** | **2.19** | 44.82 |

**Table P.** Glycan compositions and proposed structures, average GU values, average relative areas (average % area), standard deviations (SDs) and coefficients of variation (CVs) for the most abundant *N*-glycan structures detected in HCT15 human colorectal cancer cell line from experiment 2 and calculated after triplicate analysis. Structures for *N-*glycans are depicted following the Consortium for Functional Glycomics (CFG) notation: *N*-acetylglucosamine (N; blue square), fucose (F; red triangle), galactose (H; yellow circle), mannose (H; green circle), *N*-acetylneuraminic acid (S; purple diamond). Glycan compositions are given in the terms of hexose (H), *N*-acetylhexosamine (N), deoxyhexose (F), *N*-acetylneuraminic acid (S).

| Peak ID | Possible  structure | Composition | | | | Avg. GU |  | HCT116 samples released with: | | | I.M. Data  *(inter-method data)* |
| --- | --- | --- | --- | --- | --- | --- | --- | --- | --- | --- | --- |
|  |  |  |  |  |  |  |  | IS *(n=3)* | PVDF *(n=3)* | N-Hy *(n=3)* |  |
|  |  | Hex | HexNAc | Fuc | Neu5Ac |  |  |  |  |  |  |
| A |  | 3 | 1 | 0 | 0 | 3.71 | Average % area | 2.62 | - | 2.43 | - |
|  |  |  |  |  |  |  | Standard deviation (SD) | 0.11 | - | 0.27 | - |
|  |  |  |  |  |  |  | Coefficient of variation (CV) | **4.04** | **-** | **11.14** | - |
| B |  | 3 | 2 | 0 | 0 | 4.22 | Average % area | 5.14 | - | 26.94 | - |
|  |  |  |  |  |  |  | Standard deviation (SD) | 0.40 | - | 4.11 | - |
|  |  |  |  |  |  |  | Coefficient of variation (CV) | **7.78** | **-** | **15.24** | - |
| C |  | 5 | 2 | 0 | 0 | 6.03 | Average % area | 6.28 | 11.14 | 7.75 | 8.39 |
|  |  |  |  |  |  |  | Standard deviation (SD) | 0.13 | 1.10 | 0.81 | 2.26 |
|  |  |  |  |  |  |  | Coefficient of variation (CV) | **2.00** | **9.83** | **10.44** | 26.99 |
| D | - | 5 | 3 | 0 | 0 | 6.54 | Average % area | 4.01 | 9.39 | 3.77 | 5.72 |
|  |  |  |  |  |  |  | Standard deviation (SD) | 0.22 | 2.22 | 0.14 | 2.97 |
|  |  |  |  |  |  |  | Coefficient of variation (CV) | **5.39** | **23.67** | **3.60** | 51.89 |
| E |  | 4 | 3 | 0 | 1 | 6.65 | Average % area | 1.27 | 4.15 | 1.56 | 2.33 |
|  |  |  |  |  |  |  | Standard deviation (SD) | 0.12 | 0.98 | 0.14 | 1.46 |
|  |  |  |  |  |  |  | Coefficient of variation (CV) | **9.59** | **23.59** | **8.69** | 62.64 |
| F |  | 6 | 2 | 0 | 0 | 6.96 | Average % area | 8.99 | 8.79 | 8.49 | 8.76 |
|  |  |  |  |  |  |  | Standard deviation (SD) | 0.14 | 0.85 | 1.00 | 0.69 |
|  |  |  |  |  |  |  | Coefficient of variation (CV) | **1.57** | **9.62** | **11.78** | 7.92 |
| G |  | 5 | 4 | 0 | 0 | 7.07 | Average % area | 1.76 | 6.10 | 1.39 | 3.08 |
|  |  |  |  |  |  |  | Standard deviation (SD) | 0.05 | 1.04 | 0.21 | 2.33 |
|  |  |  |  |  |  |  | Coefficient of variation (CV) | **2.71** | **17.02** | **15.13** | 75.57 |
| H |  | 5 | 3 | 0 | 1 | 7.38 | Average % area | 1.67 | - | 2.01 | - |
|  |  |  |  |  |  |  | Standard deviation (SD) | 0.08 | - | 0.24 | - |
|  |  |  |  |  |  |  | Coefficient of variation (CV) | **4.81** | **-** | **11.72** | - |
| I |  | 6 | 3 | 0 | 0 | 7.49 | Average % area | 4.39 | 9.54 | 3.05 | 5.66 |
|  |  |  |  |  |  |  | Standard deviation (SD) | 0.17 | 1.23 | 0.25 | 3.03 |
|  |  |  |  |  |  |  | Coefficient of variation (CV) | **3.85** | **12.86** | **8.06** | 53.55 |
| I |  | 5 | 4 | 0 | 1 | 7.49 | Average % area |  |  |  |  |
|  |  |  |  |  |  |  | Standard deviation (SD) |  |  |  |  |
|  |  |  |  |  |  |  | Coefficient of variation (CV) |  |  |  |  |
| J |  | 7 | 2 | 0 | 0 | 7.71 | Average % area | 10.34 | 16.49 | 8.08 | 11.64 |
|  |  |  |  |  |  |  | Standard deviation (SD) | 0.10 | 1.73 | 0.72 | 3.89 |
|  |  |  |  |  |  |  | Coefficient of variation (CV) | **0.94** | **10.51** | **8.92** | 33.38 |
| K |  | 5 | 4 | 0 | 2 | 8.11 | Average % area | 7.17 | 9.90 | 4.72 | 7.26 |
|  |  |  |  |  |  |  | Standard deviation (SD) | 0.19 | 2.82 | 0.60 | 2.67 |
|  |  |  |  |  |  |  | Coefficient of variation (CV) | **2.60** | **28.50** | **12.65** | 36.74 |
| L |  | 8 | 2 | 0 | 0 | 8.61 | Average % area | 17.84 | 12.19 | 11.39 | 13.81 |
|  |  |  |  |  |  |  | Standard deviation (SD) | 0.31 | 2.14 | 1.39 | 3.31 |
|  |  |  |  |  |  |  | Coefficient of variation (CV) | **1.73** | **17.58** | **12.22** | 23.96 |

| M |  | 6 | 5 | 0 | 1 | 8.81 | Average % area | 2.12 | 0.94 | 0.90 | 1.32 |
| --- | --- | --- | --- | --- | --- | --- | --- | --- | --- | --- | --- |
|  |  |  |  |  |  |  | Standard deviation (SD) | 0.32 | 0.27 | 0.31 | 0.65 |
|  |  |  |  |  |  |  | Coefficient of variation (CV) | **14.95** | **28.73** | **34.04** | 49.53 |
| N |  | 6 | 5 | 0 | 2 | 9.01 | Average % area | 10.09 | - | 6.41 | - |
|  |  |  |  |  |  |  | Standard deviation (SD) | 0.53 | - | 0.66 | - |
|  |  |  |  |  |  |  | Coefficient of variation (CV) | **5.25** | **-** | **10.27** | - |
| O |  | 9 | 2 | 0 | 0 | 9.32 | Average % area | 16.31 | 11.37 | 11.11 | 12.93 |
|  |  |  |  |  |  |  | Standard deviation (SD) | 0.31 | 2.58 | 0.85 | 2.88 |
|  |  |  |  |  |  |  | Coefficient of variation (CV) | **1.87** | **22.65** | **7.64** | 22.29 |

**Table Q.** Glycan compositions and proposed structures, average GU values, average relative areas (average % area), standard deviations (SDs) and coefficients of variation (CVs) for the most abundant *N*-glycan structures detected in HCT116 human colorectal cancer cell line from experiment 2 and calculated after triplicate analysis. Structures for *N-*glycans are depicted following the Consortium for Functional Glycomics (CFG) notation: *N*-acetylglucosamine (N; blue square), fucose (F; red triangle), galactose (H; yellow circle), mannose (H; green circle), *N*-acetylneuraminic acid (S; purple diamond). Glycan compositions are given in the terms of hexose (H), *N*-acetylhexosamine (N), deoxyhexose (F), *N*-acetylneuraminic acid (S).

| Peak ID | Possible  structure | Composition | | | | Avg. GU |  | KM12 samples released with: | | | I.M. Data  *(inter-method data)* |
| --- | --- | --- | --- | --- | --- | --- | --- | --- | --- | --- | --- |
|  |  |  |  |  |  |  |  | IS *(n=3)* | PVDF *(n=3)* | N-Hy *(n=3)* |  |
|  |  | Hex | HexNAc | Fuc | Neu5Ac |  |  |  |  |  |  |
| A |  | 2 | 2 | 1 | 0 | 3.71 | Average % area | 2.17 | - | 1.29 | - |
|  |  |  |  |  |  |  | Standard deviation (SD) | 0.09 | - | 0.12 | - |
|  |  |  |  |  |  |  | Coefficient of variation (CV) | **4.19** | **-** | **9.27** | - |
| B | - | 3 | 2 | 0 | 0 | 4.22 | Average % area | 1.57 | - | 16.08 | - |
|  |  |  |  |  |  |  | Standard deviation (SD) | 0.14 | - | 1.52 | - |
|  |  |  |  |  |  |  | Coefficient of variation (CV) | **9.02** | **-** | **9.45** | - |
| C |  | 3 | 2 | 1 | 0 | 4.63 | Average % area | 8.82 | 7.34 | 22.32 | 12.82 |
|  |  |  |  |  |  |  | Standard deviation (SD) | 0.45 | 0.22 | 2.12 | 7.23 |
|  |  |  |  |  |  |  | Coefficient of variation (CV) | **5.09** | **2.99** | **9.49** | 56.38 |
| D |  | 3 | 4 | 1 | 0 | 5.04 | Average % area | 1.04 | - | 1.12 | - |
|  |  |  |  |  |  |  | Standard deviation (SD) | 0.10 | - | 0.24 | - |
|  |  |  |  |  |  |  | Coefficient of variation (CV) | **9.59** | **-** | **21.11** | - |
| E | - | 5 | 1 | 0 | 0 | 5.65 | Average % area | 1.30 | 6.52 | 14.21 | 7.35 |
|  |  |  |  |  |  |  | Standard deviation (SD) | 0.15 | 1.62 | 0.38 | 5.69 |
|  |  |  |  |  |  |  | Coefficient of variation (CV) | **11.23** | **24.80** | **2.70** | 77.40 |
| F |  | 5 | 2 | 0 | 0 | 6.06 | Average % area | 4.44 | 8.85 | 3.22 | 5.50 |
|  |  |  |  |  |  |  | Standard deviation (SD) | 0.30 | 2.01 | 0.30 | 2.77 |
|  |  |  |  |  |  |  | Coefficient of variation (CV) | **6.71** | **22.74** | **9.30** | 50.27 |
| G |  | 6 | 2 | 0 | 0 | 6.97 | Average % area | 13.19 | 10.00 | 7.69 | 10.29 |
|  |  |  |  |  |  |  | Standard deviation (SD) | 0.55 | 1.28 | 0.68 | 2.52 |
|  |  |  |  |  |  |  | Coefficient of variation (CV) | **4.14** | **12.78** | **8.89** | 24.44 |
| H |  | 6 | 2 | 0 | 0 | 7.48 | Average % area | 2.97 | 8.77 | 0.94 | 4.23 |
|  |  |  |  |  |  |  | Standard deviation (SD) | 0.05 | 3.16 | 0.02 | 3.86 |
|  |  |  |  |  |  |  | Coefficient of variation (CV) | **1.83** | **36.08** | **2.01** | 91.25 |
| I |  | 7 | 2 | 0 | 0 | 7.89 | Average % area | 5.75 | 6.97 | 3.16 | 5.29 |
|  |  |  |  |  |  |  | Standard deviation (SD) | 0.10 | 1.58 | 0.39 | 1.87 |
|  |  |  |  |  |  |  | Coefficient of variation (CV) | **1.74** | **22.64** | **12.35** | 35.34 |
| J |  | 5 | 4 | 1 | 1 | 8.11 | Average % area | 8.43 | 11.56 | 4.22 | 8.07 |
|  |  |  |  |  |  |  | Standard deviation (SD) | 0.21 | 2.33 | 0.79 | 3.42 |
|  |  |  |  |  |  |  | Coefficient of variation (CV) | **2.49** | **20.18** | **18.67** | 42.41 |
| K |  | 8 | 2 | 0 | 0 | 8.61 | Average % area | 14.54 | 11.12 | 7.37 | 11.01 |
|  |  |  |  |  |  |  | Standard deviation (SD) | 0.09 | 1.16 | 0.96 | 3.19 |
|  |  |  |  |  |  |  | Coefficient of variation (CV) | **0.65** | **10.43** | **12.99** | 29.02 |
| L |  | 6 | 5 | 1 | 0 | 8.81 | Average % area | 2.65 | 1.76 | 1.29 | 1.90 |
|  |  |  |  |  |  |  | Standard deviation (SD) | 0.30 | 0.74 | 0.15 | 0.72 |
|  |  |  |  |  |  |  | Coefficient of variation (CV) | **11.48** | **41.96** | **11.84** | 38.00 |
| L |  | 5 | 4 | 2 | 1 | 8.81 | Average % area |  |  |  |  |
|  |  |  |  |  |  |  | Standard deviation (SD) |  |  |  |  |
|  |  |  |  |  |  |  | Coefficient of variation (CV) |  |  |  |  |
|  |  |  |  |  |  |  |  |  |  |  |  |
| M |  | 9 | 1 | 0 | 0 | 9.01 | Average % area | 8.64 | 2.21 | 4.64 | 5.17 |
|  |  |  |  |  |  |  | Standard deviation (SD) | 0.63 | 0.88 | 0.18 | 2.87 |
|  |  |  |  |  |  |  | Coefficient of variation (CV) | **7.32** | **39.63** | **3.78** | 55.49 |
| N |  | 9 | 2 | 0 | 0 | 9.31 | Average % area | 21.95 | 17.85 | 11.07 | 16.96 |
|  |  |  |  |  |  |  | Standard deviation (SD) | 0.30 | 1.38 | 0.61 | 4.82 |
|  |  |  |  |  |  |  | Coefficient of variation (CV) | **1.39** | **7.71** | **5.47** | 28.44 |
| O |  | 7 | 6 | 1 | 0 | 9.82 | Average % area | 2.53 | 7.04 | 1.38 | 3.65 |
|  |  |  |  |  |  |  | Standard deviation (SD) | 0.31 | 2.06 | 0.23 | 2.80 |
|  |  |  |  |  |  |  | Coefficient of variation (CV) | **12.19** | **29.25** | **16.75** | 76.60 |

**Table R.** Glycan compositions and proposed structures, average GU values, average relative areas (average % area), standard deviations (SDs) and coefficients of variation (CVs) for the most abundant *N*-glycan structures detected in KM12 human colorectal cancer cell line from experiment 2 and calculated after triplicate analysis. Structures for *N-*glycans are depicted following the Consortium for Functional Glycomics (CFG) notation: *N*-acetylglucosamine (N; blue square), fucose (F; red triangle), galactose (H; yellow circle), mannose (H; green circle), *N*-acetylneuraminic acid (S; purple diamond). Glycan compositions are given in the terms of hexose (H), *N*-acetylhexosamine (N), deoxyhexose (F), *N*-acetylneuraminic acid (S).
